# Quantifying Traces of Tool Use: A Novel Morphometric Analysis of Damage Patterns on Percussive Tools

**DOI:** 10.1371/journal.pone.0113856

**Published:** 2014-11-21

**Authors:** Matthew V. Caruana, Susana Carvalho, David R. Braun, Darya Presnyakova, Michael Haslam, Will Archer, Rene Bobe, John W. K. Harris

**Affiliations:** 1 School of Archaeology, Geography & Environmental Studies, University of the Witwatersrand, WITS 2050, Johannesburg, South Africa; 2 Center for the Advanced Study of Hominid Paleobiology, George Washington University, Washington, DC, United States of America; 3 Interdisciplinary Center for Archaeology and Evolution of Human Behavior (ICArEHB), Universidade do Algarve, Faro, Portugal; 4 Department of Human Evolution, Max Planck Institute for Evolutionary Anthropology, Leipzig, Germany; 5 Universität Tübingen, Abteilung Ältere Urgeschichte und Quartärökologie, Tübingen, Germany; 6 Research Laboratory for Archaeology and the History of Art, University of Oxford, Oxford, United Kingdom; 7 Department of Anthropology, Rutgers University, New Brunswick, New Jersey, United States of America; 8 National Museums of Kenya, Nairobi, Kenya; Universidade do Algarve, Portugal

## Abstract

Percussive technology continues to play an increasingly important role in understanding the evolution of tool use. Comparing the archaeological record with extractive foraging behaviors in nonhuman primates has focused on percussive implements as a key to investigating the origins of lithic technology. Despite this, archaeological approaches towards percussive tools have been obscured by a lack of standardized methodologies. Central to this issue have been the use of qualitative, non-diagnostic techniques to identify percussive tools from archaeological contexts. Here we describe a new morphometric method for distinguishing anthropogenically-generated damage patterns on percussive tools from naturally damaged river cobbles. We employ a geomatic approach through the use of three-dimensional scanning and geographical information systems software to statistically quantify the identification process in percussive technology research. This will strengthen current technological analyses of percussive tools in archaeological frameworks and open new avenues for translating behavioral inferences of early hominins from percussive damage patterns.

## Introduction

Percussive technology is a near ubiquitous feature of the archaeological record and comprises one of the longest-standing traditions of tool use in human evolution. Implements such as hammerstones and anvils have been recorded at some of the earliest Plio-Pleistocene sites including Gona (∼2.6 million years ago [Mya]) [1], Lokalalei 2C (2.34 Mya) [2], Fejej (∼1.9 Mya) [3], Koobi Fora (1.95 Mya) [4], Swartkrans (∼1.9 Mya) [5], Olduvai Gorge (∼1.8 Mya) [6], [7] and Melka Kunture (∼1.7 Mya) [8]–[10]. Percussive tools have also been ethnographically documented amongst the Sotho-speaking and San populations of southern Africa [11]–[13], Native Americans in the Southeastern United States [14], Aboriginal populations in Australia [15], and more recently amongst the people of Langda in Papua New Guinea [16] and the Gamo of Ethiopia [17]. Furthermore, ethological studies have found that some primate species habitually use percussive tools for extractive foraging, including chimpanzees (*Pan troglodytes*) [18]–[21], capuchin monkeys (*Sapajus sp.*) [22], [23] and long-tailed macaques (*Macaca fascicularis aurea*) [24]–[26]. Thus, percussive technology is a common variable between non-human and human primates. Investigating these links in conjunction with evidence from the archaeological record has resulted in new avenues of research [21], [27]–[33]. Based on the pervasive nature of percussive elements amongst almost all primates that use tools extensively [34], recent research has suggested that the oldest technological assemblages are likely to include percussive implements [21], [27], [32]. The use of these tools may have originated during the time of the last common ancestor of chimpanzees and the humans [27], [33]. While questions surrounding the presence or absence of tool using-behaviors in extant primate species and fossil hominins have yet to be solved, the commonality of percussive tool-use in different lineages of primates also indicates the possibility of convergent adaptations.

Nonetheless, before comparisons can be made between the percussive repertoires of extant and extinct species, fundamental issues of identification and analysis in the study of percussive technology must be addressed [4], [7]. While ethnologists and primatologists have the advantage of identifying percussive elements through direct observations of use, archaeologists are faced with the problem of how to detect wear patterns on weathered and fragmented material remains. Identifying percussive implements has historically been based on qualitative observations of usewear patterns [6], [8], [35]–[38], although technological methods have been developed to quantify this process [7], [9], [39]. Nonetheless, reliance on qualitative procedures continues to challenge the progression of technological frameworks. For example, most researchers agree on the definition of stone-knapping hammerstones as rounded (or sub-angular), water-worn pebbles with anthropogenic damage patterns (e.g. pitting and crushing, etc.) typically localized to extremities and/or protruding areas [2], [6]–[8], [40]. However, ambiguous categories such as Leakey's ‘cobblestones’ [6] and Chavaillon's ‘battered pebbles’ [8], both defined by their minimal traces of percussive usewear, are tenuous in their potential for analysis and cross-assemblage comparison. The equivocality of these issues has resulted in the popular use of categorical approaches for analyzing percussive implements that predominantly address questions of typology and/or raw material profiles [1], [3], [41]–[44].

A key problem in advancing technological approaches for percussive technology is that the focus of current research is fixed on whole artefacts as the smallest unit of analysis [7], [9], [21]. This presents concerns when examining percussive implements, such as disentangling empiricist typological fallacies that suggest that each artefact represents a singular, functional use [4], [7]. Furthermore, these studies rely on macroscopic means for analyzing the traces of damage on the surfaces of tools. From this perspective the most non-controversial methodology for identifying percussive tools is through contextual data. The most striking example of this was the use of palaeobotanical remains to substantiate nut-cracking tools in the Acheulean assemblages of Gesher Benot Ya'aqov [45].

Improving the identification and definition of percussive technology from the archaeological record is possible through shifting the analytical lens from the artefacts themselves to individual patterns of damage on the surfaces of tools. This will improve current technological methods in accurately differentiating damage types (e.g., anthropogenic vs. natural) and categorizing usewear types (e.g. pitting vs. crushing). To address these issues, we present a novel three-dimensional morphometric approach for the identification and analysis of percussive damage patterns on the surface of percussive tools. We employ this methodology on experimentally damaged specimens used as hammerstones in knapping experiments. Our analysis also includes naturally pitted specimens to test the ability of this method to distinguish naturally pitted stones from anthropogenic damage. This methodology builds on advances in the characterization of landscapes developed in geomatics [46]–[48]. These techniques allow us to identify percussive features on three-dimensional scans of specimens. Percussion features are objectively identified through a spatial cluster recognition algorithm that identifies significant differences in surface roughness. These features are identified as ‘hot’ and ‘cold’ spots (i.e. area of significantly higher or lower elevation, relative to the immediate area: [49]). We analyze the shape and size of these hot and cold spots (e.g., surface area, volume, perimeter and area). Results show that anthropogenic and natural surfaces have distinct shapes that allow us to develop diagnostic signature criteria [50] for anthropogenic damage patterns.

### Methodological Issues in Percussive Tool Analysis

Percussive technology is one of the few categories of stone-age toolkits that have ambiguous identification criteria. This is largely the result of a paucity of widely agreed upon systematic methods. A critical impediment to concordance in identification is an absence of a uniform terminology. For example, ‘battering’, ‘pitting’, ‘crushing’, ‘pecking’, ‘blunting’, ‘fracturing’, ‘chipped’, ‘ground’ and ‘abraded’ have all been used to describe percussive damage patterns. Yet there is no compendium of objective definitions for these terms. Another challenge for standardizing the description of percussive traces is the confounding influence of the variation of the material properties on stones used in percussive activities. Different materials vary in the way they form and preserve wear patterns. For instance, quartz preserves crushing damage clearly, while pitting is sometimes obscured by angular fractures caused by its trapezohedral lattice structure [51]. Fine-grained igneous rocks tend to preserve most damage patterns, yet the less stable minerals in these lithologies are more likely to weather and obscure them [52].

Much of the description of percussive tools focuses on definitions that are functionally loaded. Isaac, et al. [4] recognized the fallacy of attaching functional definition to tool types. Many percussive artifacts were likely re-purposed during their use-life and/or used as multi-use tools. Discussions surrounding the nature of spheroids have highlighted the multiple uses of percussive tools by documenting that exhausted cores were repurposed as percussive tools [53], [54]. Similarly, hammerstones bearing clear signs of being flaked as cores are also common [7]. Neither is this issue confined to hominin tools: multi-purpose percussive tools used by long-tailed macaques have been identified via both direct observation and use-wear reconstruction [26]. The combination of these difficulties has resulted in a scenario where percussive tools are routinely described using non-diagnostic terminology and the majority of analyses are focused on nominal-scale variables [53].

The difficulties with identifications of this class of artifacts are best emphasized by the inability for researchers studying the early phases of technology to agree on which tools should be considered percussive tools. Studies of the most widely studied Earlier Stone Age assemblage, the FLK “Zinj” floor, shows dramatically different counts for percussive tools (e.g. Leakey [6]: 5.5%; Potts [55]: 1.1%; Mora & de la Torre [7]: 1.6%). Whether these disagreements are the result of differences in terminology or the diagnosis of specimens is difficult to determine. Regardless of the reason, this highlights the inability of researchers to agree on the definition and analysis of this class of technology.

The vagaries of percussive technology description underscore the need for a quantitative and objective methodology for identifying percussive technology. Here we propose a method that identifies percussive damage using a methodology that has been developed and successfully applied in geomatics (albeit on a much larger scale 10^1^ mm^2^ vs. 10^3^ km^2^: [46]). This methodology allows us to investigate damage patterns as an isolated analytical unit on artefacts that can be quantified. The variation in these traces can be compared across objects and subsequently across assemblages. Focusing on individual percussive damage patterns avoids misleading interpretations of artefacts as a singular ‘type’ of tool. In contrast to previous studies, the methodological perspective developed here investigates each isolated instance of damage on a tool as representing distinct percussive events that can be individually analyzed. The techniques and methodological approach developed here is not intended for use in isolation. Percussive technology (like all other types of technology) must be investigated within the temporal, geographic, geological and social context that it was produced in. We see the present methodology as part of a multilayered approach to percussive technology that links individual traces to whole tool characterization within the broader context of the contextual framework that an object is recovered from. The quantitative techniques we describe here are meant to provide objective diagnoses at the smallest scale of analysis. In combination with current categorical and technological approaches this nested series of analytical ‘lenses’ will introduce a new framework for quantifying the identification and analysis of percussive damage patterns. We argue that this combination of approaches is the only way to advance the methods for studying percussive technology.

## Materials

### 3D Morphometric Analysis of Percussive Damage Surfaces

This study of percussive damage patterns comprised 19 archaeological, 20 experimental percussive implements, and 11 naturally pitted cobbles. We limited the study to fine-grained igneous lithologies. We found in experimental conditions that this material type faithfully preserves the largest variety of identifiable damage patterns. However, it should be noted that the exact material composition of experimental and archaeological samples differed, and ideally they should be the same for comparison. However it should be noted that the exact material composition of the experimental and archaeological samples differed (see below for further explanation). Based on previous mechanical analyses of stone artifacts [56], [57], it appears that the major differences in the preservation of percussive damage patterns will be affected by the frequency felsic and mafic elements in the stone. We note that the general similarity in the chemical composition between our experimental and archaeological materials should allow for reasonable comparisons. Furthermore, we plan to expand the methodology to other material to assess the influence of material properties on the results of this analytical technique [52].

Both naturally pitted and undamaged cobbles composed of tholeiitic andesite (Ventersdorp lava) were collected from the Vaal River in the Northern Cape, South Africa. We identified high-energy, conglomeratic deposits to select specimens that exhibited abrasion damage mimicking anthropogenic percussive damage. Undamaged cobbles were used as experimental hammerstones in one-hour stone-knapping trials by novice, intermediate and experts to generate characteristic percussive damage patterns. Although the intensity of damage patterns was dependent on the experience of the knapper, all experimental percussors displayed damage patterns characteristic of use as hammerstones.

The archaeological sample was selected from early Pleistocene and Holocene collections from the Koobi Fora Formation and the Galana Boi Formation, Kenya. All archaeological artefacts were accessed and studied at the National Museum in Nairobi, Kenya. The Early Pleistocene artifacts derive from archaeological sites form the KBS and Okote members of the Koobi Fora Formation (∼1.6 Ma) [4], [58]. In particular, we selected artifacts from the FxJj 10, FxJj 11, FxJj 16 and FxJj 18GS assemblages. Stones that exhibited percussive damage from the Galana Boi Fm. were selected from the assemblages of FxJj 12N, GaJi 12, and GaJi 4. The Holocene samples derive from sediments that are between 9000 and 4500 yrs BP [59]–[61]. All artifacts are made of fine-grained tholeiitic basalt. This is the dominant raw material in the Koobi Fora and Galana Boi Fms. [58], [60]. These tools were previously identified as stone-knapping hammerstones due to their resemblance with experimental collections. A small sample (N = 2) was also included from a survey of deposits from the Koobi Fora Fm. (Lonyumun to Burgi members) that are older than 2.2 Ma. These are referred to as the PrimArch sample as they were part of a larger study of Primate Archaeology [33]. Table 1 outlines specimens, their provenance, and the damage patterns that are visible in hand sample identification.

**Table 1:** Early Pleistocene, Holocene and PrimArch artefacts examined.

Site	Accession #	Usewear Patterns	Formation or Member	Depositional Context
FxJj 10	553	C, P	KBS	Sandy Tuff, Floodplain
FxJj 11	74	C, P	Okote	Yellow Orange Tuff
FxJj 16	400	C, P		
FxJj 16	408	C, P, FS		
FxJj 16	543	C, P		
FxJj 16	549	C, P, F	Okote	Sandstone, Channel Fill
FxJj 16	552	C, P		
FxJj 16	580	C, F		
FxJj 16	587	C, P, F		
FxJj 16	680	C, P		
FxJj 18GS	130/26	C, P		
FxJj 18GS	2148	C	Okote	Sandy Floodplain
FxJj 18GS	5305	C, P		
FxJj 18GS	5715	P		
GaJi 4	1363	C, P		
GaJi 12	1433	C, P	Galana Boi	
GaJi 12	3105	C, P, F		Beach Sand
GaJi 12	1269	P, F	Galana Boi	
FxJj 12N	170	C, P, FS	Galana Boi	
PrimArch	1206	P	Tulu Bor	Sand
PrimArch	1263	P	Tulu Bor	

Usewear patterns abbreviated as follows: C =  Crushing, P =  Pitting, FS  =  Flake Scarring, & F =  Fracturing.

## Methods

### 3D Morphometric Analysis of Percussive Damage Surfaces

A NextEngine 3D laser scanner was used to capture the surface texture on all objects included in this study. Three-dimensional representations of the surfaces were converted into scan meshes with a resolution of 17–40,000 points per in^2^. Experimental specimens were selected based on the presence of two types of anthropogenic damage: crushing and pitting. We defined crushing damage as abraded and roughened surfaces localized on extremities of specimens. Pitting damage is defined as clusters of small cupules or divots often associated with crushing damage also localized on extremities of stone surfaces. Natural abrasion was defined as any damage (typically pits) mimicking anthropogenic damage patterns resulting from fluvial processes.

**Figure 1:**
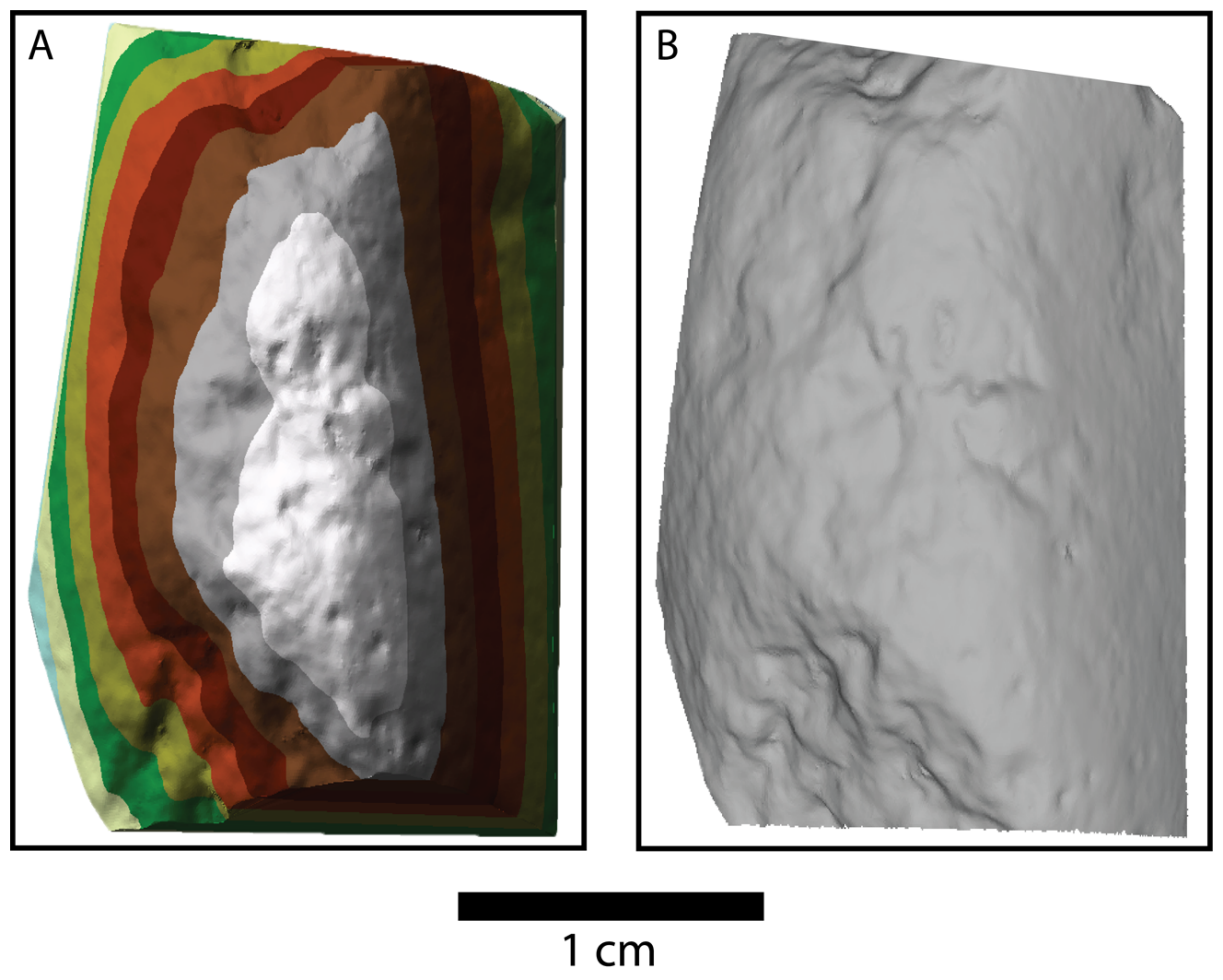
A three-dimensional model of one of the fine grained igneous experimental hammerstones used in this study. A) a TIN model of the surface scan of this experimental hammerstone; B) a hillshade model of the same scan as in A, highlighting the percussive damage on the surface of this experimentally made hammerstone. Notice the difference in surface roughness between the left side versus the right side of the scan.

**Figure 2:**
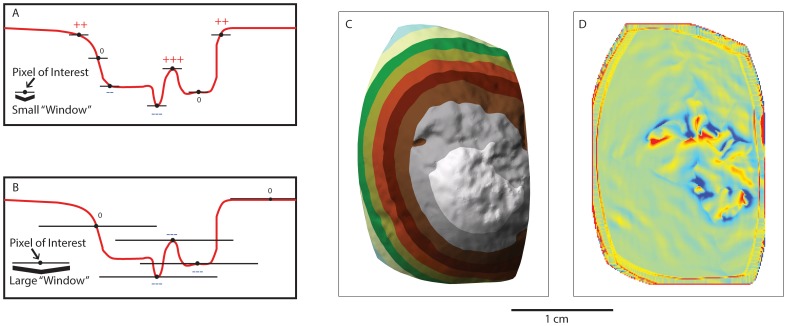
A schematic representation of the topographic position index. This shows a schematic description of the TPI index on two identical surfaces with a small window size (A) and large window size (B). The window size changes the resolution of identifying high values (peaks) versus low values (valleys) of the TPI statistic (redrawn from Jenness 2002). C) This is an experimentally damaged percussive tool showing elevation values represented by a TIN model. D) This is the TPI index of the same specimen. Note the edge effects around the borders of the TPI model.

**Figure 3:**
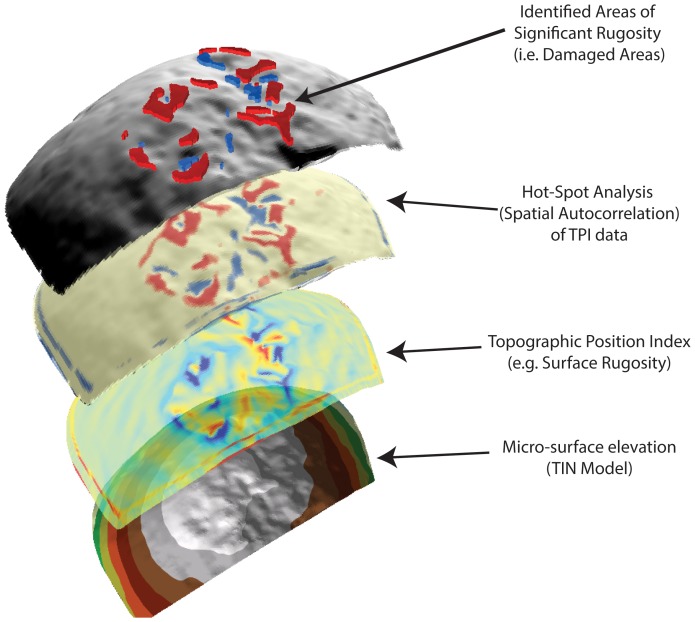
A schematic representation of the full analysis that identifies the statistically significant (spatially auto-correlated) areas of percussive damage. The three dimensional nature of this representation prohibits the use of a scale, however this is the specimen that is represented in Figure 2 and as such provides an estimate of the overall size of the damaged area.

We conducted the analysis of the 3D scans using ArcGIS 10.2 due to the wide scale availability of this software package and its ability to perform numerous spatial clustering algorithms (see SI). The ESRI suite of GIS software has recently been applied in several archaeological studies (e.g. [62]–[65]). Scan meshes were imported as TIN models using a linear interpolation method (Fig. 1), which were then used to create a series of digital elevation models. These models of the surface micro-topography were analyzed to determine areas of high micro-topographic roughness in a topographic position index (TPI) [46]. As a result we focused our analysis on patterns elucidated by the topographic position index. This index was particularly well suited for measuring the micro-topographic rugosity of percussive damage patterns because it is not affected by the curvature of the scan mesh. We used a Hot Spot Analysis (Getis-Ord GI*) to identify spatial autocorrelation. This produces an overlay highlighting cells with clustered groups of high (peaks-red) or clustered low (valleys-blue) TPI values (Fig. 2). The Getis-Ord* statistic allows us to identify cells that are significantly clustered spatially when compared to a random distribution of mean values (Fig. 3) [49]. We identified damage patterns based only on those cells that were clustered above the p<.001 level (i.e. the clustering of values at a level that far exceeds the expectations of a random pattern). This provides an objective method of identifying locations where particularly rugose areas are highly clustered (i.e. damaged surfaces; Fig. 3).

Hot spot analyses of experimental and archaeological scan meshes should produce tightly clustered hot and cold spots in areas corresponding to percussion related damage patterns. When surfaces are battered in percussive activities, microscopic conchoidal fracture patterns create pits and ridges resulting in roughened textures that should be highlighted by the TPI metric. If those pits and ridges are clustered they should be identified by the spatial auto-correlation (i.e. Hot Spot) analysis. Hot spot patterns were transformed into polygons that generated metric data (volume, surface area, maximum length and width, etc.), which are statistically compared below.

## Results

### 3D Morphometric Analysis of Percussive Damage Surfaces

The Hot Spot analysis (Getis-Ord*) function highlighted areas on scan meshes with statistically significant clusters of high and low elevation. This corresponded to areas that we had identified as experimentally produced anthropogenic damage and natural abrasion on stone surfaces (Fig. 3). This demonstrates the use of geomatic techniques in strengthening technological frameworks for identifying percussive artifacts.

**Figure 4:**
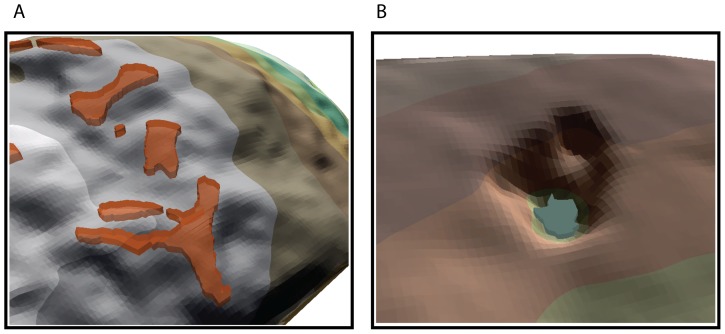
Three-dimensional representations of two polygons identified by the hotspot analysis. The polygons have been draped over the three-dimensional surface of these specimens. A) Represents a hot spot identified on an experimental hammerstone B) Represents a hot spot on a naturally damaged cobble.

Here we provide data on the morphology and shape of the hot spot polygons from the specimens in this study. Statistical analyses of the size and shape of hot spot polygons indicated significant differences between anthropogenic damage patterns and natural pitting damage. We chose to focus on the perimeter to volume ratio of polygons of particularly hot spots (i.e. peaks). The rationale for this investigation is that we expect natural damage to have rounded perimeters, and deeper relatively smooth bases. Both of these expected features of natural damage would increase the volume and decrease the relative perimeter (i.e. jagged perimeter lengths will always be larger than smooth ones; Fig. 4). Our investigation of percussive damage indicates that it produces numerous small-scale peaks as the crystalline structure of the stone is crushed.

Although we believe the ratio of perimeter to volume ratios will distinguish between natural and anthropogenic damage, it is necessary to transform these measures because of allometric affects. Volume necessarily increases in three dimensions (x, y, z) while perimeter can only increase in two dimensions (x, y). Thus small objects necessarily have larger perimeter to volume ratios. To accurately compare ratios of these two variables, we squared perimeter values prior to calculating the ratio between perimeter and volume values.

**Figure 5:**
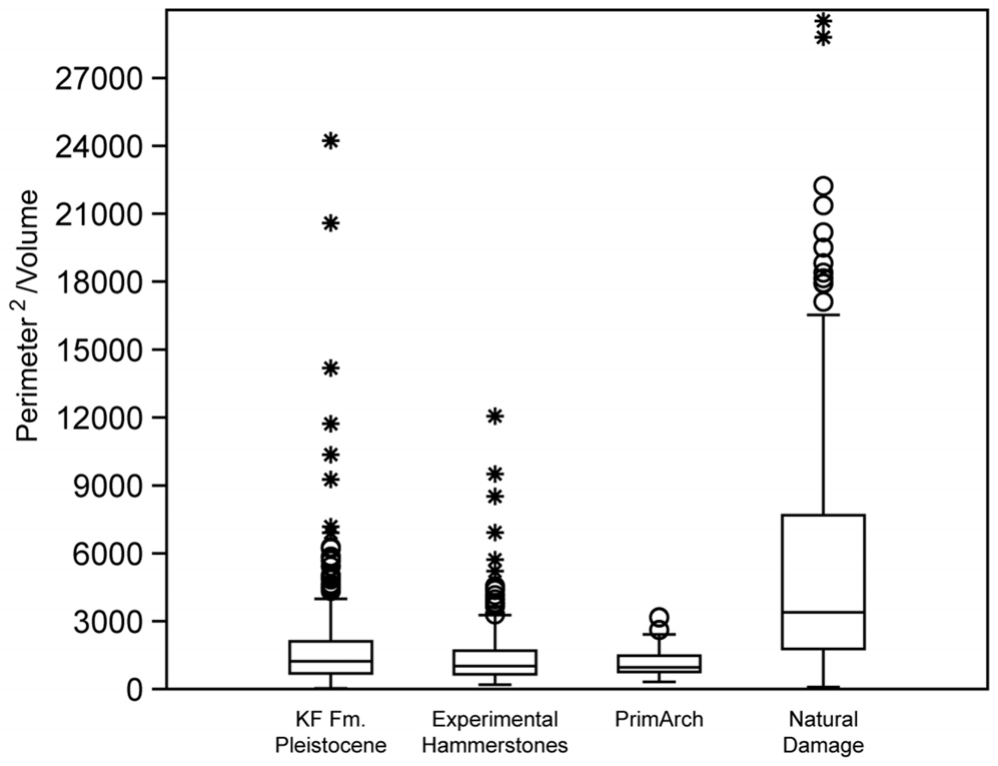
Box-plot of hotspot polygon perimeter to area ratio values. Boxes represent the interquartile range of values for the four samples (archaeological specimens from Pleistocene deposits of the KF Fm.; experimental percussive specimens; PrimArch specimens from the KF Fm. (>2.2 Ma); and naturally damaged specimens).

Mann-Whitney U tests (with Bonferoni corrections to account for multiple tests) identified significant differences in the perimeter to volume ratio between experimentally produced percussive damage (N = 189) and naturally damaged surfaces (N = 183; p = 0.0001). In addition, subsequent Mann-Whitney tests identified significant differences between the surfaces of archaeological specimens (N = 499) and naturally damaged surfaces (p = 0.0001). No significant differences were found between experimental and archaeological groups (p = 0.2052) (Fig. 5).

**Figure 6:**
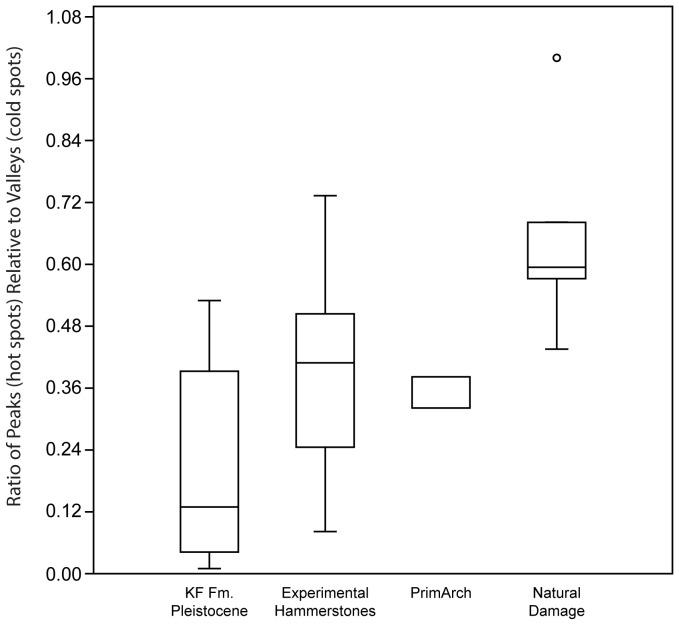
Box-plot of the ratio of hot spot (peaks) polygon area relative to cold spot (valleys) polygon area. Boxes represent the interquartile ranges for the four samples (archaeological specimens from Pleistocene deposits of the KF Fm.; experimental percussive specimens; PrimArch specimens from the KF Fm. (>2.2 Ma); and naturally damaged specimens).

The second approach that we investigated regarding the quantification of the Hot Spot analysis (Getis-Ord*) is associated with the relative amount of hot spots (peaks) relative to cold spots (valleys). The rationale behind this analysis is that naturally damaged specimens tend to develop indentations that are rounded with smooth bases. As a result they tend to make cold spots (valleys) with limited hot spots (peaks) around the edge of these cold spots. In contrast, the Hot Spot analysis (Getis-Ord*) detects fewer cold spots on anthropogenic scan surfaces because the roughened floors of pits developed during percussive activities create ridges that limits the clustering of low elevation values. We investigated the average area ratio of hot spots to cold spots to test this. Mann Whitney tests identified the significant differences between experimentally damaged specimens and naturally damaged pieces (Mann Whitney U: 14; p<.001; Fig. 6). Archaeological specimens also showed significant differences from the naturally damaged sample (Mann-Whitney U: 39; p<.001). As this analysis investigates the entire damaged surface as a single unit, sample sizes are much smaller (KF Fm. Pleistocene: N = 20; KF Fm. PrimArch: N = 2); however the patterns appear to be robust. The smooth floor of natural pitting creates areas of low elevation values that are clustered together in a larger area of cells (see Fig. 6). These data corroborate statistical evidence for quantitatively differentiating anthropogenic damage from natural abrasion damage.

## Discussion

### Distinguishing Anthropogenic Damage from Natural Abrasion Damage

**Figure 7:**
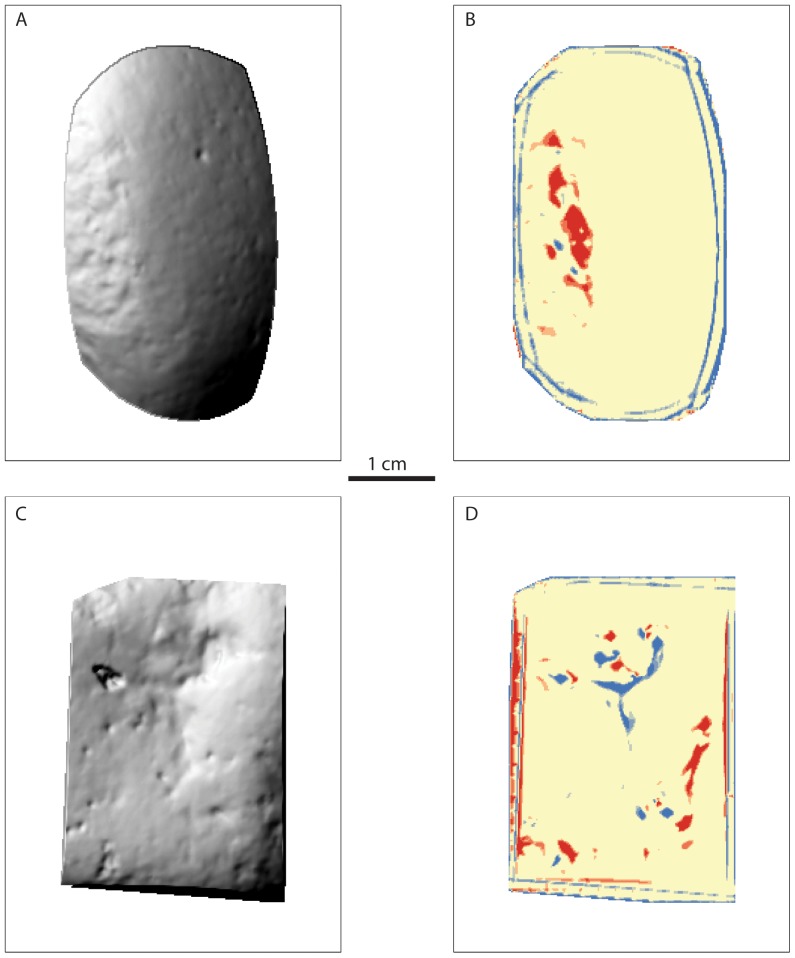
Contrasting spatial autocorrelation analysis (e.g. Hot Spot Getis-Ord*) of an experimentally produced hammerstone (A–B) and a naturally damaged specimen (C–D). Images A and C represent a hillshade representation micro-topography values.

This methodology demonstrates the application of geomatic techniques in distinguishing anthropogenic damage patterns from natural abrasion damage. Creating polygons from spatial auto-correlation analysis has provided a new means of quantifying wear on percussive implements and to differentiate them from natural damage. Our findings on the contrast of polygon volume between archaeological/experimental and natural groups relates to the roughness of scan surface textures mapped during TPI generation. Scans of archaeological and experimental specimens produce hot spot clusters based on roughened, micro-fractured surfaces that emphasize peak values, while the smoothed surfaces on natural scans contrast differences between peaks and valleys (see Fig 7). As a result, the polygons produced on natural scan surfaces have more depth and thus volume compared to archaeological and experimental polygons.

These findings are corroborated by polygon area data, where again significant differences between archaeological/experimental and natural groups are evident. In this case, the sheer number of polygons on natural scan surfaces in relation to increased volume produced more area per polygon when compared to archaeological and experimental groups. These findings provide clear evidence that geomatic analysis can be used to detect subtle differences in the roughness of scan surfaces using TPI and Hot Spot (Getis-Ord*) techniques. Furthermore, continued testing of this method will aim to distinguish between damage patterns, e.g. crushing versus pitting, as well as the differences in detecting damage pattern preservation on different stone types (e.g. quartz versus lavas, etc. [52]). As mentioned above, manipulating moving window sizes can alter the amount of variation in surface roughness identified during the Hot Spot analysis (Getis-Ord*). The goal of future research is to: 1) tailor moving window sizes for tracing damage patterns in specific conditions (e.g. detecting pitting damage on quartz material); and 2) to apply this technique to damage produced during different percussive activities (e.g. nut-cracking vs. stone-knapping, etc.).

## Implications

As the study of percussive tools continues to bridge the gap between archaeology and primatology, narrowing the focal lens from macro- to microscopic detail will increase our confidence regarding their evolutionary significance. However, critical questions including the relationship between structural properties of rock types and damage patterns, as well as differentiating tool use based on these patterns (e.g. stone-knapping vs. bone-breaking, etc.) are essential for assessing such implications. Building a repertoire of standardized analytical techniques will create opportunities for inter-assemblage comparisons of percussive tools, which is currently lacking in Paleolithic archaeology. This is critical for constructing an evolutionary sequence of percussive toolkits that may highlight trends in their use over time.

Understanding evolutionary trends will create further opportunities to provide insight into the large questions surrounding the significance of percussive technology, including the origins of lithic technology and the behavioral repertoire of the last common ancestor (LCA) of *Pan* and *Homo* [21], [32], [33]. As mentioned above, the ubiquity of percussive tool use amongst the Primate Order may argue for an ancient evolutionary trajectory that may have developed with the LCA. Investigating the relationship between percussive implements and the origins of tool use has recently sparked interest in archaeological and primatological research agendas [21], [27], [66]. The PrimArch survey material analyzed above was collected during current research ongoing in the Koobi Fora Formation attempting to locate archaeological sites spanning beyond the dates of the current record at ∼2.6 Mya. However, this research faces significant challenges such as disentangling taphonomic processes from anthropogenic traces of percussive damage. The methodology outlined here may provide the means for differentiating damage patterns resulting from pounding activities from natural abrasion. While evidence of pre-Oldowan industries is currently controversial [67], interest in their archaeological traces may eventually lead to material evidence of tool-use that extends beyond the earliest Pliocene (cf. [68]). Thus, it is necessary to develop methodologies that will address evolutionary-scale questions about the role of percussive technology in the origins of hominin technology.

## Conclusions

Despite continued interest in percussive technology [7], [21], [28], [39] the identification of these tools is fraught with difficulties. Statistical quantification of percussive damage creates an opportunity to identify and analyze behavioral events preserved on Plio-Pleistocene percussive implements. This has important implications for improving technological frameworks in terms of lessening the dependency on qualitative observation and strengthening qualitative analyses. Our computer-aided methodology improves on this process.

The object of the methodology described here is to complement existing technological analyses and to shift from analyses that investigate the artifact as a whole to more focused analyses on individual wear patterns. To best model this process, we envisage the identification and analysis of percussive implements as a series of nested lenses that tighten their focus from the context of discovery to the analysis of individual damage patterns. The broadest lens identifies the exact geological context of artifacts that may preserve percussive damage. This requires a detailed understanding of the microstratigraphy of localities where each specimen was recovered. Artifacts found in surface contexts are especially troubling because they are subject to continual erosional forces that might obscure possible damage patterns or worse yet acquire new damage patterns that are difficult to distinguish from anthropogenic patterns. Thus when identifying the earliest traces of tool use we should limit our inferences to *in situ* artifacts which are more straightforward for identifying anthropogenic damage patterns because of their geological context. The next level of focus in the analysis of percussive tool use is macroscopic investigations involving metric measurements (length, width, height, weight, mass, surface area, volume, etc.) and the observation of possible damage patterns (i.e. crushing, pitting, fracturing, flake scars, etc.). This narrows typological classification of artifacts (e.g. [26], [39]), and has been successfully demonstrated in recent archaeological and primatological studies analyzing percussive wear patterns in relation to tool types [26], [69]. Finally, the smallest scale analysis described here uses 3D technology and geomatic analysis to focus on individual damage patterns and quantify surface textures to distinguish them from natural abrasion damage. The development of these analytical processes will aid in standardizing a comprehensive methodology for addressing issues on the evolution of percussive technology.

## Supporting Information

Text S1 **Contains an outline of the GIS procedures and additional discussion of the methodology.**
(DOCX)
