# Supplementary material for: Quantifying Traces of Tool Use: A Novel Morphometric Analysis of Damage Patterns on Percussive Tools
Source: PLoS One. 2014 Nov 21;9(11):e113856. doi: 10.1371/journal.pone.0113856 (PMC4240665; doi:10.1371/journal.pone.0113856)
Supplement: Text S1 — Contains an outline of the GIS procedures and additional discussion of the methodology. (DOCX) [file pone.0113856.s001.docx]

**Text S1: Supporting Information**

Quantifying Traces of Tool Use: A Novel Morphometric Analysis of Damage Patterns on Percussive Tools

Matthew V. Caruana, Susana Carvalho, David R. Braun, Darya Presnyakova, Michael Haslam, John W. K. Harris, Will Archer, Rene Bobe

This document describes the procedures used in ArcGIS^®^ 10.2. to run the Hot Spot Analysis (Getis-Ord*), how to calculate measurements of Hot Spot polygons and all polygon data are provided as well. Scan meshes produced by a 3D laser scanner were cleaned so that the only the damaged portion of the specimen was visible using MeshLab™ 1.3.2. Scanned meshes were saved as .xyz files so that they may be imported into ESRI ArcGIS 10.2 using the 3D Analyst™ extension. To insure that our subjective identification of the visible damage pattern was not unduly influencing the analysis, we also preserved several “undamaged” surfaces and analyzed these surfaces to determine if our technique would produce false positive identifications. The analyses in this study employ techniques usually applied in geomatics. As such, they are designed for investigating landscapes, which are often represented as raster datasets or TIN (triangulated irregular networks) models. In raster datasets, each pixel represents a certain area of the landscape and the value of that pixel reflects something about that space (often elevation in digital elevation models). TIN models are based on a series of triangles connecting nodes. The nodes reflect some value of the landscape (often elevation). In these types of datasets each pixel or node can only have a singular value. In other words, areas whereby a single location has multiple elevation values (e.g. an overhang on a cliff face) are difficult to represent. We needed to make sure that the damaged surface was represented in a manner where the entire damaged surface was parallel to the visible plane (i.e. the z-value was oriented towards the viewer). Raster data sets were created from the TIN models using a linear interpolation method. These raster models represent elevation maps of microscopic variation (pixels ≤.13 mm) of the surface of a percussive tool. We chose to use an algorithm that evenly spaced cells along the surface of an object. However, we also ran the analysis using a standardized cell size. As the results of the two techniques are identical we only provide data based on rasters with evenly spaced cells.

We then used these rasters to calculate a topographic positioning index (TPI), which was developed to highlight landscape corridors [46]. We then used the Hot Spot Analysis (Getis-Ord*) to statistically verify the clustering of high (hot spots) and low (cold spots) elevation values that corresponded to percussive damage patterns on mesh surfaces. The hot and cold spot patterns were then transformed into polygons that produced data sets on metric aspects of polygons patterns (e.g. volume, surface area, maximum length and width, etc.).

We expect that the shape, size, and nature (frequency of hot vs. cold or peaks vs. valleys) of the polygons from naturally damaged specimens will be significantly different from those of the experimentally damaged specimens. When river cobbles are naturally damaged in fluvial setting we expect that exposure to suspended load material and rolling action in riverbeds will continuously weather damage surfaces. The effect will be a relatively smooth surfaces caused by constant abrasion. This is unlike the roughened character of percussive damage. Percussive damage on artefacts in archaeological contexts is less likely to be weathered in a similar manner. As such, we expect to find that hot and cold spot polygons in the natural damaged sample to be deeper and relatively smooth at their base. As a result these polygons will have higher volume relative their perimeter values when compared to the roughened surfaces of the experimental percussive sample. Nonetheless, secondary fluvial contexts in archaeological sites may complicate preservation circumstances and weather percussive damage patterns post-depositionally. Therefore, archaeological specimens may exhibit patterns similar to either experimental or natural samples, but may also exhibit patterns that are not similar to either. To account for these factors, our archaeological sample was derived from sites with minimal post-depositional disturbance [4] in an effort to limit these potentially confounding variables.

**PART I. GIS METHODOLOGY**

The structure of this section provided is numbered in sequential steps, the first point (+) outlining how to navigate ArcMap^®^ and the following points (-) outline any settings that need to be changed from default options. The last point (=) lists the type of output file.

*Topographic Positioning Index (TPI) and Hot Spot Analysis (Getis-Ord*)*

1. Import ASCII 3D file

+3D Analyst Tools > Conversion > From File > ASCII 3D to Feature Class

-Input = .xyz file

-Output Feature Class Type = ‘POINT’

=Feature class points

2. Create TIN Model from Feature Class

+3D Analyst Tools > Data Management > TIN > Create TIN

-Input Feature Class = Feature class points

=TIN model

3. Create Raster from TIN

+3D Analyst Tools > Conversion > From TIN > TIN to Raster

-Input TIN = TIN model

-Sampling Distance = ‘CELLSIZE 0….’

=Raster layer

4. Create DEMs from Raster

+Spatial Analyst Tools > Neighborhood > Focal Statistics

-Create Smooth DEM:

-Input Raster = Raster layer

-Neighborhood Settings:

•Height = 10

•Width = 10

-Statistics Type = ‘MEAN’

=SmoothDEM

-Create Max & Min DEMs

-Input Raster = SmoothDEM from Raster

-Neighborhood Settings:

•Height = 3

•Width = 3

-Statistics Type = ‘MAXIMUM’ (for MaxDEM) & ‘MINIMUM’ (for

MinDEM)

=MaxDEM & MinDEM

5. Create TPI

+Spatial Analyst Tools > Map Algebra > Raster Calculator

-Create formula by double-clicking symbols and file names =

(SmoothDEM – MinDEM) / (MaxDEM – MinDEM)

=TPI

6. Overlay Point Grid

+Conversion Tools > From Raster > Raster to Point

-Input Raster = TPI

=Point grid

7. Hot Spot Anlaysis (Getis-Ord GI*)

+Spatial Statistics Tools > Mapping Clusters > Hot Spot Anlaysis (Getis-Ord

GI*)

-Input Feature Class = Point grid

-Input Field = ‘GRID_CODE’

=Hot Spot overlay

*Creating Hot Spot Polygons*

1. Create TIN from Hot Spot Layer

+3D Analyst Tools > Data Management > Create TIN

-Input = Hot Spot overlay

-Height Field = ‘GI_BIN’

=Hot Spot TIN model

2. Create Contours

+3D Analyst Tools > Triangulated Surface > Surface Contour

-Input Surface = Hot Spot TIN model

-Contour Interval = 1

-Base Contour = -3

=Contour feature

3. Set Measurement Units

+Go to ‘View’ tab in Menu Bar

-Data Frame Properties

-Go to ‘General’ tab

-Set ‘Map’ & ‘Display’ options as ‘Millimeters’

-Press ‘Apply’ & ‘OK’

4. Select Red Contours

+Go to ‘Selection’ tab in Menu Bar

-Select by Attributes

-Layer = Contour feature

-Select ‘Get Unique Values’

-In ‘Method’ box:

+Double-click ‘Contour,’ ‘=,’ ‘2’

-Click ‘Apply’ & ‘OK’

5. Create Red Contours

+Right-click on Contour File in the Workflow Column on the left side of the

screen

-Select ‘Data’ > ‘Export Data’

-Save as New Layer

=Red contour feature

6. Select Blue Contours

+Go to ‘Selection’ tab in Menu Bar

-Select by Attributes

-Layer = Contour from TIN

-Select ‘Get Unique Values’

- In ‘Method’ box:

+Double-click ‘Contour,’ ‘=,’ ‘-3’

-Click ‘Apply’ & ‘OK”

7. Create Blue Contours

+Right-click on Contour File in the Workflow Column on the left side of the

screen

-Select ‘Data’ > ‘Export Data’

-Save as New Layer

=Blue contour feature

8. Isolate Hot Spot Contours (i.e. Remove *Edge Effect*)

+Go to ‘Editor’ tab in Menu Bar

-Turn Editor on

-Find ‘Select Features’ in Tool Bar (below the Menu Bar)

-Select ‘Lasso’

-Use Lasso to select all relevant Hot Spots, excluding *edge effect* Hot Spots

9. Eliminate Extraneous Contour Data

+Right-click on Red and Blue Contour files in Workflow Column on the left

side of the screen

-Select ‘Open Attribute Table’

-Select ‘Switch Selection’ in Tool Menu of Attribute Table Window

-Select ‘Delete’ in Tool Menu of Attribute Table Window

-Go to ‘Editor’ tab in Menu Bar

-Turn Editor off

10. Create Red and Blue Polygons

+Data Management Tools > Features > Features to Polygons

-Input = Red or Blue Contour features, respectively

=Red & Blue Polygons

11. Add Surface Information

+3D Analyst Tools > Functional Surface > Add Surface Information

-Input = Red or Blue Polygons, respectively

-Input Surface = TIN Model from 3D .XYZ Feature (*This is the original TIN

model created in PART I)

-Output Property = ‘Z-MEAN’ & ‘SURFACE AREA’

12. Calculate Polygon Volume

+3D Analyst Tools > Triangulated Surface > Polygon Volume

-Input = TIN Model from 3D .XYZ Feature (*This is the original TIN model

created in PART I)

-Input Feature Class = Red or Blue Polygon files, respectively

-Height Field = ‘Z-MEAN’

-Reference Plane = ‘BELOW’ for Blue Polygon file & ‘ABOVE’ for Red

Polygon file

-Surface Area Field = ‘SArea2’

13. Calculate Perimeter & Area for Polygon Files

+Right-click on Red and Blue Polygon files in the Workflow Column on the

left side of the screen

-Select ‘Open Attribute Table’

-Click dropdown menu in ‘Table Options’ tab in Tool Bar of Attribute Table

Window

-Select ‘Add Field’

-Name Field = ‘Perimeter’ or ‘Area,’ respectively

-Type = ‘FLOAT’

+Precision = 10

+Scale = 5

-Right-click on Perimeter and Area name fields on top of Attribute Table

-Select ‘Calculate Geometry’

-Select corresponding measure for field

*Attribute Tables for Red and Blue Polygon files can be exported in .txt file formats. Find ‘export’ in Attribute Table options.

**PART II. HOT SPOT ANALYSIS PARAMETERS**

*Cell Sizes*

Cell sizes are defined during the raster creation step in Part I, and are automatically calculated from TIN models. We found that larger cell sizes (≥0.5mm) will distort and pixelate the overlay produced during the Hot Spot analysis, while smaller cell sizes (≤0.001mm) will render the neighborhood comparison insufficient. However, the cell size generated during the TIN-to-Raster conversion (within a range of 0.01 to 0.03mm) will not significantly affect the Hot Spot outcome.

*Moving Window Sizes*

The size of the moving window used during the generation of the TPI will affect the Hot Spot analysis in terms of the number of cells compared to one another. Using a smaller moving window size, such as a 5x5 will produce a smaller scale of analysis and a finer resolution in detecting significant clusters of elevation variation. We chose to use a 10x10 neighborhood size because it captures a larger comparison range of cell values spread across entire scan surfaces, and because the raw material types preserved easily detectable usewear patterns.

*Limitations*

There are certain limitations in using the methodology described above, one of the most critical being the interpretability of 3D scans into landscape surfaces in GIS software. Problems arise when scan surfaces overlap and overlay multiple points, resulting in multiple elevation values for a single location. This creates complications when these points are translated into cells during raster creation. Also, damage pattern preservation on varying raw material types may be difficult to capture using a laser scanner. Some reflective materials may be incompatible with this type of analysis. Most importantly, false edges are created in the TPI analysis during the raster generation process, a factor that we refer to as ‘edge effect.’ As the moving window calculates scores along the edges of scan meshes, part of the window overshoots the edge of the scanned surface and calculates the values of missing cells as 0. This gives the impression of a dramatic drop in elevation. As a result erroneous hot and cold spot patterns are generated along the edges of scan meshes. These are easily detected as running parallel to the edges of scans. They are easily removed prior to analysis of the size and shape of polygons (as was done here), although this does represent one subjective component of the analysis.

However, there are some solutions to these difficulties. Overlapping mesh surfaces can be trimmed prior to analysis to remove multiple elevation values for a single location on the mesh. Varying the moving window size (in the TPI analysis) can aid in detecting fluctuations in elevation patterns on varying scales, such that using a smaller size (e.g. 5x5) can detect smaller variations in elevation values. This will produce a TPI raster with finer resolution for capturing small-scale percussive damage patterns on scan surfaces. Finally, the “edge effect” described earlier can be completely eliminated during the generation of polygons, where hot spot contours can be selected in exclusion of those falsely produced during the TPI phase.

**PART III. POLYGON DATA TABLES**

**Archaeological Polygon Data**

**Red Polygons**

| **Accession#** | **Z_Mean** | **SArea** | **Volume** | **SArea2** | **Perimeter** | **Area** |
| --- | --- | --- | --- | --- | --- | --- |
| FxJj10-553 | 71.08788713 | 0.398733131 | 0.004434951 | 0.186497142 | 2.36564 | 0.33063 |
| FxJj10-553 | 72.19088141 | 1.612071461 | 0.026698616 | 0.985890457 | 4.22806 | 0.88461 |
| FxJj10-553 | 73.11350084 | 0.121315727 | 0.000821656 | 0.061067355 | 1.44212 | 0.11704 |
| FxJj10-553 | 71.7912893 | 0.197405997 | 0.001113166 | 0.098500585 | 1.38442 | 0.13187 |
| FxJj10-553 | 72.60177834 | 3.104810203 | 0.050710947 | 1.719950306 | 5.56989 | 1.63417 |
| FxJj10-553 | 72.86947934 | 7.082425077 | 0.071516175 | 3.761069932 | 11.05796 | 3.9045 |
| FxJj10-553 | 73.19757601 | 0.047731672 | 0.000187526 | 0.022148525 | 0.74215 | 0.03251 |
| FxJj10-553 | 73.09065887 | 3.296391441 | 0.035828015 | 1.607833425 | 7.4234 | 1.78128 |
| FxJj10-553 | 73.24822968 | 13.57781405 | 0.211145174 | 8.175512182 | 24.36562 | 10.83023 |
| FxJj10-553 | 73.08928222 | 0.107637094 | 0.000538055 | 0.053379648 | 1.24861 | 0.10031 |
| FxJj10-553 | 72.6191279 | 2.102997616 | 0.01468571 | 1.039637142 | 4.29372 | 0.98501 |
| FxJj10-553 | 72.5682142 | 0.511242825 | 0.002189238 | 0.211405921 | 2.21279 | 0.26144 |
| FxJj10-553 | 72.0134691 | 1.617282233 | 0.011606086 | 0.92109375 | 4.29228 | 1.04166 |
| FxJj10-553 | 72.89332992 | 2.773010775 | 0.04649373 | 0.997574571 | 7.40283 | 1.55254 |
| FxJj10-553 | 72.93236975 | 0.146307502 | 0.001276825 | 0.07281668 | 1.34526 | 0.08776 |
| FxJj10-553 | 71.89208542 | 0.405074688 | 0.004519935 | 0.203329462 | 2.24653 | 0.29858 |
| FxJj10-553 | 72.20384522 | 0.439287581 | 0.007584229 | 0.216124739 | 2.29433 | 0.33951 |
| FxJj10-553 | 73.13639715 | 0.456477117 | 0.00390473 | 0.2681929 | 2.08995 | 0.28701 |
| FxJj10-553 | 72.80761684 | 1.933199245 | 0.019679344 | 0.981276878 | 4.35371 | 1.03227 |
| FxJj10-553 | 72.81301659 | 1.249263125 | 0.011108211 | 0.646233511 | 3.63865 | 0.68544 |
| FxJj10-553 | 70.51789998 | 1.183996433 | 0.021070335 | 0.730051024 | 4.40554 | 1.0365 |
| FxJj10-553 | 72.97713755 | 10.46865886 | 0.466137971 | 7.78780639 | 22.24469 | 6.78173 |
| FxJj10-553 | 69.9209418 | 0.150521737 | 0.002618252 | 0.062859675 | 1.57857 | 0.11332 |
| FxJj10-553 | 71.11397816 | 0.319458786 | 0.003222257 | 0.165417492 | 1.70077 | 0.17832 |
| FxJj10-553 | 71.00901469 | 0.365474143 | 0.007446694 | 0.150108923 | 2.52051 | 0.26886 |
| FxJj10-553 | 70.15347351 | 6.911986655 | 0.873145745 | 3.362873776 | 19.4895 | 6.17249 |
| FxJj10-553 | 70.76416004 | 0.33584587 | 0.003456717 | 0.162394064 | 1.77974 | 0.22847 |
| FxJj10-553 | 69.15935047 | 0.40250841 | 0.014472107 | 0.202406006 | 2.31318 | 0.35109 |
| FxJj10-553 | 65.52614345 | 0.25220288 | 0.00249997 | 0.128129261 | 1.87304 | 0.21734 |
| FxJj11-74 | 70.05084487 | 0.198085484 | 0.002071871 | 0.100990568 | 1.76981 | 0.13569 |
| FxJj11-74 | 69.6401737 | 0.017171716 | 6.38787E-05 | 0.007493082 | 0.4886 | 0.01522 |
| FxJj11-74 | 69.866033 | 3.179121822 | 0.090236674 | 1.402685313 | 9.74335 | 2.19112 |
| FxJj11-74 | 71.00672087 | 0.116920819 | 0.001122671 | 0.058033702 | 1.24482 | 0.09971 |
| FxJj11-74 | 71.87786786 | 0.268737734 | 0.003082049 | 0.126967896 | 1.93894 | 0.24186 |
| FxJj11-74 | 71.75257505 | 0.039189255 | 0.000243108 | 0.022478589 | 0.79424 | 0.03737 |
| FxJj11-74 | 72.13975734 | 1.272778485 | 0.019152384 | 0.574244722 | 5.70636 | 0.93467 |
| FxJj11-74 | 71.75371852 | 0.087575118 | 0.000317925 | 0.039359631 | 1.01124 | 0.06277 |
| FxJj11-74 | 72.27490707 | 0.328396258 | 0.003284679 | 0.187026263 | 2.55478 | 0.23814 |
| FxJj11-74 | 68.56784575 | 0.600730789 | 0.007768305 | 0.302508367 | 2.49657 | 0.40944 |
| FxJj11-74 | 72.28244969 | 3.408821956 | 0.029045917 | 1.920174222 | 9.84094 | 3.04123 |
| FxJj11-74 | 72.31312331 | 1.001586796 | 0.009026227 | 0.527914398 | 4.45148 | 0.82758 |
| FxJj11-74 | 72.40321658 | 0.114767319 | 0.000787218 | 0.06413106 | 1.40748 | 0.10109 |
| FxJj11-74 | 72.4366478 | 0.908746339 | 0.010881865 | 0.577968681 | 4.19731 | 0.83544 |
| FxJj11-74 | 71.006042 | 0.088172716 | 0.000889191 | 0.041485802 | 1.04636 | 0.07063 |
| FxJj11-74 | 72.29345593 | 0.238207842 | 0.001040493 | 0.119695295 | 1.86723 | 0.18278 |
| FxJj11-74 | 72.50198296 | 0.499402477 | 0.002551878 | 0.235303452 | 2.81841 | 0.4625 |
| FxJj11-74 | 72.03290585 | 0.267403247 | 0.002287379 | 0.145468818 | 1.50263 | 0.14955 |
| FxJj11-74 | 72.56014951 | 2.682074689 | 0.027516102 | 1.353740302 | 6.64991 | 2.18677 |
| FxJj11-74 | 71.2719202 | 0.776583022 | 0.007365767 | 0.391149663 | 3.98783 | 0.67159 |
| FxJj11-74 | 72.12646112 | 0.199535275 | 0.001204829 | 0.100699574 | 1.6525 | 0.18279 |
| FxJj11-74 | 72.6323272 | 4.901760474 | 0.059113775 | 2.543556022 | 12.59962 | 4.19373 |
| FxJj11-74 | 72.51680056 | 0.135335352 | 0.000863462 | 0.068617084 | 1.30372 | 0.12177 |
| FxJj11-74 | 72.78872297 | 0.139411221 | 0.00093537 | 0.068006956 | 1.47016 | 0.13294 |
| FxJj11-74 | 71.02309146 | 0.164813394 | 0.001306667 | 0.088182169 | 1.24482 | 0.0997 |
| FxJj11-74 | 70.86754759 | 0.573924636 | 0.004256239 | 0.240826827 | 2.44334 | 0.33095 |
| FxJj11-74 | 70.11445836 | 7.718628063 | 0.191648044 | 4.666328677 | 19.23442 | 5.45382 |
| FxJj11-74 | 69.8657617 | 0.389865622 | 0.004115829 | 0.201631124 | 2.56396 | 0.33232 |
| FxJj11-74 | 72.84355856 | 6.264420485 | 0.155957901 | 4.315926788 | 12.02134 | 5.13962 |
| FxJj11-74 | 71.97358891 | 2.266027737 | 0.043040097 | 1.254939281 | 7.36119 | 1.62518 |
| FxJj11-74 | 71.99118744 | 0.049845072 | 0.000492458 | 0.025371987 | 0.93717 | 0.0443 |
| FxJj11-74 | 70.40068827 | 0.383829769 | 0.003409075 | 0.193746873 | 2.45778 | 0.31156 |
| FxJj11-74 | 70.50393123 | 3.008228848 | 0.163532713 | 1.940018722 | 12.24514 | 1.97175 |
| FxJj11-74 | 72.45188305 | 2.238222017 | 0.033233195 | 1.335989337 | 6.76447 | 2.05527 |
| FxJj11-74 | 70.07895729 | 0.331317 | 0.003928457 | 0.162156049 | 2.13905 | 0.26032 |
| FxJj11-74 | 72.19014914 | 1.913360336 | 0.068592463 | 0.945414056 | 6.86628 | 1.57902 |
| FxJj11-74 | 72.17413967 | 0.061880557 | 0.000192448 | 0.028608112 | 0.9233 | 0.04986 |
| FxJj11-74 | 72.30763506 | 0.106293234 | 0.000705822 | 0.051987254 | 1.13803 | 0.08308 |
| FxJj11-74 | 69.99329802 | 3.244349421 | 0.075223231 | 1.732744031 | 9.86227 | 2.0008 |
| FxJj11-74 | 72.20536484 | 4.932397543 | 0.104752818 | 2.357118965 | 17.645 | 4.16133 |
| FxJj11-74 | 72.43480537 | 12.95041033 | 0.216633649 | 7.315334827 | 20.57044 | 10.28191 |
| FxJj11-74 | 70.56391306 | 9.184708718 | 0.18917603 | 4.555242447 | 19.42831 | 6.55157 |
| FxJj16-400 | 72.70927109 | 0.628503549 | 0.005281022 | 0.318952211 | 3.15727 | 0.45785 |
| FxJj16-400 | 74.1885711 | 1.054762265 | 0.010351647 | 0.549246621 | 3.58994 | 0.70219 |
| FxJj16-400 | 75.36649623 | 0.01318374 | 1.40368E-05 | 0.005734836 | 0.53757 | 0.0124 |
| FxJj16-400 | 75.32755939 | 2.442824747 | 0.027139514 | 1.405677972 | 7.06229 | 2.0243 |
| FxJj16-400 | 75.630859 | 3.322066232 | 0.068569648 | 1.776518937 | 9.29674 | 2.85884 |
| FxJj16-400 | 75.23692908 | 0.04018245 | 0.00011128 | 0.020888437 | 0.87675 | 0.03441 |
| FxJj16-400 | 75.38583166 | 0.019797183 | 5.17055E-05 | 0.009442839 | 0.54184 | 0.01377 |
| FxJj16-400 | 75.38161415 | 0.09024753 | 0.000383243 | 0.047304486 | 1.07517 | 0.07438 |
| FxJj16-400 | 75.42480691 | 1.616088985 | 0.020702482 | 0.939922199 | 5.70905 | 1.36762 |
| FxJj16-400 | 73.92153929 | 2.630375682 | 0.0275279 | 1.531243618 | 8.29533 | 2.06505 |
| FxJj16-400 | 75.62503883 | 0.399409565 | 0.003237451 | 0.231401237 | 2.18725 | 0.32047 |
| FxJj16-400 | 75.03774895 | 2.089820913 | 0.030625481 | 1.199237267 | 7.58179 | 1.83267 |
| FxJj16-400 | 75.47543186 | 9.383177204 | 0.632764213 | 6.012586676 | 26.70011 | 7.67388 |
| FxJj16-400 | 74.81352167 | 3.457312903 | 0.045262285 | 1.764327521 | 7.91583 | 2.92761 |
| FxJj16-400 | 74.62218587 | 0.077467696 | 0.000531429 | 0.039365511 | 0.98293 | 0.06198 |
| FxJj16-400 | 74.58480884 | 0.072211609 | 0.000381119 | 0.033605975 | 0.98259 | 0.06194 |
| FxJj16-408 | 74.21058938 | 0.871868005 | 0.015986533 | 0.431053517 | 3.9117 | 0.68499 |
| FxJj16-408 | 75.1020398 | 0.696240361 | 0.003220957 | 0.350981872 | 2.61109 | 0.41591 |
| FxJj16-408 | 75.00090003 | 0.62587758 | 0.006296467 | 0.327374799 | 2.69974 | 0.46944 |
| FxJj16-408 | 73.90453536 | 0.123043296 | 0.000790017 | 0.055457242 | 1.17623 | 0.08668 |
| FxJj16-408 | 75.32996226 | 18.32014126 | 0.306952051 | 9.078178322 | 30.61709 | 14.00492 |
| FxJj16-408 | 70.81001079 | 3.306619329 | 0.130560659 | 1.586901143 | 7.70791 | 2.0006 |
| FxJj16-408 | 71.24383174 | 0.581823377 | 0.014893254 | 0.281587278 | 2.34314 | 0.37556 |
| FxJj16-408 | 75.14215956 | 1.900025457 | 0.049463848 | 1.18175448 | 6.84052 | 1.61242 |
| FxJj16-408 | 75.13815556 | 1.337055583 | 0.036340449 | 0.751502178 | 5.36358 | 1.12191 |
| FxJj16-408 | 74.82280791 | 0.187807617 | 0.001302042 | 0.087300226 | 1.61116 | 0.17877 |
| FxJj16-453 | 73.64659003 | 3.030025252 | 0.048388689 | 1.834339749 | 8.13395 | 2.65546 |
| FxJj16-453 | 72.42442462 | 2.784083933 | 0.109887649 | 1.553420272 | 8.96072 | 2.19524 |
| FxJj16-453 | 72.72422229 | 0.117004476 | 0.001105598 | 0.057555238 | 1.23315 | 0.08886 |
| FxJj16-453 | 73.20463273 | 3.767394228 | 0.089235167 | 2.18473438 | 12.30842 | 2.99383 |
| FxJj16-453 | 74.44819631 | 15.46526115 | 0.325676017 | 7.65873568 | 32.0329 | 12.30988 |
| FxJj16-453 | 72.92279591 | 0.156902192 | 0.000519309 | 0.089886606 | 1.55319 | 0.15073 |
| FxJj16-453 | 74.15827182 | 0.38328127 | 0.003897528 | 0.175394659 | 2.38276 | 0.34643 |
| FxJj16-453 | 72.78361171 | 0.450388329 | 0.007661563 | 0.210851909 | 2.45128 | 0.29988 |
| FxJj16-453 | 75.1181766 | 0.433081216 | 0.008090315 | 0.21307936 | 3.05798 | 0.39506 |
| FxJj16-453 | 75.07103624 | 1.404927679 | 0.02177758 | 0.781144951 | 4.5655 | 1.24756 |
| FxJj16-453 | 75.47889542 | 1.778608579 | 0.056073464 | 0.927954585 | 6.44264 | 1.42682 |
| FxJj16-453 | 76.20284173 | 0.112899571 | 0.001042425 | 0.060515957 | 1.21811 | 0.09519 |
| FxJj16-453 | 76.39877293 | 0.108986509 | 0.000408867 | 0.052482703 | 1.36315 | 0.10578 |
| FxJj16-453 | 76.63660659 | 0.054709803 | 9.23606E-05 | 0.02483507 | 0.92478 | 0.05234 |
| FxJj16-453 | 73.46114147 | 4.115652645 | 0.793685896 | 2.167787733 | 12.81805 | 3.42271 |
| FxJj16-453 | 76.60842333 | 4.033467544 | 0.054177387 | 2.10060047 | 10.51652 | 3.67868 |
| FxJj16-453 | 77.58831114 | 0.272099712 | 0.001910201 | 0.13614499 | 2.1279 | 0.26016 |
| FxJj16-453 | 77.665784 | 1.223205092 | 0.01821079 | 0.676319557 | 4.76999 | 1.16297 |
| FxJj16-453 | 77.25865443 | 3.039141823 | 0.097110122 | 1.748271439 | 9.76228 | 2.74188 |
| FxJj16-453 | 77.19954839 | 0.041874125 | 9.80256E-05 | 0.018733585 | 0.78418 | 0.03596 |
| FxJj16-453 | 77.9463446 | 0.111723026 | 0.000804772 | 0.062202931 | 1.30925 | 0.11 |
| FxJj16-453 | 77.23203351 | 2.888532512 | 0.104590399 | 1.772822947 | 8.44541 | 2.17519 |
| FxJj16-453 | 77.94420162 | 0.042354166 | 0.000158953 | 0.02048383 | 0.76938 | 0.03331 |
| FxJj16-453 | 77.88053056 | 9.799604892 | 0.253396475 | 5.012823008 | 17.33934 | 8.20154 |
| FxJj16-549 | 67.55371878 | 0.572056056 | 0.008021171 | 0.289022957 | 3.28439 | 0.55235 |
| FxJj16-549 | 73.02300868 | 7.851399364 | 0.420521411 | 5.184710686 | 15.06774 | 7.4696 |
| FxJj16-549 | 71.06857183 | 1.379618183 | 0.03098082 | 0.660340006 | 6.06583 | 1.31825 |
| FxJj16-549 | 71.26307527 | 9.17215626 | 0.356682049 | 5.761362352 | 21.38303 | 8.88293 |
| FxJj16-549 | 70.38874939 | 3.933575633 | 0.188451333 | 2.108701966 | 14.59152 | 3.78859 |
| FxJj16-549 | 69.84268537 | 0.655310959 | 0.011799904 | 0.270696175 | 3.34703 | 0.6276 |
| FxJj16-549 | 69.91143127 | 0.146182663 | 0.001696618 | 0.074196975 | 1.58332 | 0.13978 |
| FxJj16-549 | 69.95473773 | 0.07176942 | 0.000383142 | 0.035469302 | 1.06123 | 0.07039 |
| FxJj16-549 | 70.1215242 | 1.561060341 | 0.029861805 | 0.767638319 | 5.70382 | 1.52463 |
| FxJj16-549 | 69.73597515 | 0.088539439 | 0.000280071 | 0.037520973 | 1.17112 | 0.08798 |
| FxJj16-549 | 69.57910049 | 0.111069837 | 0.001066719 | 0.050335441 | 1.34263 | 0.10559 |
| FxJj16-549 | 70.04388888 | 2.393854765 | 0.054605301 | 1.316650198 | 7.58199 | 2.32411 |
| FxJj16-549 | 70.08536799 | 5.021180755 | 0.235741579 | 2.358011672 | 14.32862 | 4.71582 |
| FxJj16-549 | 70.11091276 | 1.781068257 | 0.036158364 | 0.908279373 | 6.16649 | 1.73224 |
| FxJj16-549 | 69.84227119 | 2.250189501 | 0.219911517 | 1.584572812 | 9.46943 | 2.09098 |
| FxJj16-552 | 77.3503406 | 1.045735034 | 0.023121344 | 0.623520126 | 4.51889 | 0.69386 |
| FxJj16-552 | 77.50618189 | 0.083012231 | 0.000327443 | 0.042347313 | 1.07654 | 0.07435 |
| FxJj16-552 | 77.52975364 | 0.061762837 | 0.000152501 | 0.03164439 | 0.97552 | 0.05948 |
| FxJj16-552 | 77.59324083 | 0.120042552 | 0.001208543 | 0.053776777 | 1.27858 | 0.10409 |
| FxJj16-552 | 77.75487172 | 0.431919896 | 0.004143689 | 0.201786249 | 2.19364 | 0.32709 |
| FxJj16-552 | 77.53565388 | 0.062788856 | 0.000216213 | 0.023885072 | 1.03691 | 0.04492 |
| FxJj16-552 | 77.32331232 | 15.4194175 | 0.552465963 | 9.074000069 | 32.23092 | 12.23864 |
| FxJj16-552 | 77.58329937 | 5.387186168 | 0.079474946 | 3.201939891 | 10.29249 | 3.58103 |
| FxJj16-552 | 77.9304625 | 0.489326683 | 0.004401485 | 0.237253371 | 3.324 | 0.4262 |
| FxJj16-552 | 76.93975308 | 0.505079305 | 0.001301305 | 0.227672703 | 3.0568 | 0.4311 |
| FxJj16-552 | 77.82326192 | 0.541744251 | 0.006164476 | 0.298438624 | 3.04259 | 0.43243 |
| FxJj16-552 | 77.20544804 | 1.106251911 | 0.018767287 | 0.575998148 | 4.2212 | 0.80291 |
| FxJj16-552 | 76.88119896 | 0.984403076 | 0.019461496 | 0.580621037 | 3.74259 | 0.86405 |
| FxJj16-552 | 77.35953987 | 0.130241033 | 0.000592342 | 0.070712596 | 1.31274 | 0.10034 |
| FxJj16-552 | 77.18179796 | 0.157501579 | 0.0013481 | 0.091310554 | 0.97552 | 0.05948 |
| FxJj16-552 | 78.18218115 | 6.201113522 | 0.083175187 | 3.201636989 | 11.70109 | 5.27955 |
| FxJj16-552 | 77.92751524 | 0.726097709 | 0.008430811 | 0.383676891 | 3.9943 | 0.59473 |
| FxJj16-552 | 77.75669595 | 0.062589276 | 0.000191321 | 0.0247256 | 0.87337 | 0.04461 |
| FxJj16-552 | 77.53951487 | 0.479312638 | 0.006937526 | 0.254231863 | 2.92638 | 0.41138 |
| FxJj16-552 | 77.8215546 | 1.534235206 | 0.042490324 | 0.727553409 | 6.49867 | 1.17501 |
| FxJj16-552 | 77.66130107 | 0.135011669 | 0.000639137 | 0.074706925 | 1.33356 | 0.114 |
| FxJj16-552 | 77.67702628 | 1.56851814 | 0.019876422 | 0.948978346 | 5.24979 | 1.27953 |
| FxJj16-552 | 77.39501778 | 4.076691778 | 0.136097247 | 2.394056329 | 9.77574 | 3.16855 |
| FxJj16-552 | 77.25731954 | 0.111769106 | 0.000401542 | 0.052833269 | 1.19295 | 0.09049 |
| FxJj16-552 | 77.35244398 | 2.412227234 | 0.030706854 | 1.266720555 | 5.93915 | 2.13015 |
| FxJj16-552 | 77.21480326 | 5.18480874 | 0.067624751 | 2.765486089 | 12.00074 | 3.80618 |
| FxJj16-552 | 75.79693326 | 0.441510545 | 0.011008083 | 0.222876337 | 2.67635 | 0.38291 |
| FxJj16-552 | 75.30694631 | 0.212104996 | 0.003293508 | 0.107679807 | 1.86716 | 0.17842 |
| FxJj16-580 | 78.07773268 | 17.244262 | 1.149813178 | 9.446035693 | 28.24481 | 13.99152 |
| FxJj16-580 | 77.93915237 | 0.039479817 | 4.48372E-05 | 0.0172509 | 0.79738 | 0.0384 |
| FxJj16-580 | 78.26181196 | 20.80069744 | 2.355968858 | 9.926629103 | 40.01787 | 15.68826 |
| FxJj16-580 | 75.83176752 | 0.122261445 | 0.001001603 | 0.059855479 | 1.2694 | 0.10369 |
| FxJj16-580 | 74.59408411 | 1.446427777 | 0.021077836 | 0.786304695 | 4.78892 | 1.19673 |
| FxJj16-580 | 74.94136593 | 1.46188588 | 0.0219579 | 0.840150968 | 4.88296 | 1.12758 |
| FxJj16-580 | 70.48146505 | 1.584540829 | 0.11985145 | 0.856015761 | 4.7061 | 0.93324 |
| FxJj16-580 | 73.94968239 | 4.324069382 | 0.108814355 | 2.477080958 | 10.55811 | 3.52137 |
| FxJj16-580 | 73.33345009 | 4.296875231 | 0.118351911 | 2.135811273 | 9.41083 | 2.90324 |
| FxJj16-580 | 69.92234967 | 1.42454259 | 0.069942825 | 0.765054375 | 5.38563 | 0.89853 |
| FxJj16-580 | 70.35267428 | 0.519418058 | 0.013383794 | 0.265483601 | 2.38498 | 0.31108 |
| FxJj16-580 | 73.24709127 | 2.762238494 | 0.194922495 | 1.813399297 | 8.4117 | 2.17309 |
| FxJj16-587 | 67.69792205 | 0.175592818 | 0.00260018 | 0.080895749 | 1.76895 | 0.15824 |
| FxJj16-587 | 67.6731243 | 0.454817696 | 0.009136469 | 0.220402048 | 1.91966 | 0.24374 |
| FxJj16-587 | 67.8836354 | 0.135599935 | 0.001086059 | 0.074063323 | 1.29537 | 0.09231 |
| FxJj16-587 | 67.96319984 | 0.354527159 | 0.00225419 | 0.200720105 | 2.08798 | 0.27015 |
| FxJj16-587 | 69.61960171 | 0.13879176 | 0.001256957 | 0.065669316 | 1.19439 | 0.08064 |
| FxJj16-587 | 69.68311718 | 0.420746628 | 0.002024072 | 0.197098969 | 2.43583 | 0.32036 |
| FxJj16-587 | 69.51353449 | 18.12191958 | 1.266451971 | 6.743927313 | 30.86245 | 11.59533 |
| FxJj16-587 | 71.40276065 | 0.429844828 | 0.004787106 | 0.219853999 | 2.73423 | 0.40858 |
| FxJj16-587 | 71.4308869 | 0.372774705 | 0.006313586 | 0.179785345 | 2.45277 | 0.32256 |
| FxJj16-587 | 71.33305034 | 0.102982672 | 0.001216687 | 0.049559693 | 1.12118 | 0.08064 |
| FxJj16-587 | 71.64463574 | 0.477007551 | 0.00815827 | 0.226670993 | 2.81197 | 0.45159 |
| FxJj16-587 | 71.15494036 | 1.292810089 | 0.057260732 | 0.622730444 | 4.99239 | 0.68413 |
| FxJj16-587 | 73.30988538 | 0.184092369 | 0.005775603 | 0.096510862 | 1.48055 | 0.14518 |
| FxJj16-587 | 73.80038174 | 0.327361485 | 0.006245728 | 0.149569421 | 1.58576 | 0.16133 |
| FxJj16-587 | 75.52023932 | 1.001354482 | 0.019477848 | 0.514332329 | 4.55347 | 0.82934 |
| FxJj16-587 | 74.55889301 | 2.135532444 | 0.105769322 | 1.161554579 | 6.75216 | 1.72316 |
| FxJj16-587 | 76.94184876 | 4.754656908 | 0.175613504 | 2.63796269 | 13.10843 | 4.57625 |
| FxJj16-587 | 75.98161433 | 2.90631199 | 0.105565238 | 1.800246148 | 7.79635 | 2.55695 |
| FxJj16-587 | 76.31094704 | 1.34780693 | 0.077302673 | 0.56583351 | 5.56494 | 1.20697 |
| FxJj16-587 | 77.49594643 | 0.654116094 | 0.025870123 | 0.355704245 | 3.63131 | 0.58467 |
| FxJj16-587 | 77.92034739 | 2.845588674 | 0.109459132 | 1.531838101 | 6.72124 | 2.33994 |
| FxJj16-587 | 75.40378151 | 0.670637588 | 0.014692156 | 0.352541454 | 3.72219 | 0.6087 |
| FxJj16-587 | 77.18910159 | 3.887281284 | 0.101774374 | 2.158431824 | 11.87573 | 3.76164 |
| FxJj16-587 | 76.44888393 | 13.54160586 | 1.159198717 | 8.2216672 | 28.46649 | 11.18282 |
| FxJj16-587 | 75.06179737 | 0.658557497 | 0.014452304 | 0.297978447 | 3.6568 | 0.56813 |
| FxJj16-587 | 74.98469998 | 0.422932754 | 0.003618737 | 0.180184918 | 2.78009 | 0.35484 |
| FxJj16-680 | 71.64322122 | 0.250250138 | 0.005200705 | 0.114762196 | 1.80704 | 0.19638 |
| FxJj16-680 | 72.28613378 | 15.45239033 | 0.946974113 | 7.82241618 | 29.54207 | 12.11502 |
| FxJj16-680 | 71.66960114 | 8.654397061 | 0.415949023 | 4.590423621 | 14.98525 | 6.87108 |
| FxJj16-680 | 71.12233452 | 0.259437472 | 0.00483869 | 0.120476051 | 2.19818 | 0.24425 |
| FxJj16-680 | 70.97492576 | 3.283390743 | 0.045348377 | 1.786006301 | 9.30888 | 2.51073 |
| FxJj16-680 | 71.1508762 | 10.28068749 | 0.380273637 | 3.417562207 | 23.1605 | 8.41097 |
| FxJj16-680 | 71.12113784 | 1.050342006 | 0.007773558 | 0.566580949 | 4.38874 | 1.01571 |
| FxJj16-680 | 70.73926923 | 0.47841266 | 0.00464389 | 0.231707602 | 2.13969 | 0.32729 |
| FxJj16-680 | 71.03112883 | 1.209146952 | 0.031664638 | 0.717025862 | 5.63037 | 1.02721 |
| FxJj16-680 | 71.03924578 | 0.309335331 | 0.002536039 | 0.201793159 | 2.35007 | 0.3054 |
| FxJj16-680 | 70.51970662 | 0.297590828 | 0.004400675 | 0.14351083 | 2.01732 | 0.26184 |
| FxJj16-680 | 71.28308931 | 8.549803635 | 0.176498398 | 4.507811229 | 15.88784 | 7.56869 |
| FxJj16-680 | 70.63026068 | 1.655955488 | 0.014095878 | 0.895168515 | 5.07244 | 1.269 |
| FxJj16-680 | 69.50308617 | 0.399130004 | 0.003324977 | 0.24470669 | 2.91886 | 0.3551 |
| FxJj16-680 | 70.18890563 | 0.14867078 | 0.000942753 | 0.078917772 | 1.56475 | 0.14548 |
| FxJj16-680 | 69.67509412 | 0.08034843 | 0.000560389 | 0.040839734 | 1.08858 | 0.06787 |
| FxJj16-680 | 71.21095911 | 0.068558972 | 0.000610792 | 0.034395038 | 1.05788 | 0.06544 |
| FxJj16-680 | 69.81404862 | 0.097767143 | 0.000608402 | 0.051047023 | 1.18158 | 0.08726 |
| FxJj16-680 | 70.96413229 | 5.645617516 | 0.088816417 | 3.307751239 | 13.41216 | 5.43781 |
| FxJj16-680 | 71.40858795 | 3.283299851 | 0.057020619 | 2.037998158 | 8.96827 | 3.06342 |
| FxJj16-680 | 69.58462467 | 1.759237981 | 0.044704436 | 0.994953677 | 5.39167 | 1.36214 |
| FxJj16-680 | 70.80809095 | 2.605750115 | 0.041425508 | 1.156569743 | 8.33793 | 2.40335 |
| FxJj16-680 | 71.20664071 | 0.237812243 | 0.001046336 | 0.121036212 | 2.12935 | 0.23151 |
| FxJj16-680 | 71.33254445 | 0.451943599 | 0.002102727 | 0.271986402 | 2.4953 | 0.4351 |
| FxJj16-680 | 71.19177359 | 1.10801388 | 0.010966022 | 0.617151533 | 4.25098 | 0.98964 |
| FxJj16-680 | 71.34016087 | 9.167551901 | 0.151239264 | 4.763995889 | 17.81259 | 6.62314 |
| FxJj16-680 | 71.44881428 | 3.059828516 | 0.048205709 | 1.705060141 | 7.90517 | 2.88289 |
| FxJj16-680 | 71.90109412 | 1.469575799 | 0.019624628 | 0.822679276 | 4.70425 | 1.07017 |
| FxJj16-680 | 72.84911008 | 0.561747101 | 0.005590097 | 0.273795851 | 3.4902 | 0.54598 |
| FxJj16-680 | 72.86320646 | 4.800762006 | 0.207252981 | 2.719483324 | 13.24786 | 4.36205 |
| FxJj16-680 | 72.78348371 | 0.11930899 | 0.000313344 | 0.065229838 | 1.30409 | 0.10911 |
| FxJj16-680 | 72.61551017 | 10.15767562 | 0.460753447 | 5.066863199 | 21.63837 | 8.80111 |
| FxJj18GS-13-26 | 71.90888825 | 2.788653494 | 0.061149495 | 1.112283169 | 8.74174 | 1.96938 |
| FxJj18GS-13-26 | 71.78390463 | 0.055736661 | 0.000168393 | 0.027278456 | 0.92045 | 0.05435 |
| FxJj18GS-13-26 | 71.33192355 | 0.226890476 | 0.003204415 | 0.090146331 | 1.72804 | 0.19563 |
| FxJj18GS-13-26 | 72.64110277 | 1.884140308 | 0.033497003 | 0.980566926 | 7.29748 | 1.44354 |
| FxJj18GS-13-26 | 71.08821311 | 1.713635411 | 0.023325494 | 0.906101944 | 6.14006 | 1.47042 |
| FxJj18GS-13-26 | 71.9913133 | 0.17954801 | 0.001271025 | 0.088211094 | 1.76373 | 0.12589 |
| FxJj18GS-13-26 | 72.14168483 | 0.082079962 | 0.000495751 | 0.039914813 | 0.98044 | 0.05434 |
| FxJj18GS-13-26 | 72.19212842 | 0.279498385 | 0.002081475 | 0.154845321 | 1.96832 | 0.21373 |
| FxJj18GS-13-26 | 69.7752435 | 0.077654464 | 0.000486036 | 0.038670782 | 0.84615 | 0.04619 |
| FxJj18GS-13-26 | 72.8296228 | 1.815806522 | 0.01547021 | 0.894258196 | 4.34923 | 1.16279 |
| FxJj18GS-13-26 | 71.38494643 | 0.156142396 | 0.000580014 | 0.072663262 | 1.3543 | 0.12228 |
| FxJj18GS-13-26 | 72.33820742 | 0.018886949 | 6.59258E-05 | 0.009314448 | 0.51071 | 0.01722 |
| FxJj18GS-13-26 | 72.33812525 | 1.664932657 | 0.022129944 | 1.14674713 | 5.77336 | 1.25673 |
| FxJj18GS-13-26 | 72.46904214 | 0.286621384 | 0.002023003 | 0.173005148 | 1.92333 | 0.22944 |
| FxJj18GS-13-26 | 71.42314541 | 2.95779569 | 0.038816863 | 1.58703164 | 7.93837 | 2.47417 |
| FxJj18GS-13-26 | 72.33253566 | 0.488663545 | 0.003150281 | 0.263618266 | 2.56444 | 0.3224 |
| FxJj18GS-13-26 | 71.98233297 | 1.216539779 | 0.024894265 | 0.550134156 | 4.4399 | 0.79723 |
| FxJj18GS-13-26 | 72.20852416 | 1.63047261 | 0.018230529 | 0.890049533 | 4.92056 | 1.22563 |
| FxJj18GS-13-26 | 72.07656203 | 1.915119554 | 0.02360055 | 1.032259887 | 5.2209 | 1.10123 |
| FxJj18GS-13-26 | 71.00521629 | 0.248273387 | 0.002502915 | 0.133122797 | 1.71839 | 0.17385 |
| FxJj18GS-13-26 | 71.18876273 | 0.074084196 | 0.000393949 | 0.040289306 | 1.00668 | 0.0652 |
| FxJj18GS-13-26 | 72.00869877 | 0.048168709 | 0.000254186 | 0.023691376 | 0.83396 | 0.04347 |
| FxJj18GS-13-26 | 70.95352697 | 0.371051543 | 0.004653155 | 0.185101117 | 2.09974 | 0.27168 |
| FxJj18GS-13-26 | 71.39413382 | 0.80906988 | 0.009128802 | 0.450313508 | 2.95979 | 0.48453 |
| FxJj18GS-13-26 | 72.74910811 | 2.526699848 | 0.034964294 | 1.407618499 | 6.64458 | 1.63677 |
| FxJj18GS-13-26 | 71.81761238 | 1.864247382 | 0.037470082 | 0.970002563 | 6.82044 | 1.33915 |
| FxJj18GS-13-26 | 72.14709562 | 0.028886292 | 7.85411E-05 | 0.018402267 | 0.62462 | 0.02292 |
| FxJj18GS-13-26 | 71.09436201 | 0.197189215 | 0.002269607 | 0.098191133 | 1.64347 | 0.15941 |
| FxJj18GS-13-26 | 71.47926829 | 1.559955914 | 0.039691778 | 0.792950734 | 5.83235 | 1.02692 |
| FxJj18GS-13-26 | 72.15815283 | 0.74016093 | 0.006391293 | 0.33080383 | 3.87432 | 0.51224 |
| FxJj18GS-13-26 | 72.3614084 | 2.089214426 | 0.029438867 | 1.116839825 | 5.8707 | 1.50304 |
| FxJj18GS-13-26 | 71.7941052 | 0.953397942 | 0.011870565 | 0.515572592 | 2.92333 | 0.5373 |
| FxJj18GS-13-26 | 72.13283798 | 0.065958797 | 0.000262587 | 0.033278371 | 0.81712 | 0.04468 |
| FxJj18GS-13-26 | 72.39666838 | 2.099574317 | 0.028904656 | 1.069525972 | 4.79246 | 1.228 |
| FxJj18GS-13-26 | 72.19886831 | 0.206969651 | 0.000808394 | 0.096244818 | 1.44866 | 0.12165 |
| FxJj18GS-13-26 | 69.6665989 | 0.182132753 | 0.003447857 | 0.086810371 | 1.42342 | 0.13036 |
| FxJj18GS-13-26 | 72.1966108 | 3.337701301 | 0.059351091 | 1.637313554 | 7.01087 | 1.89124 |
| FxJj18GS-13-26 | 72.35471805 | 1.151205739 | 0.019388081 | 0.580480835 | 3.96786 | 0.94845 |
| FxJj18GS-13-26 | 71.89642991 | 2.231819202 | 0.042609465 | 1.260691167 | 5.98075 | 1.57755 |
| FxJj18GS-13-26 | 71.34267707 | 0.061478006 | 0.000530721 | 0.030270788 | 0.83396 | 0.04347 |
| FxJj18GS-13-26 | 71.45462631 | 0.137968802 | 0.002177372 | 0.068999206 | 1.30168 | 0.1087 |
| FxJj18GS-13-26 | 71.24052835 | 0.926930894 | 0.024343795 | 0.514853328 | 3.43709 | 0.60858 |
| FxJj18GS-2148 | 71.87583656 | 1.47488554 | 0.031144253 | 0.74245212 | 6.44742 | 1.33995 |
| FxJj18GS-2148 | 73.99354294 | 0.551705023 | 0.008647798 | 0.277744032 | 3.26649 | 0.47993 |
| FxJj18GS-2148 | 74.08064603 | 0.111517834 | 0.001311989 | 0.057140954 | 1.26534 | 0.10026 |
| FxJj18GS-2148 | 74.16726205 | 0.759085075 | 0.017499613 | 0.433092302 | 3.9206 | 0.74401 |
| FxJj18GS-2148 | 74.30488482 | 0.044081345 | 0.000179931 | 0.021386217 | 0.84362 | 0.04368 |
| FxJj18GS-2148 | 74.37799139 | 0.059629478 | 0.000294579 | 0.030874478 | 0.94846 | 0.05783 |
| FxJj18GS-2148 | 74.46074824 | 1.915128481 | 0.016808159 | 0.922955166 | 5.39049 | 1.67276 |
| FxJj18GS-2148 | 74.1796254 | 0.229565881 | 0.001409758 | 0.133060186 | 1.91046 | 0.22746 |
| FxJj18GS-2148 | 74.24366111 | 0.511995233 | 0.006210539 | 0.201411235 | 3.07481 | 0.50591 |
| FxJj18GS-2148 | 74.29018409 | 0.622482881 | 0.004830191 | 0.289400963 | 3.43145 | 0.55903 |
| FxJj18GS-2148 | 73.94392619 | 0.6576498 | 0.01265646 | 0.384900776 | 3.62913 | 0.64378 |
| FxJj18GS-2148 | 74.59679795 | 0.488910467 | 0.008670629 | 0.253987453 | 2.84434 | 0.478 |
| FxJj18GS-2148 | 74.53832726 | 0.012187095 | 2.29948E-05 | 0.006976316 | 0.51913 | 0.01156 |
| FxJj18GS-2148 | 74.51700344 | 0.42369428 | 0.002436078 | 0.196997824 | 2.57812 | 0.39351 |
| FxJj18GS-2148 | 74.69066905 | 0.020263191 | 1.31145E-05 | 0.010835328 | 0.56368 | 0.02023 |
| FxJj18GS-2148 | 74.46450318 | 0.248921318 | 0.000383023 | 0.081804823 | 1.99167 | 0.24864 |
| FxJj18GS-2148 | 74.44003581 | 0.148534044 | 0.001611654 | 0.079145124 | 1.46678 | 0.14361 |
| FxJj18GS-2148 | 73.29204593 | 0.445158988 | 0.009400338 | 0.222605544 | 2.50704 | 0.34693 |
| FxJj18GS-2148 | 74.56548381 | 8.768911634 | 0.201257688 | 4.17781725 | 27.66568 | 8.12655 |
| FxJj18GS-2148 | 73.555216 | 1.170421415 | 0.020984104 | 0.688736849 | 4.64834 | 0.97726 |
| FxJj18GS-2148 | 74.30972126 | 3.048673738 | 0.119892849 | 1.906450749 | 10.62444 | 2.83171 |
| FxJj18GS-2148 | 73.67467013 | 0.224292435 | 0.00146374 | 0.110998299 | 1.98794 | 0.21974 |
| FxJj18GS-5305 | 51.42584585 | 0.137453319 | 0.001001692 | 0.072490244 | 0.96492 | 0.05819 |
| FxJj18GS-5305 | 52.15190826 | 3.600627391 | 0.097872668 | 1.257321843 | 9.65936 | 2.73146 |
| FxJj18GS-5305 | 52.75403581 | 0.500150512 | 0.003960073 | 0.284296184 | 2.32628 | 0.31317 |
| FxJj18GS-5305 | 52.98018718 | 2.015055891 | 0.098724686 | 0.908946854 | 8.17143 | 1.3219 |
| FxJj18GS-5305 | 53.20126902 | 0.063598259 | 0.000442245 | 0.032635422 | 0.97796 | 0.05334 |
| FxJj18GS-5305 | 56.22061503 | 0.198063252 | 0.001066065 | 0.100074517 | 1.39069 | 0.12 |
| FxJj18GS-5305 | 56.26436801 | 0.314717112 | 0.002195769 | 0.165693223 | 1.63212 | 0.19278 |
| FxJj18GS-5305 | 55.74837051 | 0.631542835 | 0.008941383 | 0.364184472 | 2.87398 | 0.41502 |
| FxJj18GS-5305 | 53.35186094 | 1.481407075 | 0.01292124 | 0.616819909 | 5.70283 | 1.22857 |
| FxJj18GS-5305 | 56.33886314 | 1.979459858 | 0.040558417 | 0.832230001 | 7.12588 | 1.39428 |
| FxJj18GS-5305 | 56.54603704 | 0.088198031 | 0.000628606 | 0.047600223 | 1.06465 | 0.07271 |
| FxJj18GS-5305 | 56.95667535 | 0.392504197 | 0.003762808 | 0.179111989 | 2.35529 | 0.31196 |
| FxJj18GS-5305 | 56.26360049 | 0.078263221 | 0.000222945 | 0.034860205 | 1.17683 | 0.07396 |
| FxJj18GS-5305 | 56.90089744 | 16.86090464 | 0.665278704 | 8.044032597 | 31.74851 | 12.06446 |
| FxJj18GS-5305 | 56.50532951 | 3.774180223 | 0.059621967 | 1.910935417 | 8.48939 | 2.85969 |
| FxJj18GS-5305 | 56.20043487 | 0.23060371 | 0.0026583 | 0.130919035 | 1.86001 | 0.19879 |
| FxJj18GS-5715 | 74.21580053 | 0.019337895 | 5.48119E-05 | 0.010511022 | 0.51569 | 0.01693 |
| FxJj18GS-5715 | 74.32589514 | 0.087751996 | 0.000214819 | 0.042493094 | 1.12191 | 0.07742 |
| FxJj18GS-5715 | 74.34909733 | 1.624283605 | 0.008816183 | 0.838742167 | 5.50802 | 1.01184 |
| FxJj18GS-5715 | 74.19331865 | 9.817378351 | 0.108799116 | 4.547079976 | 23.14969 | 8.11826 |
| FxJj18GS-5715 | 74.45544952 | 0.089197922 | 0.000400917 | 0.050553874 | 1.18665 | 0.07529 |
| FxJj18GS-5715 | 74.43892235 | 0.228735146 | 0.002179755 | 0.109154874 | 1.7947 | 0.17903 |
| FxJj18GS-5715 | 74.49604781 | 0.276248895 | 0.002134742 | 0.139722497 | 1.63685 | 0.17422 |
| FxJj18GS-5715 | 74.45999419 | 0.031729322 | 0.000136364 | 0.015182769 | 0.62234 | 0.01937 |
| FxJj18GS-5715 | 74.49252284 | 0.450405544 | 0.007228587 | 0.201933199 | 3.21903 | 0.37181 |
| FxJj18GS-5715 | 74.641384 | 0.066184687 | 0.000255577 | 0.031425909 | 0.86849 | 0.04839 |
| FxJj18GS-5715 | 74.83843348 | 0.073655427 | 0.000317752 | 0.0344784 | 0.90036 | 0.05806 |
| FxJj18GS-5715 | 74.72898036 | 4.363593455 | 0.054491437 | 1.987723572 | 13.99293 | 3.73187 |
| FxJj18GS-5715 | 74.67867092 | 0.238318252 | 0.001069227 | 0.137056028 | 1.9684 | 0.22904 |
| FxJj18GS-5715 | 74.8645066 | 2.442748869 | 0.030126889 | 1.082848105 | 6.51037 | 1.70917 |
| FxJj18GS-5715 | 71.38045692 | 0.186039184 | 0.001235877 | 0.086402373 | 1.75813 | 0.13387 |
| FxJj18GS-5715 | 71.48760349 | 0.14864601 | 0.00113649 | 0.067849905 | 1.42512 | 0.12584 |
| FxJj18GS-5715 | 71.54620355 | 2.068463614 | 0.020460974 | 0.90279842 | 7.73587 | 1.66456 |
| FxJj18GS-5715 | 71.98195997 | 0.957722853 | 0.014906837 | 0.449928845 | 5.20789 | 0.79035 |
| FxJj18GS-5715 | 71.10903337 | 0.092378734 | 0.000612658 | 0.044473631 | 1.07582 | 0.07419 |
| FxJj18GS-5715 | 71.05317942 | 0.115048456 | 0.000864737 | 0.052678473 | 1.35233 | 0.09757 |
| FxJj18GS-5715 | 70.74862355 | 0.069700762 | 0.000631304 | 0.038058662 | 0.99516 | 0.06048 |
| GaJi4-1363 | 22.65492616 | 1.987358786 | 0.025009943 | 1.181486094 | 3.75528 | 0.77825 |
| GaJi4-1363 | 21.18733541 | 0.653777788 | 0.005991676 | 0.328365121 | 2.22554 | 0.29252 |
| GaJi4-1363 | 20.66295939 | 0.066552306 | 0.000311211 | 0.030780027 | 0.76707 | 0.03747 |
| GaJi4-1363 | 25.32423243 | 0.542371114 | 0.005185225 | 0.276626601 | 2.1192 | 0.32108 |
| GaJi4-1363 | 25.40382949 | 0.261813396 | 0.001328404 | 0.112205826 | 1.5842 | 0.17122 |
| GaJi4-1363 | 26.49910368 | 0.084567707 | 0.000336909 | 0.042426571 | 0.75392 | 0.03477 |
| GaJi4-1363 | 26.49866614 | 0.375707906 | 0.001463781 | 0.191921065 | 1.11928 | 0.08562 |
| GaJi4-1363 | 26.52348317 | 0.146805408 | 0.000660768 | 0.077396353 | 1.48183 | 0.11031 |
| GaJi4-1363 | 26.72812297 | 1.318632934 | 0.01066203 | 0.608776389 | 3.77564 | 0.71263 |
| GaJi4-1363 | 26.66152768 | 0.196880954 | 0.001122886 | 0.110464556 | 1.72777 | 0.1641 |
| GaJi4-1363 | 26.68842808 | 0.164824988 | 0.000614621 | 0.087231697 | 1.48333 | 0.13438 |
| GaJi4-1363 | 26.72850955 | 0.768001262 | 0.004814546 | 0.360038741 | 3.482 | 0.4741 |
| GaJi4-1363 | 23.39569075 | 0.875779132 | 0.026812245 | 0.447546105 | 3.88435 | 0.62167 |
| GaJi4-1363 | 26.58146363 | 0.049952626 | 0.000208294 | 0.023938857 | 0.8276 | 0.04281 |
| GaJi4-1363 | 24.87959102 | 1.866576858 | 0.021606452 | 0.970926003 | 4.01023 | 0.85173 |
| GaJi4-1363 | 26.79492027 | 3.826445512 | 0.028571593 | 1.934642094 | 9.18503 | 2.72771 |
| GaJi4-1363 | 26.67607798 | 0.0352175 | 9.45851E-05 | 0.016228048 | 0.74098 | 0.03211 |
| GaJi4-1363 | 25.20316418 | 1.339935137 | 0.011844417 | 0.714103316 | 5.35775 | 0.8604 |
| GaJi4-1363 | 25.88926883 | 0.082304518 | 0.000583028 | 0.042380217 | 1.0847 | 0.07491 |
| GaJi4-1363 | 26.24844372 | 14.23714954 | 1.542174163 | 9.78465068 | 29.86797 | 9.31933 |
| GaJi4-1433 | -2.853254604 | 6.278282277 | 0.028550737 | 3.28692892 | 11.75555 | 4.48145 |
| GaJi4-1433 | -2.931108001 | 1.099517774 | 0.008108496 | 0.703059063 | 4.34863 | 0.82044 |
| GaJi4-1433 | -2.932528787 | 0.267831783 | 0.001342779 | 0.133936529 | 1.65072 | 0.18048 |
| GaJi4-1433 | -3.006240285 | 3.520559782 | 0.030729041 | 1.65258462 | 9.32394 | 2.54984 |
| GaJi4-1433 | -3.266214222 | 0.143907957 | 0.000639457 | 0.070909802 | 1.25012 | 0.10027 |
| GaJi4-1433 | -2.924011364 | 1.02915764 | 0.004475652 | 0.495284677 | 4.08886 | 0.81541 |
| GaJi4-1433 | -2.894744099 | 0.726962287 | 0.003420079 | 0.35746817 | 3.33698 | 0.44113 |
| GaJi4-1433 | -3.203967841 | 0.726435459 | 0.00618939 | 0.416379643 | 2.5359 | 0.4077 |
| GaJi4-3105 | 32.36765882 | 0.733850983 | 0.008844663 | 0.325846472 | 2.21983 | 0.33462 |
| GaJi4-3105 | 32.88759331 | 3.169053871 | 0.041363557 | 1.613054359 | 7.00119 | 2.04499 |
| GaJi4-3105 | 34.09657266 | 2.099447759 | 0.039098775 | 1.232895946 | 5.84276 | 1.42517 |
| GaJi4-3105 | 32.05172654 | 2.700119689 | 0.077148389 | 1.522732559 | 6.79966 | 1.59596 |
| GaJi4-3105 | 34.25907188 | 4.321988467 | 0.071858085 | 2.498554245 | 9.5684 | 2.94313 |
| GaJi4-3105 | 32.9296719 | 0.149877106 | 0.001313741 | 0.07506381 | 1.25352 | 0.1008 |
| GaJi4-3105 | 34.04983237 | 0.104597086 | 0.000657563 | 0.051548641 | 1.13193 | 0.0739 |
| GaJi4-3105 | 34.19834988 | 0.407442782 | 0.003311231 | 0.217378618 | 2.20264 | 0.27553 |
| GaJi4-3105 | 33.00900664 | 1.286060278 | 0.016550524 | 0.608466967 | 4.25921 | 0.93402 |
| GaJi4-3105 | 34.05715595 | 1.836975265 | 0.036308927 | 0.924858555 | 6.24018 | 1.36298 |
| GaJi4-3105 | 33.11585183 | 3.779689475 | 0.090056979 | 2.132432414 | 6.72648 | 1.83779 |
| GaJi4-3105 | 34.25254976 | 0.906091642 | 0.005768717 | 0.513480444 | 3.79624 | 0.69553 |
| GaJi4-3105 | 30.95232375 | 0.152514597 | 0.000435147 | 0.076725672 | 1.25352 | 0.1008 |
| GaJi4-3105 | 32.99482208 | 3.880648446 | 0.072682507 | 2.001681882 | 7.50493 | 2.00802 |
| GaJi4-3105 | 30.9938382 | 0.991947509 | 0.005942777 | 0.553197214 | 4.43307 | 0.58101 |
| GaJi4-3105 | 34.51499115 | 0.3188925 | 0.002751415 | 0.165250153 | 1.73559 | 0.19989 |
| GaJi4-3105 | 34.81855087 | 1.170826623 | 0.00924317 | 0.595648721 | 4.01218 | 0.77108 |
| GaJi4-3105 | 34.66929929 | 0.155938409 | 0.000571246 | 0.064754991 | 1.37098 | 0.12094 |
| GaJi4-3105 | 34.87608932 | 7.173141458 | 0.077872885 | 4.207029673 | 12.14874 | 4.92151 |
| GaJi4-3105 | 34.80930533 | 0.251028346 | 0.000721387 | 0.140942959 | 1.65512 | 0.18144 |
| GaJi4-3105 | 34.66438179 | 0.168339435 | 0.002401605 | 0.091849093 | 1.39375 | 0.11647 |
| GaJi4-3105 | 33.3747993 | 2.3006926 | 0.062292309 | 1.401547073 | 5.47637 | 1.19097 |
| GaJi4-3105 | 34.63201485 | 0.240001822 | 0.002905458 | 0.124655096 | 1.60946 | 0.16574 |
| GaJi4-3105 | 34.92010968 | 0.543746837 | 0.006289215 | 0.321959344 | 2.47038 | 0.34718 |
| GaJi4-3105 | 32.97221765 | 2.463709054 | 0.029027328 | 1.132055479 | 5.26824 | 1.29911 |
| GaJi4-3105 | 33.79814175 | 3.235578366 | 0.029750033 | 1.873896295 | 6.69522 | 1.7907 |
| GaJi4-3105 | 34.89168858 | 10.84736688 | 0.147136099 | 5.524038101 | 21.38406 | 7.85056 |
| GaJi4-3105 | 33.41828755 | 6.451940655 | 0.298294949 | 3.411598162 | 10.386 | 3.13516 |
| GaJi4-3105 | 30.22891779 | 0.258820747 | 0.001970619 | 0.131740835 | 1.40981 | 0.11366 |
| GaJi12-1269 | 54.02062112 | 0.543124817 | 0.005806637 | 0.274193768 | 2.64241 | 0.42005 |
| GaJi12-1269 | 57.46842493 | 0.28956422 | 0.000839104 | 0.143225974 | 1.94485 | 0.22396 |
| GaJi12-1269 | 58.45069897 | 0.77486725 | 0.014754275 | 0.373245749 | 4.25615 | 0.62653 |
| GaJi12-1269 | 58.80516504 | 0.12073749 | 0.000369616 | 0.05472601 | 1.2794 | 0.105 |
| GaJi12-1269 | 58.83849357 | 0.097331364 | 0.000510268 | 0.050835929 | 1.17502 | 0.07702 |
| GaJi12-1269 | 58.76371998 | 0.683841343 | 0.010517214 | 0.318384419 | 3.02516 | 0.42291 |
| GaJi12-1269 | 58.42534773 | 3.458955956 | 0.050167843 | 1.574077006 | 8.10748 | 2.90277 |
| GaJi12-1269 | 58.98888177 | 6.487295513 | 0.081275234 | 3.039279998 | 11.41278 | 5.49697 |
| GaJi12-1269 | 58.88549015 | 1.553156971 | 0.013292014 | 0.705851391 | 4.84318 | 1.27575 |
| GaJi12-1269 | 58.15131946 | 4.007268699 | 0.112965834 | 1.731164188 | 9.57619 | 3.52814 |
| GaJi12-1269 | 57.70239529 | 0.606704662 | 0.008263315 | 0.284240638 | 2.67877 | 0.44101 |
| GaJi12-1269 | 57.55698682 | 0.515818824 | 0.010992628 | 0.255464923 | 2.65932 | 0.4673 |
| GaJi12-1269 | 56.76463592 | 1.93093098 | 0.046908826 | 0.959448714 | 5.65245 | 1.64332 |
| GaJi12-1269 | 52.54706429 | 0.257343398 | 0.003479583 | 0.128428302 | 1.8551 | 0.20708 |
| GaJi12-1269 | 52.99053627 | 0.099094565 | 0.001453636 | 0.050017581 | 1.15795 | 0.084 |
| FxJj12N-170 | 22.67856918 | 0.124620489 | 0.00089339 | 0.063053482 | 1.0501 | 0.06999 |
| FxJj12N-170 | 22.56299279 | 0.6674426 | 0.035783679 | 0.412872174 | 1.70059 | 0.16284 |
| FxJj12N-170 | 22.70230042 | 0.224710664 | 0.002299484 | 0.123188564 | 1.48486 | 0.13124 |
| FxJj12N-170 | 23.28607865 | 1.568164363 | 0.021105008 | 0.866907954 | 0.93364 | 0.0557 |
| FxJj12N-170 | 22.65501757 | 0.063389417 | 0.000357267 | 0.033173376 | 0.76384 | 0.03481 |
| FxJj12N-170 | 22.80513136 | 0.033940238 | 0.000156109 | 0.018447173 | 0.64708 | 0.02388 |
| FxJj12N-170 | 22.58940979 | 0.587206037 | 0.004457315 | 0.328018722 | 0.64306 | 0.02307 |
| FxJj12N-170 | 24.0359465 | 1.115891035 | 0.02519298 | 0.622345618 | 0.92984 | 0.05438 |
| FxJj12N-170 | 24.4625456 | 1.02900341 | 0.01200899 | 0.56953743 | 0.76285 | 0.03185 |
| FxJj12N-170 | 27.74776107 | 0.022397695 | 8.68328E-05 | 0.011389955 | 0.5275 | 0.01194 |
| FxJj12N-170 | 27.55648958 | 0.726792121 | 0.006878476 | 0.404746539 | 0.76271 | 0.03183 |
| FxJj12N-170 | 27.25059539 | 7.974756118 | 0.773506267 | 4.848208879 | 15.39444 | 3.58405 |
| FxJj12N-170 | 27.87388498 | 0.233365593 | 0.00175981 | 0.129279363 | 0.50174 | 0.01194 |
| FxJj12N-170 | 27.86537059 | 0.733076781 | 0.004186061 | 0.412482762 | 0.72721 | 0.03017 |
| FxJj12N-170 | 28.21063176 | 0.29688082 | 0.009287187 | 0.161807723 | 0.8527 | 0.03183 |
| FxJj12N-170 | 28.56078506 | 0.303791757 | 0.003186102 | 0.168909819 | 0.50139 | 0.01327 |
| FxJj12N-170 | 28.78502447 | 0.606121414 | 0.006621545 | 0.336561727 | 0.64703 | 0.02388 |
| FxJj12N-170 | 29.12032042 | 2.875542825 | 0.03595269 | 1.59660348 | 1.56728 | 0.14728 |
| FxJj12N-170 | 29.17059922 | 0.398286221 | 0.00376759 | 0.218756961 | 0.66262 | 0.02122 |
| FxJj12N-170 | 29.19149471 | 0.257017693 | 0.005513359 | 0.142011682 | 0.65523 | 0.01724 |
| FxJj12N-170 | 29.3183395 | 0.489969655 | 0.00734325 | 0.271296816 | 0.68328 | 0.02885 |
| FxJj12N-170 | 29.16809525 | 0.751415025 | 0.030828745 | 0.42526459 | 1.61782 | 0.08621 |
| FxJj12N-170 | 29.34559663 | 1.192001477 | 0.009426257 | 0.662275503 | 1.15511 | 0.06501 |
| FxJj12N-170 | 29.17312706 | 2.065763017 | 0.050975016 | 1.15131708 | 2.12791 | 0.12816 |
| FxJj12N-170 | 29.45674027 | 2.016139533 | 0.020875031 | 1.119769607 | 1.45395 | 0.12026 |
| FxJj12N-170 | 29.40995581 | 1.878865884 | 0.025024668 | 1.034516321 | 1.51214 | 0.10217 |
| FxJj12N-170 | 29.46986316 | 1.353823968 | 0.011548944 | 0.7506328 | 1.16266 | 0.0796 |
| FxJj12N-170 | 29.09065074 | 1.974802519 | 0.13199883 | 1.241718197 | 4.13861 | 0.44528 |
| FxJj12N-170 | 29.51207538 | 0.41713051 | 0.005038952 | 0.232103537 | 0.62262 | 0.02283 |
| FxJj12N-170 | 29.51931988 | 0.45173868 | 0.004230084 | 0.243047576 | 0.78666 | 0.02389 |
| FxJj12N-170 | 29.36346932 | 0.621764026 | 0.016890139 | 0.347752891 | 0.81835 | 0.04643 |
| FxJj12N-170 | 29.26950708 | 0.944232879 | 0.016837086 | 0.526660523 | 0.8407 | 0.04644 |
| FxJj12N-170 | 29.32556157 | 0.663436869 | 0.017512104 | 0.369158989 | 0.78438 | 0.04378 |
| FxJj12N-170 | 29.55034155 | 1.570525991 | 0.016181985 | 0.862557877 | 1.26606 | 0.10878 |
| FxJj12N-170 | 29.60208548 | 0.366495188 | 0.00344476 | 0.201644975 | 0.66345 | 0.02521 |
| FxJj12N-170 | 29.50653898 | 4.235041037 | 0.07239199 | 2.344899867 | 3.08822 | 0.27531 |
| FxJj12N-170 | 29.62542682 | 1.18883017 | 0.015254644 | 0.657068124 | 1.91155 | 0.07154 |
| FxJj12N-170 | 29.6767309 | 0.937748525 | 0.011937989 | 0.526912681 | 1.23243 | 0.0796 |
| FxJj12N-170 | 29.55756251 | 0.571513048 | 0.005692101 | 0.314564539 | 0.86063 | 0.04908 |
| FxJj12N-170 | 29.76889139 | 0.097504143 | 0.002752555 | 0.05604224 | 0.5315 | 0.01459 |
| FxJj12N-170 | 29.59926388 | 0.052032318 | 0.000193045 | 0.025953773 | 0.78017 | 0.02839 |
| FxJj12N-170 | 29.28680751 | 0.999949963 | 0.020992346 | 0.559935729 | 1.07219 | 0.05041 |
| FxJj12N-170 | 29.7683181 | 0.552963242 | 0.00657057 | 0.319541511 | 1.4368 | 0.04442 |
| FxJj12N-170 | 29.52535419 | 1.636617574 | 0.046894975 | 0.917085398 | 1.5021 | 0.12434 |
| FxJj12N-170 | 29.73216519 | 0.188851012 | 0.005875185 | 0.103764888 | 0.61731 | 0.02123 |
| FxJj12N-170 | 29.51791866 | 0.086171092 | 0.002432051 | 0.065296895 | 1.04862 | 0.0374 |
| FxJj12N-170 | 29.68268156 | 9.842734189 | 0.053280695 | 5.443215754 | 5.42857 | 0.50175 |
| FxJj12N-170 | 29.5932554 | 0.393137138 | 0.002625198 | 0.217448149 | 0.84325 | 0.03613 |
| FxJj12N-170 | 29.75021671 | 0.428345646 | 0.014454295 | 0.254527148 | 1.60872 | 0.07529 |
| FxJj12N-170 | 29.36445015 | 0.03504664 | 0.000150593 | 0.017532369 | 0.66283 | 0.01989 |
| FxJj12N-170 | 29.45234615 | 0.593064318 | 0.010780144 | 0.330837362 | 0.90192 | 0.03482 |
| FxJj12N-170 | 29.70212908 | 0.936781017 | 0.009594628 | 0.526880836 | 0.8242 | 0.04246 |
| FxJj12N-170 | 29.4119271 | 0.563452765 | 0.011160718 | 0.318623334 | 0.8443 | 0.03881 |
| FxJj12N-170 | 29.66926076 | 0.234067178 | 0.002191363 | 0.128856083 | 0.48 | 0.01061 |
| FxJj12N-170 | 29.35648291 | 0.025332056 | 8.04956E-05 | 0.012527652 | 0.51528 | 0.01326 |
| FxJj12N-170 | 29.57632013 | 12.40026482 | 0.24945542 | 6.906757456 | 8.30244 | 0.8197 |
| FxJj12N-170 | 29.54285916 | 0.57380481 | 0.007124976 | 0.315760779 | 0.77469 | 0.02786 |
| FxJj12N-170 | 29.69936173 | 11.57197027 | 0.121203078 | 6.405394979 | 5.92473 | 0.54457 |
| FxJj12N-170 | 29.48650447 | 0.047936689 | 0.000198942 | 0.023597085 | 0.66283 | 0.01989 |
| FxJj12N-170 | 29.18549884 | 1.080191098 | 0.020046699 | 0.610995015 | 1.1746 | 0.06234 |
| FxJj12N-170 | 29.6511865 | 0.365040771 | 0.005424069 | 0.197773416 | 0.65474 | 0.0262 |
| FxJj12N-170 | 29.67212297 | 2.699271077 | 0.031330099 | 1.500971786 | 2.0012 | 0.14889 |
| FxJj12N-170 | 29.73166062 | 7.132726838 | 0.075909049 | 3.971943307 | 5.26165 | 0.4664 |
| FxJj12N-170 | 29.66646284 | 0.255240899 | 0.003539254 | 0.141399282 | 0.58676 | 0.01923 |
| FxJj12N-170 | 29.84395439 | 0.086380128 | 0.001815956 | 0.060729188 | 1.06046 | 0.03189 |
| FxJj12N-170 | 29.63048634 | 1.593923074 | 0.020882651 | 0.880234124 | 2.01716 | 0.08755 |
| FxJj12N-170 | 29.41490916 | 3.428188805 | 0.061231995 | 1.907780142 | 2.26173 | 0.20135 |
| FxJj12N-170 | 29.62183607 | 0.162645274 | 0.006882394 | 0.096829931 | 0.79657 | 0.02919 |
| FxJj12N-170 | 29.19080681 | 3.867497268 | 0.088131208 | 2.156189652 | 2.54075 | 0.24668 |
| FxJj12N-170 | 29.34663338 | 0.302213564 | 0.007884203 | 0.167074951 | 0.6427 | 0.02388 |
| FxJj12N-170 | 29.42061953 | 0.731353364 | 0.011921559 | 0.406434314 | 1.02014 | 0.04477 |
| FxJj12N-170 | 29.51805612 | 0.694675537 | 0.005833618 | 0.387892771 | 1.09082 | 0.0427 |
| FxJj12N-170 | 29.51920982 | 0.752368547 | 0.007997421 | 0.422042669 | 1.40542 | 0.04773 |
| FxJj12N-170 | 29.5814164 | 4.453695559 | 0.049117279 | 2.464303428 | 2.20346 | 0.30246 |
| FxJj12N-170 | 29.37243039 | 1.271600154 | 0.020548302 | 0.705407016 | 1.6643 | 0.08283 |
| FxJj12N-170 | 29.45957338 | 1.664208333 | 0.018658027 | 0.911572296 | 1.46574 | 0.10977 |
| FxJj12N-170 | 29.35450442 | 1.732469624 | 0.06966235 | 1.115221106 | 3.80424 | 0.42318 |
| FxJj12N-170 | 29.42322361 | 0.52776305 | 0.004929234 | 0.299212426 | 0.83289 | 0.0292 |
| FxJj12N-170 | 29.42219623 | 0.330919925 | 0.002616761 | 0.181062983 | 0.65523 | 0.01724 |
| FxJj12N-170 | 27.18840363 | 0.927402281 | 0.024581709 | 0.489759927 | 3.07498 | 0.37012 |
| FxJj12N-170 | 29.43272376 | 2.084076712 | 0.020125068 | 1.165297251 | 2.26497 | 0.13262 |
| FxJj12N-170 | 29.2431027 | 0.540511294 | 0.008711971 | 0.292754683 | 0.78267 | 0.03582 |
| FxJj12N-170 | 29.31109856 | 0.754212731 | 0.007104256 | 0.417149842 | 0.91452 | 0.03947 |
| FxJj12N-170 | 29.27507388 | 1.065103352 | 0.027610212 | 0.600660506 | 1.39309 | 0.11804 |
| FxJj12N-170 | 29.321136 | 0.391872292 | 0.00251298 | 0.218411485 | 0.68301 | 0.02122 |
| FxJj12N-170 | 29.47289664 | 0.330234105 | 0.003261517 | 0.162758811 | 1.83958 | 0.21059 |
| FxJj12N-170 | 24.5978105 | 0.571642355 | 0.005124446 | 0.318434249 | 0.66283 | 0.01989 |
| FxJj12N-170 | 22.24132399 | 3.234774708 | 0.03067513 | 1.786209874 | 1.2152 | 0.06298 |
| FxJj12N-170 | 24.14372458 | 1.212659133 | 0.00844854 | 0.656743114 | 0.97203 | 0.03582 |
| FxJj12N-170 | 23.65054468 | 1.201036169 | 0.01288383 | 0.658286928 | 0.97851 | 0.03814 |
| FxJj12N-170 | 23.80230103 | 0.772637609 | 0.004205404 | 0.426717596 | 0.66301 | 0.0199 |
| PrimeArch-1206 | 73.15479276 | 0.117108779 | 0.000950858 | 0.061228037 | 1.23792 | 0.09831 |
| PrimeArch-1206 | 73.14577515 | 1.139589865 | 0.024896189 | 0.580569839 | 4.60804 | 0.90389 |
| PrimeArch-1206 | 72.32935279 | 0.060549331 | 0.000479863 | 0.031176757 | 0.98381 | 0.05462 |
| PrimeArch-1206 | 72.20078725 | 0.722072672 | 0.011426216 | 0.375487902 | 2.82202 | 0.45221 |
| PrimeArch-1206 | 72.27154934 | 0.137305475 | 0.000559768 | 0.066641482 | 1.21141 | 0.07428 |
| PrimeArch-1206 | 73.53362312 | 0.135775139 | 0.000992405 | 0.073071465 | 1.49677 | 0.12673 |
| PrimeArch-1206 | 69.38282064 | 1.698938076 | 0.026759416 | 0.858703416 | 4.72744 | 1.09077 |
| PrimeArch-1206 | 73.71609432 | 1.135129086 | 0.010815334 | 0.57062031 | 5.1125 | 1.02571 |
| PrimeArch-1206 | 72.22467195 | 1.401453069 | 0.046283892 | 0.640390293 | 4.70167 | 1.11807 |
| PrimeArch-1206 | 69.91098186 | 0.79627937 | 0.013469692 | 0.4836493 | 3.81342 | 0.56528 |
| PrimeArch-1206 | 70.92640712 | 0.212691295 | 0.00225226 | 0.111505621 | 1.63452 | 0.17694 |
| PrimeArch-1206 | 72.46465656 | 6.054735979 | 0.181859184 | 2.983979624 | 13.25786 | 4.25579 |
| PrimeArch-1206 | 73.703479 | 0.872982077 | 0.018141713 | 0.455361322 | 3.78335 | 0.75312 |
| PrimeArch-1206 | 70.74502408 | 0.661552483 | 0.019528826 | 0.303262569 | 3.73107 | 0.54126 |
| PrimeArch-1206 | 70.94155238 | 5.137454425 | 0.102091428 | 2.727692307 | 7.70603 | 2.9422 |
| PrimeArch-1206 | 73.8760536 | 0.434481653 | 0.009266605 | 0.236443168 | 2.49293 | 0.39056 |
| PrimeArch-1206 | 73.76758933 | 1.26626353 | 0.026776226 | 0.681498894 | 4.37377 | 1.0776 |
| PrimeArch-1206 | 73.93719072 | 3.369041646 | 0.088195262 | 1.777945209 | 8.22984 | 2.85337 |
| PrimeArch-1206 | 73.80597661 | 0.810773683 | 0.011298691 | 0.528579249 | 3.44477 | 0.73894 |
| PrimeArch-1206 | 70.37897222 | 0.792887486 | 0.009734114 | 0.423439383 | 3.38014 | 0.5986 |
| PrimeArch-1206 | 73.7145097 | 0.493148258 | 0.00703978 | 0.303817265 | 2.85965 | 0.43201 |
| PrimeArch-1206 | 73.5520129 | 0.464290115 | 0.004568322 | 0.237434453 | 1.95311 | 0.25942 |
| PrimeArch-1206 | 73.67385794 | 11.00177623 | 0.506798135 | 5.726114602 | 28.3263 | 8.27024 |
| PrimeArch-1206 | 73.82810579 | 6.028622084 | 0.121390941 | 3.285333314 | 14.98642 | 5.04722 |
| PrimeArch-1206 | 70.15784774 | 2.233835987 | 0.05193393 | 1.058189726 | 6.44273 | 1.79205 |
| PrimeArch-1206 | 69.22765997 | 0.207273939 | 0.001742223 | 0.109546889 | 1.53352 | 0.15074 |
| PrimeArch-1206 | 73.72908641 | 1.601690416 | 0.025991021 | 0.606612862 | 4.97397 | 1.34943 |
| PrimeArch-1206 | 68.99213364 | 0.097699773 | 0.000563547 | 0.051006183 | 0.94643 | 0.05898 |
| PrimeArch-1206 | 73.5585735 | 0.313548842 | 0.006604264 | 0.188435332 | 2.17398 | 0.28839 |
| PrimeArch-1206 | 68.66152473 | 2.188186718 | 0.066050322 | 0.993124391 | 6.46296 | 1.57525 |
| PrimeArch-1206 | 69.20414433 | 0.168598455 | 0.000922719 | 0.08571818 | 1.36408 | 0.11798 |
| PrimeArch-1206 | 69.14668606 | 0.346223482 | 0.002940594 | 0.18098604 | 1.6115 | 0.16931 |
| PrimeArch-1206 | 73.51179672 | 2.902741902 | 0.07685446 | 1.480768416 | 8.66587 | 2.45669 |
| PrimeArch-1206 | 73.42052951 | 0.729136651 | 0.010340643 | 0.369706728 | 3.25556 | 0.5893 |
| PrimeArch-1206 | 69.02439513 | 0.506381657 | 0.005034978 | 0.292207167 | 1.59471 | 0.14581 |
| PrimeArch-1206 | 67.79008997 | 0.116226374 | 0.000981807 | 0.056190705 | 1.22772 | 0.08519 |
| PrimeArch-1206 | 66.73168141 | 0.123064037 | 0.000495192 | 0.050487049 | 1.13692 | 0.07209 |
| PrimeArch-1206 | 67.6948576 | 0.281395164 | 0.001795726 | 0.134220331 | 1.87957 | 0.22613 |
| PrimeArch-1206 | 69.33397439 | 7.737063951 | 0.188749733 | 3.245192994 | 17.48435 | 5.42623 |
| PrimeArch-1206 | 67.89325234 | 0.629741143 | 0.009116403 | 0.27926026 | 2.80734 | 0.45882 |
| PrimeArch-1206 | 72.98792495 | 2.1960726 | 0.114993441 | 1.389462721 | 7.93449 | 1.94389 |
| PrimeArch-1206 | 68.97265034 | 2.351687295 | 0.060475489 | 1.415067346 | 7.50368 | 1.96132 |
| PrimeArch-1206 | 72.64922219 | 0.105420207 | 0.001708901 | 0.05597637 | 1.20259 | 0.07865 |
| PrimeArch-1206 | 72.47932487 | 0.590387655 | 0.019963291 | 0.253490876 | 2.9403 | 0.46314 |
| PrimeArch-1206 | 67.24849752 | 0.018471633 | 7.89787E-05 | 0.009895498 | 0.50108 | 0.01638 |
| PrimeArch-1206 | 68.89208657 | 2.757092387 | 0.055241997 | 1.179646152 | 7.03946 | 2.22227 |
| PrimeArch-1206 | 69.14852027 | 5.13863141 | 0.085403256 | 2.83034592 | 12.7915 | 3.106 |
| PrimeArch-1206 | 68.92365484 | 2.854118695 | 0.070540944 | 1.352789148 | 7.9211 | 2.21644 |
| PrimeArch-1206 | 67.5283222 | 0.133201319 | 0.000523384 | 0.053201315 | 1.12041 | 0.07864 |
| PrimeArch-1206 | 68.75058253 | 1.13591875 | 0.036247235 | 0.701607096 | 4.69655 | 0.92739 |
| PrimeArch-1206 | 67.53403981 | 0.973450653 | 0.013038595 | 0.547575566 | 3.22816 | 0.59093 |
| PrimeArch-1206 | 66.47147238 | 0.077274538 | 0.000778171 | 0.042505261 | 1.13707 | 0.07211 |
| PrimeArch-1206 | 68.56517249 | 0.432692536 | 0.004949175 | 0.224319265 | 2.34459 | 0.3041 |
| PrimeArch-1206 | 67.20036043 | 0.095920984 | 0.000813666 | 0.047944318 | 1.20259 | 0.07864 |
| PrimeArch-1206 | 68.64797278 | 3.321470961 | 0.139510262 | 2.055858789 | 10.1096 | 2.45556 |
| PrimeArch-1206 | 68.13864057 | 1.478413468 | 0.028876215 | 0.850014315 | 5.20392 | 1.08141 |
| PrimeArch-1206 | 68.63547038 | 3.158870674 | 0.061424589 | 1.755562515 | 6.73671 | 2.15894 |
| PrimeArch-1206 | 68.99337273 | 0.220883453 | 0.003849134 | 0.106669763 | 1.54885 | 0.14421 |
| PrimeArch-1206 | 68.66517782 | 0.393170568 | 0.003042805 | 0.187663824 | 2.1211 | 0.23484 |
| PrimeArch-1206 | 69.10604533 | 0.779374964 | 0.013555087 | 0.407638316 | 2.10071 | 0.31581 |
| PrimeArch-1206 | 69.07843024 | 4.038434227 | 0.122406169 | 2.415522689 | 9.65181 | 3.30974 |
| PrimeArch-1206 | 67.12384166 | 0.234777942 | 0.002244032 | 0.127076931 | 1.77231 | 0.17041 |
| PrimeArch-1206 | 69.18088054 | 0.066235372 | 0.000467454 | 0.033098346 | 0.94189 | 0.05942 |
| PrimeArch-1206 | 69.3111304 | 2.730732913 | 0.049846868 | 1.400294844 | 6.54902 | 2.0694 |
| PrimeArch-1206 | 69.26086044 | 7.155951558 | 0.095942504 | 3.685035117 | 11.48593 | 4.90301 |
| PrimeArch-1206 | 68.42632714 | 0.397854232 | 0.00787641 | 0.196059656 | 2.01359 | 0.23105 |
| PrimeArch-1206 | 67.30917617 | 0.623840683 | 0.006504344 | 0.332826326 | 2.93006 | 0.43533 |
| PrimeArch-1206 | 69.09774839 | 1.814715801 | 0.01749659 | 0.900604603 | 7.4391 | 1.56268 |
| PrimeArch-1206 | 68.81598935 | 1.936450436 | 0.037838752 | 1.021676541 | 5.58709 | 1.06731 |
| PrimeArch-1263 | 67.76566331 | 0.327020417 | 0.002811832 | 0.166922381 | 2.23311 | 0.32329 |
| PrimeArch-1263 | 67.06981861 | 0.198136238 | 0.003466538 | 0.10053288 | 1.83891 | 0.18802 |
| PrimeArch-1263 | 66.21127752 | 0.83466923 | 0.015238384 | 0.350706689 | 3.75949 | 0.81153 |
| PrimeArch-1263 | 68.91656172 | 0.61418474 | 0.011772111 | 0.316470791 | 3.24447 | 0.59377 |
| PrimeArch-1263 | 69.86718143 | 1.386989333 | 0.041339852 | 0.742862629 | 6.30932 | 1.33769 |
| PrimeArch-1263 | 70.04722775 | 0.527183634 | 0.016476907 | 0.194556188 | 3.45741 | 0.47668 |
| PrimeArch-1263 | 70.18510221 | 0.104694855 | 0.001559847 | 0.054316554 | 1.24197 | 0.09895 |
| PrimeArch-1263 | 70.48475073 | 2.358866459 | 0.066313132 | 1.526444499 | 6.26621 | 2.28542 |
| PrimeArch-1263 | 70.31690039 | 0.546078406 | 0.010947515 | 0.217439598 | 3.65938 | 0.53162 |
| PrimeArch-1263 | 70.64810788 | 2.577459477 | 0.07936776 | 1.421808721 | 6.70396 | 2.4768 |
| PrimeArch-1263 | 70.86464668 | 2.460295632 | 0.044385252 | 1.075873311 | 7.26069 | 2.39867 |
| PrimeArch-1263 | 70.16433007 | 0.084330556 | 0.001016088 | 0.043925624 | 1.12543 | 0.07916 |
| PrimeArch-1263 | 69.77165341 | 2.680601538 | 0.11458189 | 1.11199717 | 9.69236 | 2.55316 |
| PrimeArch-1263 | 68.57902453 | 0.736812194 | 0.012591782 | 0.367467173 | 3.69562 | 0.70977 |
| PrimeArch-1263 | 65.81775818 | 0.810840766 | 0.026772064 | 0.477176816 | 3.67653 | 0.75208 |
| PrimeArch-1263 | 63.90914941 | 0.089349833 | 0.001056866 | 0.047455681 | 1.10857 | 0.08353 |
| PrimeArch-1263 | 63.85476923 | 0.497932433 | 0.015196432 | 0.209391382 | 3.47075 | 0.74326 |
| PrimeArch-1263 | 65.15084927 | 0.51443288 | 0.008579274 | 0.265052386 | 3.39646 | 0.49481 |

**Blue Polygons**

| FxJj10-553 | 71.06571029 | 0.898461052 | 0.016173065 | 0.568391007 | 4.69156 | 0.66879 |
| --- | --- | --- | --- | --- | --- | --- |
| FxJj10-553 | 71.86074383 | 1.329500095 | 0.02274436 | 0.837792762 | 5.05713 | 0.90268 |
| FxJj10-553 | 73.09693165 | 0.024716115 | 7.06986E-05 | 0.013211023 | 0.62412 | 0.01671 |
| FxJj10-553 | 73.11443489 | 0.077007609 | 0.000247416 | 0.040185448 | 0.99006 | 0.05016 |
| FxJj10-553 | 72.82098815 | 3.137207463 | 0.018914822 | 1.565909697 | 7.18862 | 1.50462 |
| FxJj10-553 | 73.1091029 | 0.100916869 | 0.000411557 | 0.050721232 | 0.99006 | 0.05016 |
| FxJj10-553 | 73.16821849 | 0.035396547 | 7.18407E-05 | 0.019087083 | 0.7314 | 0.03343 |
| FxJj10-553 | 71.65154034 | 3.896624461 | 0.063575979 | 1.803659837 | 7.40259 | 2.40728 |
| FxJj10-553 | 72.92759032 | 0.17510871 | 0.000787854 | 0.082117896 | 1.61421 | 0.13373 |
| FxJj10-553 | 72.5347212 | 2.532398339 | 0.043738392 | 1.551406192 | 7.55391 | 1.57139 |
| FxJj10-553 | 72.00335688 | 1.997621817 | 0.033853467 | 1.009387272 | 5.31582 | 1.08664 |
| FxJj10-553 | 70.46544631 | 3.749405523 | 0.065017842 | 2.144792083 | 8.65151 | 2.17338 |
| FxJj10-553 | 69.78684185 | 7.446780702 | 0.277782311 | 4.405034185 | 22.38607 | 5.51687 |
| FxJj10-553 | 69.99255432 | 0.047076867 | 0.000208828 | 0.020407406 | 0.88295 | 0.03344 |
| FxJj10-553 | 70.81268555 | 1.385483183 | 0.025119845 | 0.791371642 | 5.57422 | 0.90276 |
| FxJj10-553 | 66.61331038 | 0.046955205 | 0.000317866 | 0.0234326 | 0.88281 | 0.03342 |
| FxJj11-74 | 70.95635886 | 0.09883523 | 0.000584934 | 0.046604125 | 0.98701 | 0.04985 |
| FxJj11-74 | 69.89655547 | 0.089090202 | 0.000682563 | 0.046871709 | 0.88022 | 0.03323 |
| FxJj11-74 | 69.70856844 | 3.441610125 | 0.037285388 | 1.731706479 | 9.35449 | 2.32633 |
| FxJj11-74 | 71.89144926 | 0.066380037 | 0.000230157 | 0.034739431 | 0.98701 | 0.04985 |
| FxJj11-74 | 71.83259938 | 0.101149022 | 0.000626918 | 0.04776405 | 1.0938 | 0.06647 |
| FxJj11-74 | 72.21375561 | 0.016934866 | 5.60137E-05 | 0.007978827 | 0.62241 | 0.01662 |
| FxJj11-74 | 72.27074268 | 0.07794057 | 0.000425678 | 0.032062596 | 1.0938 | 0.06647 |
| FxJj11-74 | 72.34406459 | 0.151190663 | 0.001127267 | 0.077151832 | 1.4584 | 0.0997 |
| FxJj11-74 | 72.05361392 | 3.406724324 | 0.042750253 | 1.572893102 | 5.77104 | 1.36256 |
| FxJj11-74 | 68.49651063 | 1.81255317 | 0.035475393 | 0.967589944 | 5.29966 | 0.94715 |
| FxJj11-74 | 72.44914787 | 0.036753891 | 0.000191917 | 0.019177639 | 0.7292 | 0.03323 |
| FxJj11-74 | 71.60276323 | 1.171814134 | 0.012244683 | 0.593745702 | 3.43242 | 0.56497 |
| FxJj11-74 | 71.21302719 | 0.238030354 | 0.00103473 | 0.097785953 | 1.97402 | 0.13293 |
| FxJj11-74 | 70.20588677 | 0.16826379 | 0.001229855 | 0.077187343 | 1.97402 | 0.13293 |
| FxJj11-74 | 72.3371173 | 0.77976333 | 0.00654313 | 0.44831172 | 4.41943 | 0.58158 |
| FxJj11-74 | 72.57006726 | 0.064913836 | 0.000389639 | 0.034716775 | 0.98701 | 0.04985 |
| FxJj11-74 | 69.81978696 | 0.079300581 | 0.00034209 | 0.042658037 | 1.24482 | 0.06647 |
| FxJj11-74 | 69.87946839 | 4.393170009 | 0.054229872 | 1.977183927 | 12.1645 | 2.50911 |
| FxJj11-74 | 72.42372168 | 3.493656788 | 0.048822548 | 1.873823772 | 11.26596 | 1.96076 |
| FxJj11-74 | 72.02952695 | 0.105270117 | 0.001157867 | 0.043578275 | 1.35161 | 0.08308 |
| FxJj11-74 | 70.8202393 | 1.831375724 | 0.024026795 | 1.040372251 | 4.93506 | 1.27948 |
| FxJj11-74 | 71.31094758 | 1.004318505 | 0.012580616 | 0.53405247 | 5.04184 | 0.6979 |
| FxJj11-74 | 71.43653179 | 0.181864186 | 0.001883355 | 0.100546009 | 1.71621 | 0.11632 |
| FxJj11-74 | 69.25120281 | 7.425175446 | 0.092686968 | 3.848649397 | 5.40644 | 0.93053 |
| FxJj11-74 | 72.1404034 | 0.056102877 | 0.000211657 | 0.025160821 | 0.98701 | 0.04985 |
| FxJj11-74 | 72.14674709 | 0.018802799 | 2.52689E-05 | 0.009185775 | 0.62241 | 0.01662 |
| FxJj11-74 | 69.91790687 | 0.85055114 | 0.020587395 | 0.450520585 | 5.90374 | 0.71452 |
| FxJj11-74 | 71.7705935 | 0.040786886 | 0.000211605 | 0.020828161 | 0.7292 | 0.03323 |
| FxJj11-74 | 72.08607784 | 1.507210836 | 0.023340249 | 0.822578558 | 7.53149 | 1.0967 |
| FxJj11-74 | 70.09021524 | 0.207808318 | 0.001744652 | 0.109004615 | 2.1876 | 0.16617 |
| FxJj11-74 | 71.70817891 | 4.149170105 | 0.081303598 | 2.205386662 | 8.98989 | 2.2931 |
| FxJj11-74 | 71.85104234 | 0.24588402 | 0.002361397 | 0.139267685 | 1.24482 | 0.0997 |
| FxJj11-74 | 72.11021575 | 2.999717109 | 0.030937472 | 1.41802899 | 8.98989 | 1.66166 |
| FxJj11-74 | 70.53737254 | 0.969978033 | 0.005934538 | 0.488351579 | 3.11206 | 0.44865 |
| FxJj11-74 | 71.89306824 | 3.535592419 | 0.042388932 | 1.890817887 | 6.39346 | 1.47888 |
| FxJj11-74 | 72.21464148 | 0.106535863 | 0.000491888 | 0.061803547 | 1.60942 | 0.0997 |
| FxJj11-74 | 71.05778058 | 0.094819887 | 0.000877652 | 0.048151429 | 1.0938 | 0.06647 |
| FxJj11-74 | 70.5056441 | 0.610254774 | 0.006642802 | 0.304963607 | 3.2814 | 0.36557 |
| FxJj11-74 | 69.78329967 | 3.662699116 | 0.038056676 | 1.955108553 | 6.17988 | 1.97738 |
| FxJj11-74 | 70.57053234 | 1.078998562 | 0.012830002 | 0.556925928 | 4.82827 | 0.66467 |
| FxJj11-74 | 70.83150135 | 0.057533165 | 0.000366741 | 0.029596831 | 0.98701 | 0.04985 |
| FxJj11-74 | 70.86535178 | 0.03775844 | 9.87857E-05 | 0.01820985 | 0.7292 | 0.03323 |
| FxJj11-74 | 70.43668716 | 3.917672324 | 0.042627891 | 2.041242834 | 10.59931 | 2.2931 |
| FxJj16-400 | 75.25955121 | 0.869042892 | 0.008190041 | 0.463756721 | 3.50173 | 0.45852 |
| FxJj16-400 | 75.24110423 | 0.399353581 | 0.002292375 | 0.210884322 | 2.11197 | 0.26027 |
| FxJj16-400 | 75.2396997 | 0.524765927 | 0.004587314 | 0.264449147 | 4.35396 | 0.37174 |
| FxJj16-400 | 75.37138446 | 0.093946541 | 0.000371934 | 0.052749331 | 1.07511 | 0.07436 |
| FxJj16-400 | 75.15765679 | 0.025297716 | 0.000170799 | 0.015027043 | 0.5374 | 0.01239 |
| FxJj16-400 | 75.12629677 | 1.314518646 | 0.010279548 | 0.636605383 | 5.83636 | 0.85508 |
| FxJj16-400 | 73.58154687 | 2.411438198 | 0.025900972 | 1.229155399 | 4.83757 | 1.07812 |
| FxJj16-400 | 75.44812995 | 0.200561227 | 0.001267183 | 0.10787442 | 1.70459 | 0.12392 |
| FxJj16-400 | 75.1034212 | 0.044765722 | 0.000175557 | 0.024432739 | 0.6298 | 0.02479 |
| FxJj16-400 | 75.07828613 | 0.854381258 | 0.008818849 | 0.450107717 | 3.63207 | 0.53283 |
| FxJj16-400 | 75.18517071 | 0.273515635 | 0.002507478 | 0.134641146 | 2.14993 | 0.1735 |
| FxJj16-400 | 74.47046867 | 0.42557115 | 0.003762865 | 0.244097823 | 2.77973 | 0.17347 |
| FxJj16-453 | 73.64257979 | 0.390365337 | 0.002506601 | 0.182822574 | 2.77928 | 0.32366 |
| FxJj16-453 | 73.75559032 | 0.049211211 | 0.000241155 | 0.024731196 | 0.7806 | 0.03808 |
| FxJj16-453 | 74.31358975 | 0.097096302 | 0.00017674 | 0.045649718 | 1.21808 | 0.09518 |
| FxJj16-453 | 73.31181644 | 2.980888839 | 0.064377309 | 1.51506109 | 9.94595 | 2.70341 |
| FxJj16-453 | 74.34382845 | 7.847956598 | 0.133536134 | 4.38062041 | 24.89101 | 6.53038 |
| FxJj16-453 | 72.87893551 | 2.105213212 | 0.030261203 | 1.363626709 | 6.84331 | 1.84665 |
| FxJj16-453 | 75.13894288 | 0.061308386 | 0.00013788 | 0.031880558 | 1.05648 | 0.05711 |
| FxJj16-453 | 75.23190378 | 0.180183879 | 0.000991256 | 0.0970114 | 2.22745 | 0.17139 |
| FxJj16-453 | 76.45409542 | 0.696739502 | 0.002676458 | 0.324082211 | 3.83594 | 0.64736 |
| FxJj16-453 | 76.72681901 | 0.104690327 | 0.00054542 | 0.054629284 | 1.44685 | 0.0952 |
| FxJj16-453 | 77.19884946 | 2.448649904 | 0.057970122 | 1.431844826 | 9.44161 | 2.07518 |
| FxJj16-549 | 68.71212099 | 0.093474623 | 0.001259706 | 0.047985138 | 1.54632 | 0.08798 |
| FxJj16-549 | 68.80139511 | 0.019621012 | 0.000140784 | 0.010503231 | 0.64051 | 0.0176 |
| FxJj16-549 | 67.26818833 | 0.035346475 | 5.80143E-05 | 0.018590354 | 0.7504 | 0.03519 |
| FxJj16-549 | 70.90101889 | 0.176829603 | 0.000632263 | 0.085147941 | 1.876 | 0.17597 |
| FxJj16-549 | 70.32995318 | 1.04443943 | 0.034104537 | 0.701356471 | 5.76456 | 1.00302 |
| FxJj16-549 | 69.96331326 | 3.110451036 | 0.045756919 | 1.736132722 | 11.23715 | 3.07945 |
| FxJj16-549 | 69.78806802 | 0.729773276 | 0.009083516 | 0.397721347 | 4.92312 | 0.72147 |
| FxJj16-549 | 70.09881651 | 0.113568225 | 0.002305296 | 0.057404169 | 1.65621 | 0.10558 |
| FxJj16-549 | 69.96527778 | 0.073825306 | 0.001080731 | 0.039436622 | 1.28101 | 0.07039 |
| FxJj16-549 | 69.86122304 | 1.354804782 | 0.036077758 | 0.707673099 | 6.78026 | 1.26698 |
| FxJj16-549 | 69.57271007 | 1.736195738 | 0.062431924 | 0.976465205 | 9.47104 | 1.65411 |
| FxJj16-549 | 69.33730966 | 0.127273777 | 0.001994218 | 0.060656568 | 1.76611 | 0.12318 |
| FxJj16-549 | 69.12817581 | 0.128800556 | 0.001812918 | 0.060901879 | 1.76611 | 0.12318 |
| FxJj16-552 | 77.18829864 | 0.024114594 | 0.00013648 | 0.010522966 | 0.58861 | 0.01486 |
| FxJj16-552 | 77.156073 | 0.208293872 | 0.001226565 | 0.089655033 | 1.62321 | 0.13378 |
| FxJj16-552 | 76.99352268 | 0.15122187 | 0.0008865 | 0.076515028 | 1.62335 | 0.10408 |
| FxJj16-552 | 77.56285709 | 1.587975643 | 0.017797163 | 0.677366608 | 9.39557 | 1.10014 |
| FxJj16-552 | 76.8449595 | 0.207447652 | 0.001054293 | 0.109179887 | 1.42148 | 0.13383 |
| FxJj16-552 | 76.85429362 | 0.14251272 | 0.000566949 | 0.07961245 | 1.27858 | 0.10409 |
| FxJj16-552 | 76.86416161 | 6.910533068 | 0.075313735 | 3.55547372 | 18.7566 | 4.78757 |
| FxJj16-552 | 77.30560443 | 0.556162435 | 0.00540091 | 0.317451102 | 4.8702 | 0.40149 |
| FxJj16-552 | 77.2143798 | 0.03981884 | 0.000242452 | 0.017620965 | 0.83256 | 0.02974 |
| FxJj16-552 | 77.23505012 | 0.10639967 | 0.00032054 | 0.046746297 | 1.52236 | 0.08922 |
| FxJj16-552 | 77.13864462 | 1.458438957 | 0.010943661 | 0.716220458 | 5.80387 | 1.01104 |
| FxJj16-552 | 75.45764736 | 0.815321302 | 0.008241282 | 0.427296891 | 3.08667 | 0.49061 |
| FxJj16-580 | 69.97114303 | 3.384883745 | 0.251989349 | 1.452193058 | 11.68789 | 2.4711 |
| FxJj16-580 | 76.03077734 | 0.021583491 | 7.98184E-05 | 0.009527444 | 0.6347 | 0.01728 |
| FxJj16-580 | 75.78985497 | 0.997663396 | 0.027736688 | 0.5897987 | 3.91731 | 0.70853 |
| FxJj16-580 | 74.57219335 | 1.66857428 | 0.019031386 | 0.901349033 | 3.91711 | 0.63934 |
| FxJj16-580 | 74.96498289 | 1.141006219 | 0.012281775 | 0.668558172 | 5.73118 | 0.82944 |
| FxJj16-580 | 73.96403911 | 1.621934891 | 0.018081421 | 0.872889847 | 5.44959 | 1.07141 |
| FxJj16-580 | 73.37396556 | 0.027419531 | 0.00010945 | 0.012861099 | 0.6347 | 0.01728 |
| FxJj16-580 | 72.80833494 | 0.09470402 | 0.001225297 | 0.053153256 | 0.6347 | 0.01728 |
| FxJj16-587 | 67.726452 | 2.531649924 | 0.071132992 | 1.269235207 | 7.37656 | 1.62897 |
| FxJj16-587 | 67.7693065 | 1.134116886 | 0.014321932 | 0.594145652 | 4.50296 | 0.8871 |
| FxJj16-587 | 69.31832655 | 0.046549387 | 0.000169548 | 0.019466673 | 0.86719 | 0.03226 |
| FxJj16-587 | 69.26557693 | 0.746064896 | 0.006301326 | 0.349059333 | 3.38199 | 0.38718 |
| FxJj16-587 | 69.67979849 | 0.512155843 | 0.004230323 | 0.229237677 | 3.88957 | 0.45159 |
| FxJj16-587 | 69.73077925 | 0.424800709 | 0.001945718 | 0.185763087 | 2.66319 | 0.32256 |
| FxJj16-587 | 69.80577983 | 0.12862598 | 0.001066173 | 0.059209804 | 1.58576 | 0.09681 |
| FxJj16-587 | 70.28710437 | 0.090813185 | 0.00040183 | 0.049421046 | 1.33159 | 0.08064 |
| FxJj16-587 | 71.48345619 | 0.069186762 | 0.00043393 | 0.041870629 | 1.0776 | 0.06451 |
| FxJj16-587 | 71.4669829 | 0.05045578 | 0.000170449 | 0.030206305 | 0.97239 | 0.04838 |
| FxJj16-587 | 74.61429609 | 1.534292696 | 0.046160333 | 0.768510427 | 5.68557 | 1.35476 |
| FxJj16-587 | 77.1104464 | 0.067988912 | 0.000475478 | 0.034897004 | 1.0776 | 0.06451 |
| FxJj16-587 | 76.83717621 | 0.115152988 | 0.000866547 | 0.054857955 | 1.69079 | 0.1129 |
| FxJj16-587 | 76.92399204 | 0.928085197 | 0.012129212 | 0.484922502 | 4.35418 | 0.91932 |
| FxJj16-587 | 75.38819318 | 1.442693851 | 0.049881562 | 0.739475543 | 5.26491 | 1.29027 |
| FxJj16-587 | 76.23000226 | 0.018507846 | 7.80751E-05 | 0.009247869 | 0.61319 | 0.01613 |
| FxJj16-680 | 70.80040276 | 0.101338604 | 0.002049076 | 0.048032849 | 1.2532 | 0.08726 |
| FxJj16-680 | 70.08344252 | 1.357433878 | 0.018335512 | 0.712390355 | 5.35935 | 1.13442 |
| FxJj16-680 | 70.84847704 | 0.0450486 | 9.23984E-05 | 0.027418947 | 0.8356 | 0.04364 |
| FxJj16-680 | 70.58025417 | 0.314353096 | 0.00637876 | 0.141879434 | 2.26206 | 0.26184 |
| FxJj16-680 | 70.82910726 | 0.022203737 | 7.64532E-05 | 0.011952618 | 0.71306 | 0.02181 |
| FxJj16-680 | 70.87572078 | 0.044809717 | 0.000244598 | 0.023296565 | 0.8356 | 0.04364 |
| FxJj16-680 | 70.92789898 | 1.443287936 | 0.013893544 | 0.642578682 | 4.57446 | 0.8945 |
| FxJj16-680 | 70.90075045 | 0.044349588 | 0.000191375 | 0.02362282 | 0.8354 | 0.04362 |
| FxJj16-680 | 69.94254949 | 0.099945515 | 0.00029619 | 0.048933633 | 1.2534 | 0.08728 |
| FxJj16-680 | 70.67040365 | 0.067441837 | 0.001546252 | 0.035420103 | 0.8354 | 0.04362 |
| FxJj16-680 | 70.76629552 | 1.151890304 | 0.063045341 | 0.544718861 | 5.06378 | 0.89447 |
| FxJj16-680 | 70.86443876 | 3.150612909 | 0.166410451 | 1.515169994 | 9.83275 | 2.15991 |
| FxJj16-680 | 69.51814068 | 0.035821248 | 0.00011583 | 0.014506357 | 0.71306 | 0.02181 |
| FxJj16-680 | 69.52309387 | 0.096087388 | 0.000601878 | 0.044621941 | 1.2534 | 0.08728 |
| FxJj16-680 | 70.16026185 | 0.687326553 | 0.006646033 | 0.386863083 | 3.09726 | 0.52352 |
| FxJj16-680 | 69.38031776 | 7.233941637 | 0.090040779 | 3.749871737 | 16.96441 | 5.34507 |
| FxJj16-680 | 69.60616812 | 0.025813277 | 0.00015929 | 0.012486543 | 0.71323 | 0.02182 |
| FxJj16-680 | 69.33327963 | 2.310893146 | 0.037323267 | 1.194201717 | 6.76487 | 1.72345 |
| FxJj16-680 | 69.68090918 | 0.022676327 | 6.86767E-05 | 0.011822837 | 0.71323 | 0.02182 |
| FxJj16-680 | 69.53914131 | 2.737314884 | 0.030247039 | 1.442739761 | 7.67192 | 2.02895 |
| FxJj16-680 | 69.43128612 | 0.145061466 | 0.001237296 | 0.067625402 | 1.6712 | 0.13092 |
| FxJj16-680 | 70.82112413 | 0.05488705 | 0.000168859 | 0.024330814 | 0.8356 | 0.04364 |
| FxJj16-680 | 70.80954242 | 0.047939574 | 0.000191839 | 0.022516976 | 0.8356 | 0.04364 |
| FxJj16-680 | 69.38044719 | 1.8816593 | 0.023661876 | 0.769571432 | 6.83652 | 1.24357 |
| FxJj16-680 | 69.05589148 | 2.545425505 | 0.038273437 | 1.192059393 | 6.24549 | 1.85447 |
| FxJj16-680 | 71.01506329 | 0.292352133 | 0.002270489 | 0.148651585 | 2.13969 | 0.24002 |
| FxJj16-680 | 71.02883707 | 1.782790407 | 0.026762601 | 1.023644918 | 8.38498 | 1.52722 |
| FxJj16-680 | 69.63582423 | 0.100081613 | 0.000848932 | 0.05178352 | 1.13103 | 0.06546 |
| FxJj16-680 | 71.88058175 | 0.050932408 | 0.000242399 | 0.025958749 | 0.8356 | 0.04364 |
| FxJj16-680 | 71.37228427 | 0.232094767 | 0.001235699 | 0.116105376 | 1.96643 | 0.15272 |
| FxJj16-680 | 71.21805687 | 0.068281895 | 0.000412829 | 0.039107555 | 1.13103 | 0.06546 |
| FxJj18GS-13-26 | 71.90587786 | 0.06635243 | 0.000227707 | 0.030631143 | 1.00668 | 0.04346 |
| FxJj18GS-13-26 | 71.7562265 | 0.185938755 | 0.001727904 | 0.099806239 | 1.00668 | 0.04346 |
| FxJj18GS-13-26 | 71.89577288 | 0.132080396 | 0.001267187 | 0.067980528 | 1.30148 | 0.06519 |
| FxJj18GS-13-26 | 71.98423027 | 0.033097502 | 0.000188065 | 0.017189089 | 0.71202 | 0.02175 |
| FxJj18GS-13-26 | 72.25264851 | 0.026533089 | 0.000128014 | 0.013749656 | 0.71202 | 0.02175 |
| FxJj18GS-13-26 | 72.33682806 | 0.13657932 | 0.001371384 | 0.057270276 | 1.59628 | 0.10864 |
| FxJj18GS-13-26 | 69.90202454 | 0.035416386 | 0.000201977 | 0.019425413 | 0.79823 | 0.03261 |
| FxJj18GS-13-26 | 71.23976393 | 0.588216116 | 0.005944397 | 0.315546231 | 1.80494 | 0.18474 |
| FxJj18GS-13-26 | 72.3449207 | 0.014901995 | 9.01263E-05 | 0.00879859 | 0.50326 | 0.01086 |
| FxJj18GS-13-26 | 71.25770917 | 4.771846389 | 0.096476948 | 2.306113792 | 14.00114 | 2.08658 |
| FxJj18GS-13-26 | 71.44731553 | 0.251256808 | 0.001430723 | 0.113345851 | 2.18608 | 0.17385 |
| FxJj18GS-13-26 | 72.14330651 | 0.315273405 | 0.001640609 | 0.15772102 | 1.42373 | 0.13041 |
| FxJj18GS-13-26 | 71.38985968 | 0.38321357 | 0.003903489 | 0.181237466 | 3.48773 | 0.28253 |
| FxJj18GS-13-26 | 71.39931508 | 0.708655941 | 0.009610568 | 0.352166507 | 2.43027 | 0.26082 |
| FxJj18GS-13-26 | 72.19290655 | 1.639953979 | 0.011234127 | 0.819110777 | 6.42131 | 0.94548 |
| FxJj18GS-13-26 | 72.36072691 | 1.028498537 | 0.01333468 | 0.481295536 | 5.37901 | 0.76073 |
| FxJj18GS-13-26 | 71.20453717 | 0.022088505 | 0.000152757 | 0.01154951 | 0.50326 | 0.01086 |
| FxJj18GS-13-26 | 71.22213817 | 0.437913426 | 0.004672757 | 0.251383751 | 2.27246 | 0.20649 |
| FxJj18GS-13-26 | 71.36499735 | 0.220747841 | 0.001937123 | 0.123932663 | 1.38783 | 0.09781 |
| FxJj18GS-13-26 | 71.69340174 | 3.437507432 | 0.042299552 | 1.795108123 | 6.12643 | 1.2715 |
| FxJj18GS-13-26 | 70.90101568 | 0.013579957 | 3.63451E-05 | 0.007125502 | 0.50326 | 0.01086 |
| FxJj18GS-13-26 | 71.89934879 | 0.179628903 | 0.001252666 | 0.086328547 | 1.4744 | 0.10871 |
| FxJj18GS-13-26 | 72.17132975 | 0.011494384 | 1.69518E-05 | 0.007971932 | 0.50343 | 0.01087 |
| FxJj18GS-13-26 | 71.85578651 | 0.044630952 | 0.000243747 | 0.027705548 | 0.79823 | 0.0326 |
| FxJj18GS-13-26 | 71.24485242 | 2.562086973 | 0.031658248 | 1.369511732 | 6.34996 | 1.27156 |
| FxJj18GS-13-26 | 72.14402256 | 0.013876269 | 5.83318E-05 | 0.007545465 | 0.50343 | 0.01087 |
| FxJj18GS-13-26 | 71.43150902 | 0.093897245 | 0.000697189 | 0.045087113 | 0.8846 | 0.04348 |
| FxJj18GS-13-26 | 71.54600543 | 0.032971165 | 7.55757E-05 | 0.017690441 | 0.79823 | 0.0326 |
| FxJj18GS-13-26 | 72.02035865 | 2.194725486 | 0.01693439 | 1.18812324 | 8.13996 | 1.35837 |
| FxJj18GS-13-26 | 72.1893116 | 0.032397722 | 0.000136875 | 0.016328282 | 0.5896 | 0.02173 |
| FxJj18GS-13-26 | 72.12778148 | 1.010847594 | 0.012374127 | 0.481730597 | 4.37216 | 0.63028 |
| FxJj18GS-13-26 | 71.51232876 | 0.336029838 | 0.003010446 | 0.186284858 | 2.48088 | 0.26085 |
| FxJj18GS-13-26 | 72.12232906 | 0.870221088 | 0.008673154 | 0.517711627 | 5.62315 | 0.5869 |
| FxJj18GS-13-26 | 71.77163763 | 0.030581733 | 0.000111903 | 0.011543968 | 0.71185 | 0.02174 |
| FxJj18GS-13-26 | 71.82047213 | 0.520734388 | 0.004541997 | 0.300364964 | 3.90441 | 0.43471 |
| FxJj18GS-2148 | 71.89232671 | 0.363491408 | 0.004955703 | 0.192683692 | 3.07871 | 0.33542 |
| FxJj18GS-2148 | 74.44026635 | 0.023341984 | 5.60713E-05 | 0.011875573 | 0.6084 | 0.02313 |
| FxJj18GS-2148 | 74.33819617 | 0.024984571 | 5.09492E-05 | 0.011977716 | 0.6082 | 0.02312 |
| FxJj18GS-2148 | 74.46741272 | 0.012041642 | 3.52182E-05 | 0.006471709 | 0.5193 | 0.01157 |
| FxJj18GS-2148 | 74.45215192 | 0.02322016 | 3.70634E-05 | 0.011790907 | 0.6082 | 0.02312 |
| FxJj18GS-2148 | 74.18942683 | 0.012068953 | 4.03783E-05 | 0.005420694 | 0.51913 | 0.01156 |
| FxJj18GS-2148 | 74.45069723 | 0.117387606 | 0.001086747 | 0.07196575 | 1.8248 | 0.1156 |
| FxJj18GS-2148 | 73.75825162 | 0.05942445 | 0.0003303 | 0.029436024 | 1.1277 | 0.05784 |
| FxJj18GS-2148 | 74.30775146 | 0.196649735 | 0.002846894 | 0.075624027 | 1.9508 | 0.16185 |
| FxJj18GS-2148 | 74.22156859 | 0.337953315 | 0.001348605 | 0.190151975 | 2.77433 | 0.31217 |
| FxJj18GS-2148 | 74.38696879 | 0.191413512 | 0.001331526 | 0.124097254 | 2.2552 | 0.18506 |
| FxJj18GS-5305 | 51.37842681 | 0.664032631 | 0.015233751 | 0.337953083 | 4.31798 | 0.55286 |
| FxJj18GS-5305 | 51.51921305 | 0.162180403 | 0.000804707 | 0.094577092 | 1.50613 | 0.14552 |
| FxJj18GS-5305 | 51.69290949 | 1.390335762 | 0.019205236 | 0.719466221 | 6.84737 | 1.04755 |
| FxJj18GS-5305 | 53.99799564 | 0.787413457 | 0.008473197 | 0.402926116 | 5.15885 | 0.62557 |
| FxJj18GS-5305 | 53.3329243 | 2.058051122 | 0.034305684 | 0.945424838 | 6.66455 | 1.61493 |
| FxJj18GS-5305 | 56.31190766 | 1.026170972 | 0.009786695 | 0.596057567 | 6.66475 | 0.82921 |
| FxJj18GS-5305 | 56.05975051 | 0.459297619 | 0.005985908 | 0.228839967 | 3.55279 | 0.40734 |
| FxJj18GS-5305 | 54.78449493 | 1.305007055 | 0.011368815 | 0.787873962 | 5.98258 | 0.94562 |
| FxJj18GS-5715 | 73.96680278 | 3.714587943 | 0.0422464 | 1.885620816 | 10.23317 | 1.75172 |
| FxJj18GS-5715 | 74.12696261 | 0.020311011 | 0.000101316 | 0.010972864 | 0.47492 | 0.00967 |
| FxJj18GS-5715 | 74.16601113 | 0.033579147 | 0.000207718 | 0.015924721 | 0.5564 | 0.01935 |
| FxJj18GS-5715 | 74.20348508 | 0.301790097 | 0.002863436 | 0.153893228 | 2.42292 | 0.20322 |
| FxJj18GS-5715 | 74.39089761 | 0.136045945 | 0.001049904 | 0.066527946 | 1.7848 | 0.11614 |
| FxJj18GS-5715 | 74.70953734 | 0.022950119 | 4.28898E-05 | 0.013732105 | 0.6718 | 0.01936 |
| FxJj18GS-5715 | 74.74665634 | 0.247531825 | 0.002270376 | 0.113986516 | 2.25992 | 0.18392 |
| FxJj18GS-5715 | 74.63272549 | 0.058060451 | 0.000217508 | 0.031335194 | 0.75312 | 0.02902 |
| FxJj18GS-5715 | 74.77425517 | 0.162709528 | 0.000870209 | 0.075419741 | 2.06303 | 0.13547 |
| FxJj18GS-5715 | 71.50389738 | 0.023051659 | 0.000134708 | 0.011797609 | 0.5566 | 0.01936 |
| FxJj18GS-5715 | 71.57352665 | 2.70432374 | 0.022629239 | 1.619467913 | 7.41725 | 1.87745 |
| FxJj18GS-5715 | 71.50867378 | 0.041115906 | 0.00015008 | 0.018280713 | 0.6718 | 0.01936 |
| FxJj18GS-5715 | 72.01493854 | 2.013943449 | 0.036425697 | 1.186833969 | 9.1681 | 1.59678 |
| FxJj18GS-5715 | 71.47892718 | 0.026696786 | 0.000106125 | 0.011103651 | 0.5566 | 0.01936 |
| FxJj18GS-5715 | 71.14374512 | 0.012579053 | 4.68342E-05 | 0.006784918 | 0.47509 | 0.00968 |
| GaJi4-1363 | 19.95103895 | 0.027318476 | 0.000160488 | 0.0135414 | 0.5852 | 0.0214 |
| GaJi4-1363 | 20.11067245 | 0.0594021 | 0.000479407 | 0.029735527 | 0.7921 | 0.03211 |
| GaJi4-1363 | 21.17476934 | 0.012865913 | 3.89315E-05 | 0.006794493 | 0.4995 | 0.0107 |
| GaJi4-1363 | 21.20853817 | 0.043529013 | 0.000245751 | 0.019355541 | 0.5854 | 0.02142 |
| GaJi4-1363 | 22.60264133 | 0.14245126 | 0.000848538 | 0.078806185 | 0.5852 | 0.0214 |
| GaJi4-1363 | 21.34764651 | 0.04322058 | 0.000187459 | 0.021879262 | 0.70654 | 0.02142 |
| GaJi4-1363 | 22.57526957 | 0.681451285 | 0.0026037 | 0.338413053 | 1.70557 | 0.12846 |
| GaJi4-1363 | 21.38646416 | 1.129574648 | 0.016755487 | 0.621660849 | 4.08201 | 0.35326 |
| GaJi4-1363 | 21.20685868 | 0.310792056 | 0.001782507 | 0.143244896 | 1.2916 | 0.08561 |
| GaJi4-1363 | 20.07644433 | 0.457029198 | 0.006237221 | 0.250640669 | 3.4965 | 0.26755 |
| GaJi4-1363 | 24.59163027 | 0.371753692 | 0.012580299 | 0.224225575 | 2.8758 | 0.25684 |
| GaJi4-1363 | 23.35700505 | 0.031722023 | 0.000138209 | 0.015015263 | 0.5852 | 0.0214 |
| GaJi4-1363 | 23.31476829 | 0.889220145 | 0.007693896 | 0.457741024 | 2.7546 | 0.23544 |
| GaJi4-1363 | 20.96718418 | 0.248140044 | 0.003492389 | 0.132116706 | 2.1694 | 0.21404 |
| GaJi4-1363 | 24.81122668 | 0.254709366 | 0.0025387 | 0.12642773 | 2.1192 | 0.12842 |
| GaJi4-1363 | 24.80065546 | 2.336914302 | 0.027107198 | 1.320239501 | 4.42467 | 0.71712 |
| GaJi4-3105 | 32.3347471 | 2.389067249 | 0.040062643 | 1.181869582 | 4.23073 | 0.90709 |
| GaJi4-3105 | 32.19964635 | 0.108949925 | 0.000616107 | 0.052868096 | 0.8032 | 0.04032 |
| GaJi4-3105 | 34.07708709 | 0.026693327 | 0.00023819 | 0.016398566 | 0.68557 | 0.02016 |
| GaJi4-3105 | 32.26098111 | 7.312161953 | 0.159561398 | 4.316376832 | 14.96438 | 2.76176 |
| GaJi4-3105 | 34.05618643 | 1.425133062 | 0.011135756 | 0.654164751 | 5.95478 | 0.9272 |
| GaJi4-3105 | 33.07690163 | 0.142822818 | 0.000506958 | 0.065741148 | 1.2048 | 0.08064 |
| GaJi4-3105 | 34.2566337 | 0.051468738 | 0.000185591 | 0.026281142 | 0.8032 | 0.04032 |
| GaJi4-3105 | 32.71369387 | 3.370355973 | 0.042275281 | 1.673406791 | 5.03407 | 1.02808 |
| GaJi4-3105 | 31.020231 | 0.068282955 | 0.00019473 | 0.034354149 | 0.8032 | 0.04032 |
| GaJi4-3105 | 30.91991582 | 5.255088737 | 0.067557495 | 2.834541112 | 12.47737 | 2.82207 |
| GaJi4-3105 | 34.63815327 | 0.169603364 | 0.001195529 | 0.079073968 | 1.2046 | 0.08062 |
| GaJi4-3105 | 34.51688522 | 3.156582978 | 0.04589103 | 1.869189414 | 6.47378 | 1.06834 |
| GaJi4-3105 | 34.74527155 | 0.058881451 | 0.000167168 | 0.028042757 | 0.8032 | 0.04032 |
| GaJi4-3105 | 30.45769629 | 3.642805139 | 0.079993416 | 2.21601482 | 5.60168 | 0.90705 |
| GaJi4-3105 | 30.20523625 | 0.034905772 | 0.000178151 | 0.016648098 | 0.68557 | 0.02016 |
| GaJi4-3105 | 30.00092465 | 0.068364265 | 0.000958753 | 0.034328827 | 0.8032 | 0.04032 |
| GaJi4-3105 | 29.37725368 | 0.938484579 | 0.057155118 | 0.580001246 | 4.79902 | 0.46369 |
| GaJi12-1269 | 53.73116943 | 0.346602355 | 0.005091371 | 0.1956444 | 2.21926 | 0.21003 |
| GaJi12-1269 | 54.0903802 | 0.101302783 | 0.000240161 | 0.050337931 | 1.39949 | 0.08399 |
| GaJi12-1269 | 57.62696745 | 0.086202415 | 0.000414336 | 0.041785215 | 1.10954 | 0.06299 |
| GaJi12-1269 | 58.37242834 | 0.681206601 | 0.017645569 | 0.366871802 | 4.43831 | 0.62999 |
| GaJi12-1269 | 58.1329041 | 1.931745366 | 0.036489984 | 1.042258109 | 6.19809 | 1.57511 |
| GaJi12-1269 | 57.04905987 | 0.048363888 | 0.000144483 | 0.023383403 | 0.8198 | 0.042 |
| GaJi12-1269 | 57.098128 | 2.739995938 | 0.048780329 | 1.12370516 | 8.58683 | 2.45711 |
| GaJi12-1269 | 52.87117426 | 1.28794527 | 0.021844084 | 0.612450271 | 6.29743 | 0.94508 |
| GaJi12-1269 | 55.20966409 | 0.23400121 | 0.002314595 | 0.118752313 | 1.80946 | 0.16801 |
| GaJi12-1269 | 52.53357492 | 0.191759457 | 0.002080865 | 0.106158089 | 1.92934 | 0.14704 |
| GaJi12-1269 | 52.50585202 | 0.049477086 | 0.000533163 | 0.02539316 | 0.8196 | 0.04198 |
| GaJi12-1269 | 52.55211246 | 1.341601821 | 0.029316608 | 0.780956862 | 5.01823 | 0.88204 |
| PrimeArch-1206 | 72.34069875 | 0.047444277 | 0.000238606 | 0.026693225 | 0.67704 | 0.01966 |
| PrimeArch-1206 | 73.24138787 | 0.045719782 | 0.000345504 | 0.025008944 | 0.7932 | 0.03932 |
| PrimeArch-1206 | 72.23105142 | 1.415664745 | 0.029319047 | 0.929830858 | 4.90355 | 0.84544 |
| PrimeArch-1206 | 71.0094302 | 0.579914225 | 0.007745411 | 0.210023839 | 3.22112 | 0.33428 |
| PrimeArch-1206 | 73.71619733 | 0.061806577 | 0.000296574 | 0.026040649 | 1.07381 | 0.05899 |
| PrimeArch-1206 | 70.83294034 | 0.039471525 | 0.000378072 | 0.018417451 | 0.67704 | 0.01966 |
| PrimeArch-1206 | 70.61683226 | 0.225373818 | 0.002390009 | 0.121408444 | 1.07384 | 0.059 |
| PrimeArch-1206 | 69.98372482 | 0.027915393 | 0.000139978 | 0.016598821 | 0.67704 | 0.01966 |
| PrimeArch-1206 | 73.71172997 | 0.385690836 | 0.003242992 | 0.200230268 | 2.94068 | 0.3146 |
| PrimeArch-1206 | 70.3833656 | 0.099115678 | 0.001743727 | 0.05947125 | 0.95748 | 0.03932 |
| PrimeArch-1206 | 73.86874351 | 0.137803299 | 0.000778832 | 0.063910126 | 1.75068 | 0.11797 |
| PrimeArch-1206 | 70.14901115 | 0.192163509 | 0.002366269 | 0.104923314 | 1.1898 | 0.07865 |
| PrimeArch-1206 | 73.44521216 | 0.983082956 | 0.01206698 | 0.487054704 | 4.13028 | 0.62917 |
| PrimeArch-1206 | 69.98271106 | 0.127076427 | 0.002121638 | 0.059909799 | 1.47024 | 0.09831 |
| PrimeArch-1206 | 73.72667867 | 0.179167433 | 0.002280181 | 0.075720982 | 2.14728 | 0.15729 |
| PrimeArch-1206 | 73.63084797 | 1.72401764 | 0.030078705 | 0.765174334 | 6.83877 | 1.45509 |
| PrimeArch-1206 | 73.7128221 | 3.654213961 | 0.102854789 | 1.767692816 | 15.66037 | 3.06737 |
| PrimeArch-1206 | 73.84913378 | 0.041498366 | 0.000123239 | 0.016301588 | 0.7932 | 0.03932 |
| PrimeArch-1206 | 73.65705873 | 0.043851573 | 0.000238085 | 0.021677298 | 0.7932 | 0.03932 |
| PrimeArch-1206 | 73.52082272 | 0.420923155 | 0.002355638 | 0.213931023 | 2.54405 | 0.31465 |
| PrimeArch-1206 | 69.23087728 | 0.083721239 | 0.000330505 | 0.039899582 | 1.35425 | 0.07868 |
| PrimeArch-1206 | 69.3498166 | 0.087126663 | 0.00120551 | 0.040816557 | 1.1898 | 0.07865 |
| PrimeArch-1206 | 68.58162275 | 2.925205908 | 0.027196804 | 1.531204542 | 5.99749 | 0.80617 |
| PrimeArch-1206 | 67.99697409 | 0.096991886 | 0.000891281 | 0.042301379 | 1.35445 | 0.0787 |
| PrimeArch-1206 | 67.30979943 | 0.135514664 | 0.001210545 | 0.062640053 | 1.63452 | 0.09831 |
| PrimeArch-1206 | 73.25515202 | 0.214621474 | 0.003322496 | 0.109818926 | 2.14728 | 0.19661 |
| PrimeArch-1206 | 68.95735469 | 1.058325486 | 0.008645811 | 0.502627391 | 4.17856 | 0.6882 |
| PrimeArch-1206 | 69.17454619 | 0.570281873 | 0.006587638 | 0.278420893 | 2.54388 | 0.27526 |
| PrimeArch-1206 | 67.5344845 | 0.250478716 | 0.001639935 | 0.124183717 | 1.75068 | 0.19661 |
| PrimeArch-1206 | 68.53800956 | 0.425664699 | 0.005155483 | 0.222723323 | 2.54388 | 0.27526 |
| PrimeArch-1206 | 69.11739002 | 0.767466675 | 0.017534171 | 0.389229511 | 3.73368 | 0.5112 |
| PrimeArch-1206 | 73.15318986 | 0.075860514 | 0.00040215 | 0.03920327 | 1.07364 | 0.05898 |
| PrimeArch-1206 | 67.62488717 | 0.152010464 | 0.001384195 | 0.082798553 | 1.1898 | 0.07865 |
| PrimeArch-1206 | 67.66549775 | 0.994237391 | 0.009993276 | 0.534808312 | 4.01412 | 0.64883 |
| PrimeArch-1206 | 67.40318678 | 2.462943441 | 0.023488585 | 1.351788557 | 6.72247 | 1.43533 |
| PrimeArch-1206 | 68.05084733 | 0.115908447 | 0.000676796 | 0.049569522 | 1.07364 | 0.05898 |
| PrimeArch-1206 | 69.17040465 | 0.051380929 | 0.000319679 | 0.024355261 | 0.95748 | 0.03932 |
| PrimeArch-1206 | 67.30957101 | 2.479975387 | 0.05779311 | 1.302732353 | 6.72227 | 1.98581 |
| PrimeArch-1206 | 68.8130137 | 3.586641831 | 0.049784227 | 1.857046035 | 7.74779 | 1.82851 |
| PrimeArch-1206 | 68.77667501 | 0.606472406 | 0.016066361 | 0.292603762 | 3.33708 | 0.55052 |
| PrimeArch-1206 | 67.45714785 | 0.081250711 | 0.000417117 | 0.032388729 | 1.1898 | 0.07865 |
| PrimeArch-1206 | 67.24057468 | 1.338803399 | 0.021443261 | 0.689132645 | 5.60072 | 1.08142 |
| PrimeArch-1206 | 67.42288691 | 0.107245913 | 0.000901035 | 0.045871548 | 1.47024 | 0.09831 |
| PrimeArch-1206 | 69.03770908 | 0.177145638 | 0.001122701 | 0.093318182 | 1.47024 | 0.09831 |
| PrimeArch-1206 | 67.37088445 | 1.3078522 | 0.022923715 | 0.612174783 | 5.4847 | 1.10106 |
| PrimeArch-1206 | 68.67855635 | 0.10583334 | 0.000440936 | 0.046656553 | 1.07364 | 0.05898 |
| PrimeArch-1206 | 66.66704163 | 1.068268884 | 0.005433878 | 0.542474242 | 3.45344 | 0.49162 |
| PrimeArch-1206 | 67.55007432 | 2.662467157 | 0.0342907 | 1.322692176 | 7.23523 | 1.69096 |
| PrimeArch-1206 | 68.48831965 | 1.355081442 | 0.021565811 | 0.727694763 | 6.27775 | 1.06178 |
| PrimeArch-1206 | 69.19078505 | 0.068122467 | 0.00080527 | 0.032225681 | 1.23792 | 0.05898 |
| PrimeArch-1206 | 68.65534266 | 0.262279002 | 0.001591547 | 0.121175156 | 2.42772 | 0.17695 |
| PrimeArch-1206 | 69.31806783 | 1.251768322 | 0.032580146 | 0.59043906 | 3.33722 | 0.51122 |
| PrimeArch-1206 | 69.21597618 | 1.047775532 | 0.01944212 | 0.482018348 | 3.78179 | 0.6095 |
| PrimeArch-1206 | 69.43795476 | 0.092211087 | 0.00071032 | 0.04350931 | 1.35408 | 0.07865 |
| PrimeArch-1206 | 67.19153869 | 0.407030739 | 0.004430004 | 0.170769526 | 2.54388 | 0.23594 |
| PrimeArch-1206 | 69.25546415 | 0.429025624 | 0.004137241 | 0.214743876 | 2.66024 | 0.25564 |
| PrimeArch-1206 | 68.45766797 | 3.87273705 | 0.047765613 | 1.987073285 | 6.55836 | 1.82878 |
| PrimeArch-1206 | 67.39660166 | 0.186765031 | 0.00105319 | 0.09171287 | 1.86721 | 0.13768 |
| PrimeArch-1206 | 68.79062924 | 0.022119596 | 8.308E-05 | 0.012179764 | 0.67704 | 0.01966 |
| PrimeArch-1206 | 68.71199002 | 0.297803502 | 0.003420017 | 0.12922994 | 2.31172 | 0.19662 |
| PrimeArch-1263 | 67.16033572 | 0.119386462 | 0.000371764 | 0.061602627 | 1.75652 | 0.11874 |
| PrimeArch-1263 | 70.27285206 | 0.104401776 | 0.001041814 | 0.05802301 | 1.47489 | 0.09892 |
| PrimeArch-1263 | 70.39730295 | 0.300888066 | 0.00157879 | 0.177492991 | 2.99866 | 0.29687 |
| PrimeArch-1263 | 68.56576022 | 0.625886134 | 0.010509555 | 0.275413872 | 4.02734 | 0.61358 |
| PrimeArch-1263 | 64.95514371 | 0.126888381 | 0.001775393 | 0.068623238 | 1.5916 | 0.11872 |
| PrimeArch-1263 | 64.97018427 | 0.041344471 | 0.000298323 | 0.021338497 | 0.7958 | 0.03958 |
| PrimeArch-1263 | 63.6810802 | 0.850668502 | 0.022849694 | 0.477578614 | 6.46306 | 0.83123 |

**Experimental Polygon Data**

**Red Polygons**

| **ID** | **Z_Mean** | **SArea** | **Volume** | **SArea2** | **Perimeter** | **Area** |
| --- | --- | --- | --- | --- | --- | --- |
| EHS-1-11 | -3.54984998 | 0.091281373 | 0.001947209 | 0.045500521 | 1.09509 | 0.06688 |
| EHS-1-11 | -3.13676134 | 0.347333685 | 0.014612941 | 0.177178297 | 2.17229 | 0.25697 |
| EHS-1-11 | 4.181912191 | 0.635285138 | 0.01426738 | 0.311246431 | 3.52995 | 0.58392 |
| EHS-1-11 | 4.480434589 | 1.553445818 | 0.026286751 | 0.815621557 | 5.5889 | 1.51624 |
| EHS-1-11 | 4.950700327 | 1.929339087 | 0.088202197 | 1.301620528 | 5.55243 | 1.83353 |
| EHS-1-11 | 3.755522682 | 8.960203651 | 0.424504552 | 3.853367436 | 18.47868 | 8.53787 |
| EHS-1-11 | 5.717586757 | 4.347757518 | 0.230091086 | 2.56398799 | 10.17102 | 4.0761 |
| EHS-1-11 | 5.422934592 | 2.020663208 | 0.043511881 | 1.080114914 | 7.34457 | 1.96024 |
| EHS-1-11 | 3.801583273 | 1.086599536 | 0.042036529 | 0.553930754 | 5.06443 | 0.9056 |
| EHS-1-11 | 6.923242698 | 0.168256086 | 0.002069718 | 0.081265483 | 1.57449 | 0.1642 |
| EHS-1-11 | 4.985033802 | 5.383312374 | 1.247452838 | 2.244838133 | 16.67513 | 4.42937 |
| EHS-1-11 | 7.178386454 | 7.954550292 | 0.325440032 | 4.343578811 | 15.52334 | 7.79786 |
| EHS-2-1 | 7.227416124 | 1.271204959 | 0.103846377 | 0.72776749 | 5.05607 | 0.87717 |
| EHS-2-1 | 15.89291684 | 0.559229398 | 0.011768631 | 0.315867872 | 3.08328 | 0.5451 |
| EHS-2-1 | 16.10657789 | 1.149140982 | 0.026306593 | 0.674670045 | 4.59609 | 1.10755 |
| EHS-2-1 | 16.42208757 | 0.279060252 | 0.002732555 | 0.139719225 | 2.47892 | 0.27503 |
| EHS-2-1 | 16.4637355 | 0.056312673 | 0.000111018 | 0.033541696 | 0.97215 | 0.05618 |
| EHS-2-1 | 15.79367327 | 0.089169001 | 0.002342588 | 0.046878339 | 1.06554 | 0.06691 |
| EHS-2-1 | 16.56830244 | 2.45112757 | 0.057514331 | 1.350685429 | 10.19163 | 2.42701 |
| EHS-2-1 | 16.45661066 | 4.028087632 | 0.179934955 | 2.146276823 | 10.82191 | 3.82481 |
| EHS-2-1 | 16.40906297 | 8.262639981 | 0.84529497 | 4.30580586 | 24.78465 | 7.80237 |
| EHS-2-1 | 16.39298338 | 0.771238748 | 0.027637635 | 0.421288351 | 4.91338 | 0.86666 |
| EHS-2-2 | 37.94988505 | 1.233852259 | 0.049838799 | 0.624285345 | 5.62071 | 1.16164 |
| EHS-2-2 | 38.62867476 | 18.1080598 | 0.750308963 | 10.75592712 | 34.53692 | 17.75569 |
| EHS-2-2 | 38.03612304 | 1.620511818 | 0.035237553 | 0.773133396 | 6.03893 | 1.60169 |
| EHS-2-2 | 37.18183069 | 0.706970075 | 0.01692683 | 0.36570349 | 4.16047 | 0.69113 |
| EHS-2-2 | 33.18689752 | 0.454159743 | 0.010890229 | 0.224527162 | 2.56466 | 0.41691 |
| EHS-2-3 | 3.472075331 | 0.09914569 | 0.000195017 | 0.05097225 | 1.53382 | 0.09871 |
| EHS-2-3 | -2.04335636 | 1.160062705 | 0.052425195 | 0.583262374 | 4.39968 | 1.02725 |
| EHS-2-3 | 3.227021559 | 0.499958725 | 0.003463256 | 0.251257159 | 3.13726 | 0.4964 |
| EHS-2-3 | 3.455081215 | 14.37323848 | 0.343232769 | 7.766085971 | 21.95204 | 14.18618 |
| EHS-2-3 | 3.175220696 | 0.31351679 | 0.001070303 | 0.158613731 | 2.47357 | 0.31248 |
| EHS-2-3 | 2.311867444 | 0.239779836 | 0.004635292 | 0.12847364 | 2.26868 | 0.22728 |
| EHS-2-3 | 3.222599425 | 8.543291877 | 0.369398801 | 4.98282766 | 18.01367 | 8.31643 |
| EHS-2-3 | 3.075235704 | 0.122212576 | 0.000785612 | 0.054892225 | 1.43354 | 0.1211 |
| EHS-2-3 | 3.040538174 | 0.144197055 | 0.000712965 | 0.07107251 | 1.5277 | 0.14355 |
| EHS-2-3 | 2.795534634 | 1.44363753 | 0.053095444 | 0.710113005 | 5.88617 | 1.41755 |
| EHS-2-4 | 10.69812367 | 8.481420376 | 0.238792877 | 4.521574446 | 14.36176 | 8.31923 |
| EHS-2-4 | 10.74781714 | 16.36533278 | 0.825975376 | 10.61107851 | 36.89361 | 16.10185 |
| EHS-2-4 | 10.09682527 | 0.65291247 | 0.026387113 | 0.374692331 | 3.74758 | 0.62535 |
| EHS-2-5 | -15.2127651 | 1.032629849 | 0.024315759 | 0.534085001 | 4.34719 | 1.01612 |
| EHS-2-5 | -14.8450253 | 0.42592052 | 0.003186661 | 0.216142753 | 2.68392 | 0.42409 |
| EHS-2-5 | -14.4998273 | 10.83268904 | 1.753199687 | 3.717087246 | 28.48701 | 10.64302 |
| EHS-2-5 | -15.2874356 | 2.658227693 | 0.044455037 | 1.554058742 | 9.46265 | 2.59697 |
| EHS-2-5 | -14.2579492 | 2.397251623 | 0.319939488 | 1.325468601 | 7.84228 | 2.10141 |
| EHS-2-5 | -13.0667936 | 11.59702238 | 0.562327551 | 6.480644633 | 17.185 | 11.02657 |
| EHS-2-5 | -14.484191 | 3.023529855 | 0.360633036 | 1.301972654 | 12.73671 | 2.88766 |
| EHS-2-5 | -15.3640834 | 0.417630449 | 0.003071189 | 0.181814205 | 2.57155 | 0.41527 |
| EHS-2-5 | -15.3249338 | 2.481563408 | 0.055451849 | 1.13680184 | 6.95677 | 2.46067 |
| EHS-2-5 | -15.1888178 | 27.13577316 | 1.633850961 | 16.12542831 | 36.15815 | 26.05715 |
| EHS-2-7 | -2.85748652 | 2.324618486 | 0.082584667 | 1.25899947 | 8.50831 | 2.11494 |
| EHS-2-7 | -2.03508316 | 0.510140387 | 0.006860004 | 0.300014793 | 2.97885 | 0.49665 |
| EHS-2-7 | -1.32604396 | 0.899970423 | 0.022728176 | 0.449352627 | 4.52635 | 0.79876 |
| EHS-2-7 | -1.91676374 | 0.855062901 | 0.044961531 | 0.473309941 | 3.87195 | 0.78819 |
| EHS-2-7 | -0.76467455 | 3.751568662 | 0.197489881 | 2.079561096 | 10.17663 | 3.56206 |
| EHS-2-7 | -2.21920435 | 0.653920188 | 0.016207843 | 0.341850639 | 3.40201 | 0.62493 |
| EHS-2-7 | 0.892898824 | 10.31263974 | 0.295979195 | 4.823688231 | 25.89201 | 10.10689 |
| EHS-2-7 | 0.750986657 | 0.288330029 | 0.004172348 | 0.13927131 | 2.11779 | 0.27488 |
| EHS-2-7 | -1.27354707 | 0.075788502 | 0.000733641 | 0.037537695 | 1.07424 | 0.07212 |
| EHS-2-7 | 0.457686916 | 2.042853884 | 0.124658539 | 1.166716965 | 8.01105 | 1.92298 |
| EHS-2-7 | -5.26800348 | 0.363999857 | 0.010345616 | 0.194584071 | 2.44309 | 0.32949 |
| EHS-2-7 | 0.839732146 | 1.903738531 | 0.053203315 | 0.959528692 | 6.1761 | 1.83437 |
| EHS-2-7 | -2.92723054 | 0.82553423 | 0.047208218 | 0.458010126 | 3.93188 | 0.68157 |
| EHS-2-7 | -3.33989714 | 0.107906823 | 0.002241443 | 0.054073855 | 1.18517 | 0.09011 |
| EHS-2-7 | -0.82973595 | 0.32280174 | 0.005383678 | 0.167075154 | 2.32488 | 0.30653 |
| EHS-2-7 | 1.143126462 | 6.778140534 | 0.369349074 | 4.291617259 | 13.07446 | 6.45817 |
| EHS-2-7 | -0.61780558 | 1.135725092 | 0.032193832 | 0.583043752 | 4.46397 | 1.0982 |
| EHS-2-7 | 0.071232819 | 0.119243198 | 0.000970276 | 0.064526893 | 1.31488 | 0.11569 |
| EHS-2-7 | 0.014452999 | 9.237212452 | 0.457202457 | 4.811929597 | 19.3565 | 8.70767 |
| EHS-2-7 | -0.7229273 | 0.989605416 | 0.040289786 | 0.509480564 | 4.10768 | 0.95101 |
| EHS-2-7 | -0.38172261 | 0.151986669 | 0.002259847 | 0.077444814 | 1.45375 | 0.14419 |
| EHS-2-7 | -0.21017149 | 6.652079003 | 0.183973285 | 4.025657132 | 12.58971 | 6.50219 |
| EHS-3-1 | 23.36455723 | 0.129675689 | 0.001184147 | 0.063800407 | 1.41038 | 0.12762 |
| EHS-3-1 | 23.39630219 | 4.717600393 | 0.126352301 | 2.500362212 | 13.33564 | 4.68299 |
| EHS-3-1 | 23.35254105 | 0.283256056 | 0.002023187 | 0.144334428 | 2.12704 | 0.28077 |
| EHS-3-1 | 23.55507179 | 0.54261029 | 0.004189166 | 0.227717882 | 2.85738 | 0.53882 |
| EHS-3-1 | 23.62484453 | 0.790301172 | 0.003033427 | 0.474993195 | 3.54939 | 0.7868 |
| EHS-3-1 | 23.58072623 | 12.52176169 | 0.158283525 | 6.863006394 | 22.5717 | 12.42959 |
| EHS-3-1 | 23.47309066 | 1.63763366 | 0.025797848 | 0.820572406 | 6.03316 | 1.61651 |
| EHS-3-1 | 23.11092607 | 0.563490971 | 0.010451555 | 0.298233475 | 2.83805 | 0.55299 |
| EHS-3-1 | 23.10119333 | 1.189638969 | 0.028646562 | 0.594803295 | 4.19391 | 1.16562 |
| EHS-3-3 | 30.504893 | 1.502621798 | 0.062481558 | 0.786788202 | 5.05297 | 1.42448 |
| EHS-3-3 | 31.06507541 | 16.88838548 | 0.926391471 | 11.54194102 | 30.92656 | 16.65995 |
| EHS-3-3 | 24.69651744 | 0.313377473 | 0.006495726 | 0.15683344 | 2.22199 | 0.29475 |
| EHS-3-3 | 25.56528287 | 0.068068201 | 0.000790247 | 0.038500443 | 1.07321 | 0.06141 |
| EHS-3-3 | 25.43361805 | 0.245886516 | 0.008722767 | 0.131940719 | 1.84053 | 0.20352 |
| EHS-3-3 | 24.30020368 | 3.690868528 | 0.494908323 | 1.691979353 | 10.4353 | 3.39383 |
| EHS-3-3 | 24.52296402 | 9.436187931 | 2.162100114 | 4.263920239 | 21.19661 | 7.93014 |
| EHS-4-1 | -19.1105589 | 7.611967212 | 0.199198174 | 3.670370427 | 14.26025 | 7.47285 |
| EHS-4-1 | -18.8710129 | 0.433119688 | 0.013030884 | 0.226892909 | 2.40355 | 0.38264 |
| EHS-4-1 | -18.6510339 | 49.63505506 | 3.23439839 | 28.25522933 | 71.70584 | 47.46129 |
| EHS-4-6 | 26.49772842 | 0.854579534 | 0.017854931 | 0.429543542 | 3.95759 | 0.84142 |
| EHS-4-6 | 26.5402154 | 0.693529011 | 0.013318945 | 0.377597873 | 3.63205 | 0.67956 |
| EHS-4-6 | 26.25259101 | 1.653157692 | 0.045621535 | 0.821615089 | 5.11379 | 1.587 |
| EHS-4-6 | 27.13134925 | 0.43819801 | 0.001921044 | 0.242258071 | 2.49004 | 0.43687 |
| EHS-4-6 | 27.09777059 | 18.34269863 | 0.398245357 | 9.02394313 | 27.45845 | 18.11763 |
| EHS-4-6 | 26.60729874 | 7.389609498 | 0.323820457 | 4.010844486 | 15.74661 | 7.11669 |
| EHS-4-6 | 25.27469541 | 2.272165957 | 0.077511922 | 1.295869261 | 6.82198 | 2.20861 |
| EHS-4-7 | -22.6476734 | 4.807072114 | 0.124184686 | 2.503227698 | 11.19812 | 4.76257 |
| EHS-4-7 | -22.5007701 | 0.963743573 | 0.008894331 | 0.459636479 | 4.07622 | 0.96045 |
| EHS-4-7 | -22.5103965 | 9.070875118 | 0.290872608 | 4.323903106 | 18.9173 | 8.95847 |
| EHS-4-7 | -23.4052658 | 0.612241306 | 0.01258901 | 0.316751598 | 3.0971 | 0.59273 |
| EHS-4-7 | -25.7994909 | 0.218810783 | 0.003878887 | 0.111877741 | 1.74792 | 0.20844 |
| EHS-4-7 | -25.8925849 | 0.087704092 | 0.000851922 | 0.040673776 | 1.17646 | 0.08469 |
| EHS-4-12 | 2.89405927 | 0.846436304 | 0.033095356 | 0.399207098 | 3.88274 | 0.79033 |
| EHS-4-12 | 7.095939721 | 1.420448845 | 0.076237927 | 0.672733334 | 4.78086 | 1.35616 |
| EHS-4-12 | 7.880928009 | 9.71501453 | 0.503848007 | 4.977051653 | 20.08594 | 9.43037 |
| EHS-4-12 | 10.22589847 | 12.75022996 | 0.498674746 | 5.251920629 | 24.43366 | 12.52789 |
| EHS-4-12 | 10.05666311 | 15.56452189 | 0.804811148 | 10.9300614 | 27.7811 | 15.24222 |
| EHS-4-12 | 8.940696335 | 1.477428273 | 0.036057619 | 0.7078163 | 5.26872 | 1.43691 |
| EHS-4-12 | 7.095384362 | 1.09963383 | 0.040764878 | 0.56195759 | 4.12511 | 0.99685 |
| EHS-4-44 | 8.623158708 | 0.053657295 | 0.000131146 | 0.033593614 | 0.95263 | 0.05282 |
| EHS-4-44 | 8.644646953 | 4.144271836 | 0.113564804 | 2.295543322 | 12.01846 | 3.99824 |
| EHS-4-44 | 8.780205048 | 1.460516992 | 0.039281238 | 0.761651691 | 5.45791 | 1.41805 |
| EHS-4-44 | 9.017325106 | 5.386932064 | 0.132760227 | 2.997739481 | 13.79082 | 5.25924 |
| EHS-4-44 | 11.0454844 | 0.351939348 | 0.006608979 | 0.208768242 | 2.36077 | 0.3327 |
| EHS-4-44 | 11.36289247 | 0.912271574 | 0.020359381 | 0.485084766 | 4.12847 | 0.87348 |
| EHS-4-44 | 11.72419702 | 0.460834674 | 0.008807769 | 0.262592584 | 2.68593 | 0.4437 |
| EHS-4-44 | 11.69720712 | 2.337974091 | 0.066687794 | 1.080470561 | 7.90455 | 2.24535 |
| EHS-4-44 | 9.057150587 | 1.908508406 | 0.08588358 | 1.091689072 | 7.83573 | 1.77121 |
| EHS-4-44 | 12.68010412 | 0.572331404 | 0.027735003 | 0.282136474 | 2.84686 | 0.50896 |
| EHS-4-44 | 12.11451244 | 0.10803894 | 0.000355145 | 0.055628859 | 1.2708 | 0.10696 |
| EHS-4-44 | 12.29934411 | 0.215447639 | 0.001551436 | 0.101084602 | 1.7969 | 0.21386 |
| EHS-4-44 | 13.16820776 | 3.91751095 | 0.147893583 | 2.408650392 | 10.07839 | 3.77037 |
| EHS-4-44 | 8.097021359 | 0.14414641 | 0.00211474 | 0.08700162 | 1.50568 | 0.13472 |
| EHS-4-44 | 13.66755363 | 1.059698389 | 0.023014275 | 0.468497686 | 5.41912 | 1.00627 |
| EHS-4-44 | 8.001194806 | 0.380658987 | 0.016209398 | 0.19094167 | 2.36074 | 0.28519 |
| EHS-4-44 | 14.13985147 | 0.225813347 | 0.002340174 | 0.104225673 | 1.99585 | 0.21987 |
| EHS-4-44 | 14.28776006 | 0.36825049 | 0.00420242 | 0.180818934 | 2.23086 | 0.36047 |
| EHS-4-44 | 13.58343211 | 0.477926774 | 0.017336211 | 0.218672788 | 2.79694 | 0.42783 |
| EHS-4-44 | 13.28965193 | 1.234880965 | 0.02591855 | 0.650624569 | 5.06666 | 1.17255 |
| EHS-4-44 | 14.19533258 | 23.71900966 | 1.876320655 | 16.0828222 | 45.47242 | 22.58029 |
| EHS-4-44 | 11.81376358 | 0.143387136 | 0.001977441 | 0.070799455 | 1.51757 | 0.12939 |
| EHS-4-44 | 12.3829612 | 1.27364675 | 0.047428279 | 0.649115569 | 4.49075 | 1.16131 |
| EHS-5-2 | 10.36130803 | 0.844521123 | 0.027254364 | 0.383381417 | 3.54765 | 0.80287 |
| EHS-5-2 | 10.50916287 | 0.585333916 | 0.008907922 | 0.246080951 | 3.17063 | 0.57257 |
| EHS-5-2 | 10.78145661 | 33.38870177 | 2.011144831 | 17.58286965 | 53.33264 | 32.58184 |
| EHS-5-2 | 10.14069974 | 0.328845151 | 0.005951104 | 0.164283671 | 2.24735 | 0.31446 |
| EHS-5-2 | 10.12675586 | 8.811906996 | 0.509647836 | 4.758677604 | 16.68236 | 8.56605 |
| EHS-5-2 | 8.304512678 | 3.81391033 | 0.149949825 | 2.082529802 | 8.49386 | 3.67767 |
| EHS-5-2 | 8.364424328 | 0.526435994 | 0.006814559 | 0.258422129 | 3.16424 | 0.51338 |
| EHS-5-2 | 6.609246668 | 0.262551831 | 0.003087983 | 0.12735104 | 1.99552 | 0.25527 |
| EHS-5-2 | 8.291786243 | 0.567517618 | 0.021588963 | 0.307178859 | 2.83395 | 0.52442 |
| EHS-5-2 | 8.042428645 | 1.83004971 | 0.073062845 | 1.105457484 | 5.84212 | 1.78974 |
| EHS-5-2 | 5.472871536 | 1.504249688 | 0.051002095 | 0.930099839 | 6.01162 | 1.4198 |
| EHS-5-2 | 4.298869455 | 1.309992613 | 0.049390924 | 0.66646593 | 4.50754 | 1.24318 |
| EHS-5-5 | 26.4443975 | 0.369727727 | 0.002612954 | 0.189078121 | 2.54143 | 0.36617 |
| EHS-5-5 | 26.86607669 | 0.259597476 | 0.001192586 | 0.143128048 | 2.13443 | 0.25831 |
| EHS-5-5 | 26.92298595 | 13.83621725 | 0.430765149 | 6.885493323 | 32.07645 | 13.63859 |
| EHS-5-5 | 26.499754 | 5.620329293 | 0.138261358 | 3.298066496 | 11.50248 | 5.50627 |
| EHS-5-9 | 13.82559697 | 0.174162641 | 0.000969232 | 0.094288816 | 1.74193 | 0.17208 |
| EHS-5-9 | 14.06636146 | 0.690549436 | 0.010944712 | 0.383824562 | 4.1091 | 0.68151 |
| EHS-5-9 | 14.16906446 | 0.250439857 | 0.00117506 | 0.121376564 | 1.96902 | 0.24871 |
| EHS-5-9 | 14.172361 | 0.05298778 | 0.000117077 | 0.02685571 | 1.05483 | 0.05286 |
| EHS-5-9 | 14.24052064 | 5.343020263 | 0.077108432 | 2.908295066 | 14.50157 | 5.28772 |
| EHS-5-9 | 14.22935464 | 4.004778557 | 0.082713065 | 2.096971645 | 12.34479 | 3.94163 |
| EHS-5-9 | 13.72678678 | 0.489696141 | 0.007219568 | 0.258513948 | 3.28264 | 0.47573 |
| EHS-5-9 | 13.83033002 | 0.495952225 | 0.00837025 | 0.283180619 | 3.45189 | 0.48777 |
| EHS-7-2 | 19.76197577 | 2.059036208 | 0.058050027 | 1.039208656 | 6.96321 | 1.97355 |
| EHS-7-2 | 22.86524579 | 1.689988164 | 0.037574374 | 0.919298749 | 7.02448 | 1.66029 |
| EHS-7-2 | 23.31200921 | 2.887717538 | 0.034134861 | 1.660775824 | 8.89837 | 2.87314 |
| EHS-7-2 | 23.28164053 | 0.156868203 | 0.001244241 | 0.080403503 | 1.52506 | 0.15404 |
| EHS-7-2 | 23.57101101 | 6.38907865 | 0.063357098 | 3.601232286 | 10.87727 | 6.35736 |
| EHS-7-2 | 23.42934079 | 0.326866055 | 0.00192727 | 0.13488866 | 2.26526 | 0.32524 |
| EHS-7-2 | 23.18024516 | 0.453267978 | 0.004910093 | 0.270093868 | 2.74343 | 0.44497 |
| EHS-7-2 | 23.26326247 | 0.226582546 | 0.002255218 | 0.105848054 | 1.89523 | 0.22254 |
| EHS-7-3 | 30.66294348 | 0.425085535 | 0.008591017 | 0.1817696 | 2.84985 | 0.41367 |
| EHS-7-3 | 30.84170836 | 0.278114501 | 0.004637771 | 0.138797042 | 2.10167 | 0.27217 |
| EHS-7-3 | 30.92318404 | 0.190851905 | 0.000820907 | 0.090351289 | 1.90526 | 0.18958 |
| EHS-7-3 | 31.19329356 | 0.098492204 | 0.000337478 | 0.049095015 | 1.21613 | 0.09797 |
| EHS-7-3 | 31.17791355 | 0.307325848 | 0.004007905 | 0.156718794 | 2.15976 | 0.30298 |
| EHS-7-3 | 30.93790947 | 1.10572003 | 0.023071599 | 0.457387255 | 5.84085 | 1.09341 |
| EHS-7-3 | 31.21706272 | 7.693072271 | 0.208182463 | 4.523070661 | 18.10844 | 7.60859 |
| EHS-7-3 | 30.99996661 | 1.005940357 | 0.017044902 | 0.526403439 | 4.64031 | 0.9861 |
| EHS-7-4 | 41.02704292 | 0.026054312 | 0.000100191 | 0.013244706 | 0.63065 | 0.02551 |
| EHS-7-4 | 41.06151896 | 0.031206455 | 0.000129952 | 0.015896855 | 0.69004 | 0.03064 |
| EHS-7-4 | 40.94184336 | 0.678422351 | 0.009714229 | 0.396340986 | 3.683 | 0.66956 |
| EHS-7-4 | 40.80219298 | 0.028151501 | 0.000302823 | 0.014676167 | 0.67198 | 0.02552 |
| EHS-7-4 | 40.67849499 | 1.352870279 | 0.023770369 | 0.773396419 | 6.43767 | 1.32278 |
| EHS-7-4 | 41.01779845 | 0.490334404 | 0.004789409 | 0.273179619 | 3.16742 | 0.48185 |
| EHS-7-4 | 41.33959384 | 3.272083936 | 0.041978895 | 1.612495953 | 12.58197 | 3.24002 |
| EHS-7-4 | 41.09751396 | 0.509072508 | 0.008111426 | 0.248502117 | 3.58224 | 0.49854 |
| EHS-7-4 | 40.52995482 | 0.411183614 | 0.004393399 | 0.215297796 | 3.15248 | 0.40212 |
| EHS-7-4 | 39.56853152 | 0.296453435 | 0.003883198 | 0.161589338 | 2.79001 | 0.28018 |
| EHS-7-4 | 40.16401239 | 0.038643924 | 0.000129419 | 0.020341806 | 0.82105 | 0.03828 |
| EHS-7-4 | 40.3943547 | 2.23372684 | 0.088105803 | 1.188795282 | 12.43642 | 2.16043 |
| EHS-7-4 | 40.33015095 | 1.106323168 | 0.012161757 | 0.635332375 | 4.98644 | 1.07535 |
| EHS-7-4 | 39.86543638 | 0.80164324 | 0.023127121 | 0.451817907 | 4.66597 | 0.76427 |
| EHS-7-4 | 40.19311721 | 1.628183517 | 0.034334032 | 0.945132976 | 7.15515 | 1.61522 |

**Blue polygons**

| EHS-1-11 | 4.5337134 | 0.505709966 | 0.008023314 | 0.283777509 | 3.48472 | 0.49262 |
| --- | --- | --- | --- | --- | --- | --- |
| EHS-1-11 | 4.514830328 | 1.621567353 | 0.07906623 | 0.838673028 | 7.44406 | 1.49599 |
| EHS-1-11 | 3.659123716 | 3.598010115 | 0.056762122 | 2.15308272 | 9.91386 | 3.55767 |
| EHS-1-11 | 5.841272469 | 0.134312777 | 0.001652836 | 0.078896811 | 1.95649 | 0.12772 |
| EHS-2-1 | 2.4816176 | 0.937166845 | 0.039906977 | 0.560982648 | 4.65641 | 0.78307 |
| EHS-2-1 | 4.428727193 | 2.34410834 | 0.164706781 | 1.442612519 | 7.27309 | 2.00218 |
| EHS-2-1 | 2.11422951 | 0.071674621 | 0.001243962 | 0.036450436 | 0.8448 | 0.03965 |
| EHS-2-1 | 1.57661475 | 0.372622898 | 0.010344826 | 0.187615409 | 2.85016 | 0.30728 |
| EHS-2-1 | 16.17925587 | 0.221912895 | 0.00241942 | 0.127103205 | 2.65104 | 0.21807 |
| EHS-2-1 | 16.38654927 | 0.140155214 | 0.00082315 | 0.083900846 | 2.0877 | 0.13873 |
| EHS-2-1 | 16.50724635 | 0.039744354 | 6.28289E-05 | 0.020965504 | 0.8448 | 0.03965 |
| EHS-2-1 | 15.61905121 | 0.022420039 | 0.000183053 | 0.011149411 | 0.5632 | 0.01982 |
| EHS-2-1 | 15.14706275 | 0.967526177 | 0.056986812 | 0.539223413 | 4.73869 | 0.87221 |
| EHS-2-1 | 16.33194847 | 0.145351185 | 0.000859953 | 0.072562452 | 2.36924 | 0.13876 |
| EHS-2-2 | 38.57041361 | 0.044413583 | 0.000170951 | 0.022381259 | 0.838 | 0.04389 |
| EHS-2-2 | 38.00125825 | 0.044288208 | 0.000123568 | 0.024362311 | 0.838 | 0.04389 |
| EHS-2-2 | 38.30239907 | 0.443093744 | 0.00470298 | 0.259173763 | 2.68736 | 0.43884 |
| EHS-2-3 | 3.148416668 | 3.794887885 | 0.039785987 | 2.06988669 | 12.73257 | 3.78599 |
| EHS-2-4 | 10.71942846 | 0.027876812 | 3.9374E-05 | 0.013988165 | 0.6668 | 0.02779 |
| EHS-2-4 | 10.72820941 | 0.028316862 | 0.000134167 | 0.012916126 | 0.80521 | 0.02781 |
| EHS-2-4 | 10.63802127 | 1.350330468 | 0.026895782 | 0.72624472 | 5.98525 | 1.32024 |
| EHS-2-4 | 10.72407129 | 0.028168183 | 9.70642E-05 | 0.013352043 | 0.80507 | 0.0278 |
| EHS-2-5 | -14.79801608 | 1.304131156 | 0.021923037 | 0.881665807 | 7.15401 | 1.29882 |
| EHS-2-5 | -15.28730255 | 1.539202266 | 0.065590775 | 0.839896059 | 7.55862 | 1.48448 |
| EHS-2-5 | -14.72356105 | 0.053060404 | 6.6166E-05 | 0.023644688 | 0.9208 | 0.05299 |
| EHS-2-5 | -14.9354205 | 0.159526932 | 0.000434517 | 0.082915719 | 2.03265 | 0.15905 |
| EHS-2-5 | -14.95913897 | 0.079897577 | 0.000222197 | 0.037521284 | 1.24655 | 0.07951 |
| EHS-2-5 | -15.4722944 | 3.361653395 | 0.053162715 | 1.618236074 | 8.59127 | 3.33975 |
| EHS-2-5 | -15.11020732 | 0.053568572 | 0.000226071 | 0.027891309 | 0.9208 | 0.05299 |
| EHS-2-5 | -15.42778149 | 0.027174438 | 0.000124354 | 0.013774832 | 0.78612 | 0.02651 |
| EHS-2-5 | -15.3476214 | 1.071215815 | 0.018052272 | 0.43210092 | 4.98649 | 1.06026 |
| EHS-2-5 | -15.22482073 | 0.161190742 | 0.001036798 | 0.088209383 | 1.842 | 0.15902 |
| EHS-2-7 | -2.748791103 | 0.019207259 | 0.000106486 | 0.010096198 | 0.64836 | 0.01803 |
| EHS-2-7 | -3.555829855 | 2.065277714 | 0.157886298 | 1.175709768 | 6.0115 | 1.0457 |
| EHS-2-7 | -2.139887718 | 1.744325027 | 0.076693339 | 0.982901171 | 6.16861 | 1.55036 |
| EHS-2-7 | -0.630691239 | 0.274784264 | 0.001988595 | 0.159710377 | 2.32471 | 0.27044 |
| EHS-2-7 | 0.644780239 | 0.036810132 | 0.000124175 | 0.01874505 | 0.91692 | 0.03606 |
| EHS-2-7 | 0.648370949 | 0.662589436 | 0.013434972 | 0.403248312 | 5.25184 | 0.64908 |
| EHS-2-7 | 0.943470944 | 0.890277193 | 0.016068616 | 0.472186238 | 5.56613 | 0.86539 |
| EHS-2-7 | -5.357502765 | 2.014801514 | 0.191839466 | 1.184832229 | 5.6774 | 1.56838 |
| EHS-2-7 | 0.02648369 | 0.698119031 | 0.014516081 | 0.340974894 | 4.76063 | 0.66706 |
| EHS-2-7 | -0.387272331 | 2.636086904 | 0.098468864 | 1.562946452 | 10.37286 | 2.45186 |
| EHS-2-7 | -0.333600331 | 0.86395025 | 0.031060231 | 0.442176299 | 3.84371 | 0.8112 |
| EHS-2-7 | -0.139367028 | 1.112809318 | 0.016685844 | 0.414717385 | 6.16835 | 1.08175 |
| EHS-2-7 | -0.149773341 | 0.128497111 | 0.000891265 | 0.064738506 | 1.40796 | 0.12622 |
| EHS-3-1 | 23.35918527 | 0.129097571 | 0.000742944 | 0.064824969 | 1.41055 | 0.12764 |
| EHS-3-1 | 23.63146903 | 0.10210857 | 9.96572E-05 | 0.054211471 | 1.3554 | 0.10206 |
| EHS-3-3 | 25.1682639 | 0.089575696 | 0.001119876 | 0.043351971 | 1.40119 | 0.08421 |
| EHS-3-3 | 25.11912935 | 0.370476208 | 0.012252066 | 0.176461486 | 3.33262 | 0.35786 |
| EHS-4-1 | -19.14990334 | 0.398485519 | 0.01020576 | 0.177384042 | 2.98707 | 0.38271 |
| EHS-4-1 | -19.09218835 | 0.075386764 | 0.00111593 | 0.036550663 | 0.99576 | 0.04253 |
| EHS-4-6 | 26.29170841 | 0.26915784 | 0.001674618 | 0.154872458 | 2.07412 | 0.26696 |
| EHS-4-6 | 26.32017076 | 0.02512754 | 0.000156741 | 0.013949647 | 0.75232 | 0.02428 |
| EHS-4-12 | 7.746527285 | 0.18948264 | 0.002497205 | 0.099602717 | 1.98738 | 0.1796 |
| EHS-4-12 | 7.726263303 | 0.154107157 | 0.001945183 | 0.076385895 | 1.83033 | 0.14372 |
| EHS-4-12 | 7.787777202 | 0.075524104 | 0.00076893 | 0.038039519 | 1.0722 | 0.07185 |
| EHS-4-44 | 8.699390916 | 0.119527944 | 0.000631154 | 0.067675205 | 1.6163 | 0.11881 |
| EHS-4-44 | 8.791673594 | 0.36529404 | 0.006182566 | 0.176188723 | 2.9247 | 0.35654 |
| EHS-4-44 | 8.632860801 | 0.148019406 | 0.001392014 | 0.058532285 | 1.48897 | 0.14265 |
| EHS-4-44 | 11.22061475 | 0.17531999 | 0.003641395 | 0.09499672 | 2.0523 | 0.16633 |
| EHS-4-44 | 11.87329775 | 0.049708472 | 0.000352428 | 0.023953895 | 0.8722 | 0.04755 |
| EHS-4-44 | 11.5212763 | 2.406428475 | 0.097332982 | 1.400259905 | 9.44324 | 2.2816 |
| EHS-4-44 | 12.26780342 | 0.119978272 | 0.000416326 | 0.065163306 | 1.61647 | 0.11884 |
| EHS-4-44 | 7.99588779 | 1.078162741 | 0.02026053 | 0.556429723 | 4.9772 | 1.04579 |
| EHS-4-44 | 13.32948486 | 0.071025516 | 0.001400884 | 0.035291519 | 0.8722 | 0.04755 |
| EHS-4-44 | 13.39509815 | 0.349000747 | 0.004964804 | 0.158044299 | 2.6162 | 0.33271 |
| EHS-5-2 | 10.71218403 | 0.916748742 | 0.01289274 | 0.47849437 | 4.70771 | 0.89907 |
| EHS-5-2 | 8.454000007 | 3.688129925 | 0.095273246 | 2.202397592 | 10.14541 | 3.59622 |
| EHS-5-5 | 26.51718057 | 0.579969464 | 0.008903259 | 0.288570749 | 3.86467 | 0.57318 |
| EHS-5-9 | 14.09855142 | 0.324833928 | 0.001022731 | 0.19022261 | 2.96331 | 0.32394 |
| EHS-5-9 | 14.19342222 | 0.091456744 | 0.000303507 | 0.051828877 | 1.45736 | 0.09111 |
| EHS-5-9 | 14.19610408 | 0.172432639 | 0.00041411 | 0.091239695 | 2.31096 | 0.17204 |
| EHS-7-3 | 30.96759794 | 0.098209244 | 0.000137049 | 0.052731712 | 1.38917 | 0.09798 |
| EHS-7-3 | 31.13075718 | 0.185938254 | 0.000687873 | 0.107416119 | 1.97937 | 0.18507 |
| EHS-7-4 | 40.96580073 | 0.026097523 | 6.23357E-05 | 0.012927822 | 0.74904 | 0.02551 |
| EHS-7-4 | 40.63585851 | 0.35300196 | 0.003633072 | 0.181794115 | 3.2823 | 0.34715 |
| EHS-7-4 | 41.3112756 | 0.015347339 | 1.9794E-05 | 0.008002338 | 0.54701 | 0.01531 |
| EHS-7-4 | 40.39992861 | 0.010337698 | 1.94638E-05 | 0.005186655 | 0.4044 | 0.01022 |
| EHS-7-4 | 40.38521715 | 0.010257689 | 1.16154E-05 | 0.005099664 | 0.404 | 0.0102 |
| EHS-7-4 | 40.36343948 | 0.260941973 | 0.005413641 | 0.140131991 | 2.91284 | 0.25523 |
| EHS-7-4 | 40.62024695 | 0.036336441 | 0.000102203 | 0.023259292 | 0.95141 | 0.03574 |
| EHS-7-4 | 40.20277913 | 0.35127044 | 0.004195691 | 0.165874755 | 3.08004 | 0.34709 |
| EHS-7-4 | 39.61232495 | 0.878716555 | 0.019564238 | 0.497545699 | 5.41136 | 0.85248 |
| EHS-7-4 | 40.37525818 | 0.020438498 | 1.03694E-05 | 0.006061932 | 0.68967 | 0.0204 |
| EHS-7-4 | 40.20409342 | 0.7791019 | 0.004474518 | 0.450266356 | 5.09062 | 0.77079 |
| EHS-7-4 | 40.02871643 | 0.285720484 | 0.003015565 | 0.17128176 | 2.85365 | 0.28078 |

**Natural Polygon Data**

**Red Polygons**

| **ID** | **Z_Mean** | **SArea** | **Volume** | **SArea2** | **Perimeter** | **Area** |
| --- | --- | --- | --- | --- | --- | --- |
| ND-2-1 | 67.43918959 | 0.063333961 | 0.000270388 | 0.031607743 | 1.00126 | 0.06266 |
| ND-2-1 | 67.88059362 | 2.628811133 | 0.01785948 | 1.593432496 | 8.21478 | 2.62468 |
| ND-2-1 | 67.84529964 | 0.324489861 | 0.000967073 | 0.15764969 | 2.32729 | 0.32378 |
| ND-2-1 | 67.85755211 | 0.081952328 | 0.000151865 | 0.040443696 | 1.10417 | 0.0818 |
| ND-2-1 | 67.85492816 | 3.497470402 | 0.021408029 | 1.559569276 | 11.08158 | 3.49362 |
| ND-2-1 | 65.6412295 | 1.300364605 | 0.021296684 | 0.585242312 | 5.84983 | 1.27904 |
| ND-2-1 | 65.46951037 | 0.578199684 | 0.004343495 | 0.26021113 | 3.54857 | 0.57582 |
| ND-2-1 | 67.74323988 | 2.828215703 | 0.06966603 | 1.599453328 | 9.98798 | 2.73866 |
| ND-2-1 | 68.48719065 | 3.824618552 | 0.0219596 | 2.105089416 | 9.48725 | 3.81865 |
| ND-2-1 | 68.45031078 | 0.121585742 | 0.000178325 | 0.06093243 | 1.44355 | 0.12147 |
| ND-2-1 | 68.44460372 | 0.182844698 | 0.000275531 | 0.094072441 | 1.7228 | 0.18275 |
| ND-2-1 | 68.43693491 | 0.668819551 | 0.001859756 | 0.345803436 | 3.68142 | 0.66842 |
| ND-2-1 | 68.33222886 | 2.426541199 | 0.045326062 | 1.285144484 | 9.47574 | 2.37607 |
| ND-2-1 | 67.70151613 | 0.139687544 | 0.000467302 | 0.065627029 | 1.38196 | 0.13924 |
| ND-2-1 | 68.13787771 | 7.447615465 | 0.243689745 | 3.765177168 | 21.72368 | 7.39194 |
| ND-2-1 | 67.26522531 | 0.113828998 | 0.00087812 | 0.061158462 | 1.28876 | 0.11186 |
| ND-2-1 | 64.35049085 | 0.363288424 | 0.004718162 | 0.1879657 | 2.6135 | 0.35513 |
| ND-2-2 | 64.48146737 | 0.058159086 | 0.00018044 | 0.029739463 | 0.94761 | 0.05778 |
| ND-2-2 | 65.53081185 | 3.414319333 | 0.010145384 | 1.869249387 | 9.40877 | 3.41173 |
| ND-2-2 | 65.48413467 | 0.0963567 | 0.000158892 | 0.055815622 | 1.22501 | 0.09626 |
| ND-2-2 | 64.10597209 | 4.76653063 | 0.327750016 | 2.019579364 | 16.40388 | 4.44442 |
| ND-2-2 | 65.3255873 | 0.087258158 | 0.000398582 | 0.042824401 | 1.14363 | 0.08664 |
| ND-2-2 | 64.24307351 | 0.679319847 | 0.011672188 | 0.385788259 | 4.03375 | 0.66425 |
| ND-2-2 | 62.41169645 | 0.224493795 | 0.001912969 | 0.10215374 | 2.0472 | 0.22059 |
| ND-2-2 | 64.44093182 | 0.25092366 | 0.00041203 | 0.136599328 | 2.00543 | 0.25082 |
| ND-2-2 | 64.90147974 | 0.310690748 | 0.005785031 | 0.171848188 | 3.13954 | 0.30484 |
| ND-2-2 | 64.60398045 | 0.764567094 | 0.022737323 | 0.392366661 | 4.98081 | 0.73644 |
| ND-2-2 | 63.86112225 | 0.096661294 | 0.000219969 | 0.042464062 | 1.22504 | 0.09629 |
| ND-2-2 | 63.98569235 | 1.069684555 | 0.007287212 | 0.642688838 | 4.91427 | 1.0624 |
| ND-2-2 | 64.4222579 | 7.892568519 | 0.095639044 | 5.632565719 | 29.27421 | 7.8706 |
| ND-2-2 | 63.8413137 | 0.153691554 | 0.000765459 | 0.073536772 | 1.57412 | 0.15134 |
| ND-2-2 | 64.31020883 | 0.18536403 | 0.000200191 | 0.090844613 | 1.90626 | 0.1853 |
| ND-2-2 | 64.31793093 | 4.605553217 | 0.017113476 | 2.495728951 | 17.52457 | 4.60151 |
| ND-2-2 | 63.48984654 | 0.50960778 | 0.002411313 | 0.280089417 | 3.44185 | 0.5086 |
| ND-2-2 | 64.2410575 | 0.231663049 | 0.000785832 | 0.118967406 | 1.89503 | 0.23104 |
| ND-2-2 | 63.50620616 | 0.191979803 | 0.000911893 | 0.099645155 | 1.72054 | 0.19145 |
| ND-2-2 | 63.53052602 | 0.160640278 | 0.000421031 | 0.071974792 | 1.71823 | 0.16045 |
| ND-2-3 | 42.97171583 | 7.612753406 | 0.399546199 | 4.172583449 | 25.1765 | 7.48687 |
| ND-2-3 | 44.97643921 | 0.146408377 | 0.000479386 | 0.0649409 | 1.51245 | 0.14604 |
| ND-2-3 | 45.06092139 | 2.966856816 | 0.057163724 | 1.28405147 | 12.8457 | 2.94317 |
| ND-2-3 | 44.77554734 | 13.22025227 | 3.398729857 | 9.105457516 | 39.48068 | 12.53827 |
| ND-2-4 | 36.19236465 | 1.8029277 | 0.020083561 | 0.977415484 | 6.12624 | 1.78623 |
| ND-2-4 | 36.93667169 | 0.223713356 | 0.0020471 | 0.116541675 | 2.17443 | 0.22135 |
| ND-2-4 | 36.96755217 | 0.067561972 | 0.000269452 | 0.035794713 | 1.08932 | 0.06708 |
| ND-2-4 | 37.37488273 | 0.026872357 | 3.03883E-05 | 0.013760207 | 0.6552 | 0.02683 |
| ND-2-4 | 37.17273771 | 0.332880924 | 0.001290584 | 0.152030232 | 2.2226 | 0.33208 |
| ND-2-4 | 34.6573806 | 0.276799832 | 0.000514586 | 0.138863285 | 1.99709 | 0.27653 |
| ND-2-4 | 37.36303873 | 3.319913311 | 0.027623568 | 1.847985409 | 9.45742 | 3.30049 |
| ND-2-4 | 34.76889193 | 0.871361064 | 0.005035552 | 0.491904815 | 4.04525 | 0.86798 |
| ND-2-4 | 37.00114237 | 0.01960907 | 4.58007E-05 | 0.010212368 | 0.59321 | 0.01938 |
| ND-2-4 | 36.02018237 | 0.439985849 | 0.008748839 | 0.239733443 | 2.61721 | 0.41152 |
| ND-2-4 | 37.17093878 | 2.681221404 | 0.026927022 | 1.269133283 | 9.25943 | 2.66305 |
| ND-2-4 | 36.02347084 | 0.180406232 | 0.002417574 | 0.087895171 | 1.69047 | 0.16996 |
| ND-2-4 | 37.13093477 | 5.129981275 | 0.174946839 | 2.448234402 | 17.01808 | 5.0976 |
| ND-2-4 | 36.23595335 | 0.453856981 | 0.008167831 | 0.253920134 | 3.52166 | 0.44721 |
| ND-2-4 | 34.88832347 | 0.033585474 | 3.15379E-05 | 0.0162589 | 0.76172 | 0.03354 |
| ND-2-4 | 34.82681661 | 0.999892497 | 0.014882227 | 0.539950196 | 4.61409 | 0.994 |
| ND-2-4 | 34.86272892 | 6.625946018 | 0.149885799 | 3.48420681 | 20.41547 | 6.45074 |
| ND-2-4 | 36.34033005 | 1.20376908 | 0.016094886 | 0.660605982 | 4.29195 | 1.18739 |
| ND-2-4 | 35.62169732 | 0.338953876 | 0.008581856 | 0.171967713 | 2.23716 | 0.29519 |
| ND-2-4 | 35.09238597 | 0.048970927 | 0.00084448 | 0.024784909 | 0.89634 | 0.04025 |
| ND-2-4 | 34.03995355 | 0.979326516 | 0.015441714 | 0.550296319 | 4.23139 | 0.96603 |
| ND-2-4 | 35.28101702 | 0.270569345 | 0.004277609 | 0.139251136 | 2.28944 | 0.25044 |
| ND-2-4 | 34.24882701 | 1.28184308 | 0.131807236 | 0.605693555 | 8.09977 | 1.20643 |
| ND-2-4 | 32.29857779 | 0.136823392 | 0.001499764 | 0.070209898 | 1.53382 | 0.1308 |
| ND-2-4 | 32.10750953 | 0.67996461 | 0.005344046 | 0.434676563 | 3.57255 | 0.67411 |
| ND-2-5 | 23.56681023 | 5.449934948 | 1.202076698 | 3.322785178 | 44.21788 | 5.41012 |
| ND-2-5 | 22.82938828 | 0.34119652 | 0.003840657 | 0.165391068 | 2.70608 | 0.33972 |
| ND-2-5 | 24.26150258 | 1.529268702 | 0.002908784 | 0.891236543 | 4.97266 | 1.52873 |
| ND-2-5 | 24.29331422 | 2.498335442 | 0.007022765 | 1.2750019 | 7.4395 | 2.49775 |
| ND-2-5 | 24.31628636 | 1.002166639 | 0.00101588 | 0.564405216 | 4.16928 | 1.002 |
| ND-2-5 | 24.35349213 | 4.569100961 | 0.012978703 | 2.124875273 | 10.37632 | 4.56749 |
| ND-2-5 | 24.38035004 | 2.425452651 | 0.008316436 | 1.449249644 | 6.30738 | 2.42402 |
| ND-2-5 | 24.28041323 | 0.463597765 | 0.00078473 | 0.203135285 | 3.54953 | 0.46348 |
| ND-2-5 | 24.29483569 | 0.476679575 | 0.000393904 | 0.239888726 | 2.72244 | 0.47664 |
| ND-2-5 | 24.31645406 | 0.363545289 | 0.000327637 | 0.19070465 | 2.57135 | 0.36342 |
| ND-2-5 | 24.25806231 | 1.822039027 | 0.003959997 | 0.896955451 | 6.16157 | 1.82133 |
| ND-2-5 | 24.24091939 | 0.094863447 | 0.000110972 | 0.05235469 | 1.23011 | 0.09479 |
| ND-2-5 | 24.27579981 | 1.708886853 | 0.004835805 | 1.017322035 | 6.1449 | 1.70836 |
| ND-2-5 | 24.23601714 | 0.450458849 | 0.00085157 | 0.225668459 | 2.62644 | 0.45027 |
| ND-2-5 | 24.15535679 | 0.183706783 | 0.000109458 | 0.094246704 | 1.7752 | 0.18369 |
| ND-2-5 | 24.15923571 | 0.118520839 | 0.000129519 | 0.056037611 | 1.35902 | 0.11848 |
| ND-2-5 | 24.16453914 | 0.086908809 | 5.27788E-05 | 0.043394561 | 1.24812 | 0.08689 |
| ND-2-5 | 24.16306774 | 0.13442914 | 0.000245034 | 0.071859148 | 1.50333 | 0.1343 |
| ND-2-5 | 24.13249171 | 0.33187493 | 0.000305248 | 0.162117233 | 2.24275 | 0.33182 |
| ND-2-5 | 24.12173618 | 0.237025381 | 0.000172876 | 0.099364439 | 1.92212 | 0.237 |
| ND-2-5 | 23.17046953 | 15.99193245 | 1.668109754 | 7.715193239 | 68.82921 | 18.62836 |
| ND-2-5 | 24.01154573 | 0.765991709 | 0.002103293 | 0.541208923 | 4.23022 | 0.76563 |
| ND-2-5 | 23.96961842 | 0.254430707 | 0.000567409 | 0.109887993 | 2.65194 | 0.25415 |
| ND-2-5 | 24.01882308 | 2.824241213 | 0.011553414 | 1.420303744 | 8.43536 | 2.8229 |
| ND-2-5 | 22.51613712 | 2.145924659 | 0.186691185 | 0.960094201 | 7.28654 | 1.69699 |
| ND-2-5 | 23.94788203 | 1.742809952 | 0.007557989 | 0.989514261 | 6.32393 | 1.74156 |
| ND-2-5 | 22.01026855 | 1.089503781 | 0.030997533 | 0.489168477 | 7.39152 | 1.40093 |
| ND-2-5 | 23.88940347 | 5.836034883 | 0.058074366 | 2.716007487 | 13.04134 | 5.83092 |
| ND-2-5 | 23.91122964 | 11.79137793 | 1.255862081 | 6.801782188 | 68.80091 | 11.7562 |
| ND-2-5 | 21.63254194 | 0.290501139 | 0.00373843 | 0.137276444 | 4.63206 | 0.42392 |
| ND-2-5 | 23.65849546 | 6.152779039 | 0.045924054 | 3.185611122 | 14.04209 | 6.13833 |
| ND-2-5 | 23.84232633 | 5.712594885 | 0.017691104 | 2.986631147 | 11.84654 | 5.70982 |
| ND-2-5 | 22.46344389 | 0.260979796 | 0.000709363 | 0.13250004 | 2.04985 | 0.26076 |
| ND-2-5 | 23.34108389 | 0.071254686 | 0.000133028 | 0.038010416 | 1.10259 | 0.0711 |
| ND-2-5 | 23.7115975 | 2.994606871 | 0.014066558 | 1.693455887 | 8.28856 | 2.99079 |
| ND-2-5 | 23.62760796 | 0.223267039 | 0.00019707 | 0.125948566 | 2.09287 | 0.22317 |
| ND-2-5 | 23.26731828 | 9.343649046 | 1.497749401 | 5.87821145 | 54.26457 | 9.28059 |
| ND-2-5 | 22.92726035 | 0.202534242 | 0.010754559 | 0.10205305 | 0.97953 | 0.06188 |
| ND-4-1 | 24.09789621 | 0.30134676 | 0.001392728 | 0.159423316 | 2.26061 | 0.30086 |
| ND-4-1 | 24.65179279 | 0.229689796 | 0.001606405 | 0.121551877 | 1.8334 | 0.22771 |
| ND-4-1 | 25.07436776 | 0.624685547 | 0.013036245 | 0.327522151 | 3.54769 | 0.61628 |
| ND-4-1 | 25.73889 | 0.657533559 | 0.005356349 | 0.355186528 | 3.46998 | 0.65127 |
| ND-4-1 | 21.5577067 | 0.793679964 | 0.009792828 | 0.409955874 | 4.52905 | 0.77971 |
| ND-4-1 | 21.61111082 | 0.070411265 | 0.000391223 | 0.03700308 | 1.18005 | 0.06897 |
| ND-4-1 | 27.34543243 | 9.092049087 | 0.102223478 | 5.911900716 | 19.14545 | 9.07966 |
| ND-4-1 | 21.76919983 | 0.314861356 | 0.002608774 | 0.160552337 | 2.51416 | 0.3115 |
| ND-4-2 | 90.1372068 | 0.049375888 | 0.000265766 | 0.025783507 | 0.91177 | 0.04861 |
| ND-4-2 | 90.19749353 | 4.531764707 | 0.298573263 | 1.917774097 | 15.70573 | 4.43563 |
| ND-4-2 | 91.26832765 | 2.872374732 | 0.023242959 | 1.52419074 | 12.02063 | 2.86 |
| ND-4-2 | 91.28958453 | 0.773666571 | 0.003801393 | 0.393130538 | 4.56792 | 0.77221 |
| ND-4-2 | 91.00514106 | 0.695171508 | 0.007827837 | 0.335000426 | 4.33702 | 0.69047 |
| ND-4-2 | 90.65933859 | 1.113657156 | 0.055308313 | 0.581363543 | 5.36202 | 1.0731 |
| ND-4-2 | 90.88270521 | 1.221002879 | 0.004687774 | 0.672634844 | 6.42498 | 1.21808 |
| ND-4-2 | 91.90511512 | 5.195514282 | 0.02652668 | 3.153113179 | 17.88635 | 5.19139 |
| ND-4-2 | 91.5258328 | 0.473145493 | 0.000540387 | 0.229590171 | 3.24638 | 0.47306 |
| ND-4-2 | 91.61977935 | 1.064205189 | 0.001216997 | 0.594003831 | 4.48484 | 1.06409 |
| ND-4-2 | 91.8579619 | 0.227288577 | 0.0005439 | 0.108401028 | 1.96104 | 0.22688 |
| ND-4-2 | 92.01934454 | 6.002929833 | 0.044471523 | 3.37606952 | 17.56486 | 5.99969 |
| ND-4-2 | 91.63718151 | 0.175901843 | 0.000944333 | 0.082331333 | 1.65937 | 0.17465 |
| ND-4-2 | 91.78574955 | 3.12580234 | 0.022274358 | 2.180852476 | 11.51557 | 3.12204 |
| ND-4-2 | 91.72689626 | 2.910606263 | 0.079851901 | 1.477459453 | 13.01151 | 2.90202 |
| ND-4-2 | 91.4999292 | 0.945907298 | 0.006784427 | 0.587424258 | 5.22753 | 0.9421 |
| ND-4-2 | 88.35183824 | 0.856977957 | 0.006190829 | 0.524363993 | 4.31693 | 0.8534 |
| ND-4-3 | 81.67975665 | 0.341365677 | 0.005633518 | 0.157108712 | 2.69751 | 0.33207 |
| ND-4-3 | 81.6510205 | 1.956913896 | 0.030299357 | 1.072272664 | 6.52614 | 1.90985 |
| ND-4-3 | 82.19016166 | 1.420111142 | 0.017176056 | 0.839398045 | 6.15229 | 1.3867 |
| ND-4-3 | 82.39688604 | 2.437415278 | 0.034336279 | 1.160735387 | 8.46778 | 2.42389 |
| ND-4-3 | 82.50351401 | 0.808263924 | 0.00593607 | 0.464854614 | 3.91283 | 0.80247 |
| ND-4-3 | 82.44837662 | 10.19058797 | 0.538663967 | 5.970387013 | 27.76856 | 9.99283 |
| ND-4-3 | 82.10488089 | 0.250331162 | 0.001253599 | 0.125593726 | 1.99505 | 0.24906 |
| ND-4-3 | 82.0560383 | 0.682530866 | 0.005737612 | 0.311522062 | 3.82961 | 0.67147 |
| ND-4-3 | 84.3545175 | 0.273272669 | 0.00312162 | 0.136267314 | 2.46042 | 0.26031 |
| ND-4-3 | 82.09388035 | 4.082157052 | 0.121790251 | 1.994391786 | 11.89663 | 4.00296 |
| ND-4-3 | 85.03188096 | 6.744685704 | 0.158916046 | 3.649923718 | 22.19219 | 6.71408 |
| ND-4-3 | 82.27001462 | 0.243703147 | 0.004200221 | 0.132978179 | 2.3726 | 0.2348 |
| ND-4-3 | 84.83641331 | 8.949520779 | 0.521738113 | 5.206951456 | 29.56163 | 8.81279 |
| ND-4-3 | 84.92414979 | 0.123436766 | 0.001729382 | 0.055910817 | 1.45323 | 0.11936 |
| ND-4-3 | 84.95207032 | 0.072929982 | 0.000308428 | 0.039101083 | 1.23785 | 0.07266 |
| ND-4-3 | 83.78999056 | 0.2862845 | 0.010027508 | 0.13114052 | 2.60984 | 0.26592 |
| ND-4-3 | 83.16942597 | 1.180353097 | 0.058939438 | 0.614944654 | 5.52544 | 1.11079 |
| ND-4-3 | 85.01695554 | 3.899869809 | 0.104161617 | 2.792234133 | 12.43072 | 3.8819 |
| ND-4-3 | 84.33769577 | 0.348195282 | 0.004891466 | 0.17771496 | 2.72461 | 0.33334 |
| ND-4-3 | 84.82995245 | 0.145563648 | 0.000339847 | 0.080517633 | 1.73074 | 0.14528 |
| ND-4-3 | 83.85804516 | 0.976628101 | 0.014246538 | 0.573315787 | 5.14992 | 0.95815 |
| ND-4-3 | 84.79539821 | 0.244944351 | 0.001668823 | 0.128660583 | 2.21634 | 0.24387 |
| ND-4-3 | 83.53816955 | 5.243587083 | 0.07376075 | 2.17820435 | 14.63221 | 5.21677 |
| ND-4-3 | 81.15073511 | 1.447593292 | 0.033837291 | 0.812915715 | 6.28693 | 1.38746 |
| ND-4-8 | 27.58989577 | 0.357483728 | 0.001675441 | 0.19422677 | 3.12604 | 0.35621 |
| ND-4-8 | 27.58178212 | 0.540517156 | 0.002615733 | 0.287979758 | 3.60134 | 0.53766 |
| ND-4-8 | 26.94813301 | 0.014265679 | 1.48849E-05 | 0.007388649 | 0.49 | 0.01423 |
| ND-4-8 | 27.31193076 | 0.418785331 | 0.01300862 | 0.255340103 | 3.19831 | 0.40173 |
| ND-4-8 | 26.95643793 | 3.389360353 | 0.023816138 | 1.455263421 | 13.51217 | 3.37686 |
| ND-4-8 | 27.15387717 | 0.274345739 | 0.000687406 | 0.139432925 | 2.33118 | 0.27406 |
| ND-4-8 | 27.49770618 | 0.025378153 | 4.03897E-05 | 0.013380721 | 0.63611 | 0.02529 |
| ND-4-8 | 26.51980307 | 0.301885587 | 0.002008707 | 0.171069703 | 2.36533 | 0.29791 |
| ND-4-8 | 27.56336159 | 2.748323883 | 0.010400172 | 1.707714973 | 9.72916 | 2.74277 |
| ND-4-8 | 26.9743125 | 0.12781461 | 0.00046689 | 0.079667928 | 1.37223 | 0.12646 |
| ND-4-8 | 26.95768923 | 0.034078416 | 7.48952E-05 | 0.017907483 | 0.85238 | 0.03369 |
| ND-4-8 | 27.32226444 | 0.052718321 | 5.03888E-05 | 0.0352104 | 0.95012 | 0.05268 |
| ND-4-8 | 28.59829679 | 0.54357131 | 0.00200096 | 0.289987759 | 3.4441 | 0.54154 |
| ND-4-8 | 27.68870757 | 3.944522103 | 0.039968304 | 2.504139472 | 14.11827 | 3.91849 |
| ND-4-8 | 27.44040129 | 4.02844654 | 0.035022667 | 2.056941184 | 12.80401 | 4.00931 |
| ND-4-11 | 85.25257831 | 0.295866859 | 0.001229254 | 0.158575301 | 2.2388 | 0.29508 |
| ND-4-11 | 86.03056474 | 3.823772461 | 0.029411109 | 1.973681489 | 9.32461 | 3.81269 |
| ND-4-11 | 84.75314899 | 3.057225301 | 0.022210419 | 1.925825193 | 7.8339 | 3.04639 |
| ND-4-11 | 84.59922876 | 0.281015879 | 0.000732139 | 0.122683105 | 2.24801 | 0.28071 |
| ND-4-11 | 85.10690902 | 7.156119913 | 0.093395996 | 4.151199013 | 20.47046 | 7.14185 |
| ND-4-11 | 85.06268463 | 2.624048557 | 0.015036211 | 1.592241927 | 7.23133 | 2.6196 |
| ND-4-11 | 81.89054744 | 0.491767463 | 0.005414273 | 0.249948661 | 2.87269 | 0.47693 |
| ND-4-11 | 81.91175447 | 0.079224731 | 0.000875525 | 0.040116956 | 1.08626 | 0.07375 |
| ND-4-11 | 81.93723001 | 0.072924464 | 0.000830089 | 0.03636892 | 1.10109 | 0.06762 |
| ND-4-11 | 81.01009356 | 0.056003851 | 0.000241154 | 0.027229941 | 0.97263 | 0.05533 |
| ND-4-11 | 82.00083542 | 0.053751698 | 0.000549289 | 0.031248798 | 0.98732 | 0.04919 |
| ND-4-11 | 81.20685579 | 1.56003053 | 0.007341756 | 0.792824613 | 4.99368 | 1.55778 |
| ND-4-11 | 80.48633211 | 0.316595988 | 0.002839303 | 0.148995514 | 2.26574 | 0.31301 |
| ND-4-11 | 81.62441886 | 1.337359209 | 0.017577376 | 0.76677037 | 4.56067 | 1.31036 |
| ND-4-11 | 80.52363259 | 0.19013229 | 0.001330087 | 0.095230157 | 1.96412 | 0.18646 |

**Blue Polygons**

| ND-2-1 | 64.82396369 | 0.89028259 | 0.01900499 | 0.502111917 | 3.87658 | 0.8304 |
| --- | --- | --- | --- | --- | --- | --- |
| ND-2-1 | 66.0879792 | 0.032602716 | 0.000192304 | 0.016518968 | 0.708 | 0.03133 |
| ND-2-1 | 66.0920906 | 0.032279277 | 0.00016927 | 0.016182276 | 0.708 | 0.03133 |
| ND-2-1 | 65.02651187 | 0.077520694 | 0.001347335 | 0.040988584 | 1.0014 | 0.06268 |
| ND-2-1 | 65.18495972 | 5.024700861 | 0.376063633 | 2.304356885 | 12.38026 | 4.01045 |
| ND-2-1 | 64.69891516 | 1.471151733 | 0.086972474 | 0.78949845 | 5.49992 | 1.17496 |
| ND-2-1 | 67.01385712 | 2.370973361 | 0.178442612 | 1.346161547 | 7.25213 | 1.786 |
| ND-2-1 | 67.67624724 | 0.952274745 | 0.010440729 | 0.468732838 | 4.54166 | 0.9401 |
| ND-2-1 | 67.18414678 | 1.113323355 | 0.028648387 | 0.569876436 | 4.79175 | 1.08089 |
| ND-2-1 | 66.4993665 | 0.713114697 | 0.032878046 | 0.404851213 | 3.79052 | 0.61102 |
| ND-2-1 | 64.33713686 | 0.016523832 | 8.20169E-05 | 0.007405126 | 0.60432 | 0.01566 |
| ND-2-1 | 64.11817565 | 1.678131507 | 0.076455103 | 0.900357034 | 7.10553 | 1.50405 |
| ND-2-2 | 59.79341714 | 1.980851878 | 0.118358909 | 1.103393045 | 9.21102 | 1.81936 |
| ND-2-2 | 61.41609137 | 0.466665411 | 0.009335867 | 0.27040767 | 2.80894 | 0.43324 |
| ND-2-2 | 60.57668329 | 1.06647499 | 0.036553004 | 0.618111893 | 4.54121 | 0.97231 |
| ND-2-2 | 61.57535442 | 1.697593668 | 0.03660529 | 0.943678058 | 6.84275 | 1.59805 |
| ND-2-2 | 61.96364865 | 0.675196832 | 0.010237101 | 0.402309726 | 4.06752 | 0.65461 |
| ND-2-2 | 63.3235888 | 0.970153039 | 0.034592791 | 0.539409927 | 5.17738 | 0.88567 |
| ND-2-2 | 63.19211574 | 0.593616461 | 0.013398474 | 0.320564862 | 3.59383 | 0.54872 |
| ND-2-2 | 62.98433553 | 1.097906244 | 0.01491309 | 0.665632838 | 5.42138 | 1.06857 |
| ND-2-2 | 63.16498734 | 0.021827097 | 0.000178253 | 0.010929928 | 0.5552 | 0.01927 |
| ND-2-2 | 63.95941356 | 0.676662547 | 0.004528467 | 0.465431658 | 4.78516 | 0.67387 |
| ND-2-2 | 64.02706717 | 0.009692098 | 1.41419E-05 | 0.00430593 | 0.47355 | 0.00962 |
| ND-2-2 | 64.04463702 | 0.019353016 | 4.07613E-05 | 0.009037104 | 0.67001 | 0.01926 |
| ND-2-2 | 64.06252593 | 0.009669965 | 1.35593E-05 | 0.005348641 | 0.47372 | 0.00963 |
| ND-2-2 | 63.86777295 | 0.030027322 | 0.000114059 | 0.015104527 | 0.75132 | 0.02889 |
| ND-2-2 | 64.07679805 | 0.01932985 | 2.63545E-05 | 0.010317515 | 0.5552 | 0.01927 |
| ND-2-2 | 63.81488349 | 0.010785448 | 6.20418E-05 | 0.005606163 | 0.47372 | 0.00963 |
| ND-2-2 | 64.07570104 | 0.164092755 | 0.00041028 | 0.083536921 | 2.25371 | 0.16365 |
| ND-2-2 | 63.66439024 | 1.08330622 | 0.02896128 | 0.581199846 | 7.31616 | 1.03007 |
| ND-2-2 | 63.73479903 | 0.020399124 | 0.000113926 | 0.009970529 | 0.555 | 0.01925 |
| ND-2-2 | 63.61069889 | 0.540721849 | 0.009711499 | 0.274667718 | 3.56012 | 0.51987 |
| ND-2-2 | 62.69656853 | 1.355411511 | 0.0197239 | 0.747547581 | 5.76626 | 1.33812 |
| ND-2-3 | 42.73632761 | 0.913757472 | 0.00758065 | 0.525656938 | 4.3266 | 0.90637 |
| ND-2-3 | 42.67151459 | 1.582602135 | 0.035099879 | 0.957892718 | 7.87038 | 1.57108 |
| ND-2-3 | 44.94576335 | 0.713386693 | 0.008808743 | 0.390175839 | 3.95962 | 0.69491 |
| ND-2-4 | 35.25293992 | 0.419101624 | 0.002937531 | 0.235611464 | 2.98834 | 0.41589 |
| ND-2-4 | 35.62568795 | 3.908790665 | 0.146798691 | 2.351623207 | 17.19495 | 3.70292 |
| ND-2-4 | 36.897383 | 0.229975138 | 0.000932523 | 0.134991369 | 2.19745 | 0.22814 |
| ND-2-4 | 37.13100485 | 0.392071067 | 0.003339992 | 0.237154952 | 2.98814 | 0.38904 |
| ND-2-4 | 36.99552548 | 1.284717981 | 0.026752032 | 0.624148189 | 5.97649 | 1.26105 |
| ND-2-4 | 34.63915206 | 3.441024115 | 0.149941403 | 1.712945694 | 14.66984 | 3.28703 |
| ND-2-4 | 34.60015821 | 1.005008877 | 0.040620415 | 0.535723699 | 6.43976 | 0.91229 |
| ND-2-4 | 36.03147559 | 1.210586888 | 0.020427592 | 0.609014748 | 5.22545 | 1.16736 |
| ND-2-4 | 33.91992763 | 0.681348759 | 0.025973682 | 0.331666733 | 3.45181 | 0.63064 |
| ND-2-4 | 36.17239612 | 0.054312706 | 0.000250177 | 0.02769175 | 0.9828 | 0.05366 |
| ND-2-4 | 34.82041863 | 0.067604559 | 0.000248567 | 0.030938597 | 1.21445 | 0.06708 |
| ND-2-4 | 33.70947633 | 0.076632374 | 0.002401406 | 0.035442105 | 1.35014 | 0.06708 |
| ND-2-4 | 33.44846541 | 0.017101266 | 0.000204263 | 0.007919022 | 0.55925 | 0.01342 |
| ND-2-4 | 35.49663339 | 1.979185986 | 0.101421033 | 1.297160356 | 10.46711 | 1.83804 |
| ND-2-4 | 35.33733111 | 0.01423026 | 7.84178E-05 | 0.006536968 | 0.55942 | 0.01342 |
| ND-2-4 | 33.082114 | 0.109526709 | 0.003986194 | 0.060409951 | 1.81372 | 0.09394 |
| ND-2-4 | 35.12329491 | 0.438038295 | 0.004842956 | 0.319720474 | 4.01069 | 0.42929 |
| ND-2-4 | 31.94251744 | 5.672200999 | 0.849506856 | 2.931592865 | 26.91764 | 5.541 |
| ND-2-5 | 22.81084051 | 2.366664968 | 0.035278276 | 1.125818091 | 7.81609 | 2.34628 |
| ND-2-5 | 24.23313687 | 0.64016179 | 0.002004398 | 0.461284776 | 4.58768 | 0.63989 |
| ND-2-5 | 24.31893767 | 0.047419539 | 1.56584E-05 | 0.024350312 | 0.871 | 0.04742 |
| ND-2-5 | 24.2736444 | 2.420401698 | 0.013115649 | 1.082497234 | 7.32767 | 2.41737 |
| ND-2-5 | 24.24393366 | 1.114503854 | 0.004038609 | 0.682068444 | 4.84256 | 1.11387 |
| ND-2-5 | 24.21570214 | 0.331925251 | 0.000413043 | 0.182339722 | 2.53801 | 0.33183 |
| ND-2-5 | 24.16175523 | 0.095010787 | 0.000194738 | 0.050228225 | 1.3062 | 0.09479 |
| ND-2-5 | 23.95142233 | 0.569243178 | 0.001715322 | 0.302410802 | 3.84427 | 0.56888 |
| ND-2-5 | 23.93275969 | 3.058894733 | 0.014838408 | 1.444848333 | 14.78266 | 3.05739 |
| ND-2-5 | 22.76920658 | 0.831108868 | 0.006227579 | 0.488258596 | 4.40719 | 0.82952 |
| ND-2-5 | 23.10736471 | 1.634556191 | 0.055100598 | 0.87602491 | 5.76616 | 1.51677 |
| ND-2-5 | 22.48091904 | 0.807830784 | 0.003618511 | 0.445133756 | 4.71507 | 0.80576 |
| ND-2-5 | 23.29530759 | 1.263434501 | 0.013522821 | 0.541378505 | 5.71362 | 1.2561 |
| ND-4-1 | 24.1168625 | 1.111149318 | 0.004002633 | 0.558974504 | 6.02525 | 1.10928 |
| ND-4-1 | 24.56988486 | 2.413200492 | 0.117617933 | 1.153541761 | 7.02819 | 2.12459 |
| ND-4-1 | 24.28316323 | 0.848614358 | 0.006611858 | 0.461089999 | 5.24945 | 0.84608 |
| ND-4-1 | 25.81599312 | 0.192316062 | 0.001832825 | 0.077558975 | 1.71217 | 0.18806 |
| ND-4-1 | 21.56311855 | 2.084545867 | 0.069734805 | 1.063588398 | 6.96157 | 2.01185 |
| ND-4-1 | 22.9577552 | 0.113560649 | 0.000258088 | 0.039442427 | 1.5514 | 0.11283 |
| ND-4-1 | 22.94632154 | 0.037708308 | 6.74982E-05 | 0.017931385 | 0.7756 | 0.0376 |
| ND-4-1 | 21.85217092 | 1.524878061 | 0.042485688 | 0.73227049 | 7.41579 | 1.48542 |
| ND-4-1 | 23.49890879 | 0.684508775 | 0.021642323 | 0.400352911 | 3.81166 | 0.63921 |
| ND-4-1 | 19.6023928 | 0.057748057 | 0.000405499 | 0.0285821 | 1.04982 | 0.0564 |
| ND-4-2 | 90.55035884 | 1.077992661 | 0.02453355 | 0.600412135 | 5.80445 | 1.05328 |
| ND-4-2 | 88.76466829 | 1.5015947 | 0.052057608 | 0.766666978 | 5.59333 | 1.28012 |
| ND-4-2 | 91.16773739 | 0.925275298 | 0.008962489 | 0.508609748 | 4.6188 | 0.90746 |
| ND-4-2 | 91.21827986 | 1.294010998 | 0.030262457 | 0.644751927 | 5.84806 | 1.23152 |
| ND-4-2 | 90.71192799 | 1.906508763 | 0.035498298 | 0.850317045 | 7.58661 | 1.84732 |
| ND-4-2 | 91.83510344 | 1.010326119 | 0.010572445 | 0.629424466 | 5.99721 | 1.00465 |
| ND-4-2 | 91.50392032 | 0.861108254 | 0.011073318 | 0.464478925 | 4.04789 | 0.84263 |
| ND-4-2 | 91.26408175 | 1.028773589 | 0.037825519 | 0.498946209 | 4.25878 | 0.93985 |
| ND-4-2 | 91.72845661 | 1.598965992 | 0.020466899 | 0.785422076 | 8.36852 | 1.58802 |
| ND-4-2 | 90.61303079 | 0.195683323 | 0.000811637 | 0.092750806 | 1.94946 | 0.19447 |
| ND-4-2 | 90.59224651 | 1.991873613 | 0.035967012 | 0.897302384 | 6.3135 | 1.96068 |
| ND-4-2 | 88.17677315 | 1.126906394 | 0.034128075 | 0.629888789 | 5.89174 | 1.08572 |
| ND-4-2 | 88.10383478 | 0.09738096 | 0.000158151 | 0.048231573 | 1.22929 | 0.09723 |
| ND-4-3 | 77.2546568 | 0.039683481 | 0.000523252 | 0.020280431 | 0.7056 | 0.03112 |
| ND-4-3 | 77.47647045 | 0.339772412 | 0.011516776 | 0.177699621 | 2.8232 | 0.24908 |
| ND-4-3 | 78.29640695 | 1.327303405 | 0.116245832 | 0.753322205 | 8.8043 | 0.98059 |
| ND-4-3 | 81.56827406 | 1.685906393 | 0.02391388 | 0.830818935 | 6.54105 | 1.63443 |
| ND-4-3 | 82.45595958 | 0.077926977 | 7.62047E-05 | 0.032770392 | 1.30821 | 0.07785 |
| ND-4-3 | 82.05262717 | 2.498555139 | 0.090731049 | 1.254734584 | 10.92167 | 2.3193 |
| ND-4-3 | 81.78543754 | 1.381640556 | 0.071628984 | 0.71948569 | 5.68888 | 1.04286 |
| ND-4-3 | 82.06642475 | 0.048392696 | 0.000469508 | 0.025643332 | 0.95544 | 0.04672 |
| ND-4-3 | 81.82869672 | 0.235950351 | 0.003634047 | 0.111454061 | 2.65905 | 0.23346 |
| ND-4-3 | 84.85686468 | 0.680689157 | 0.005967601 | 0.3719078 | 3.71776 | 0.66928 |
| ND-4-3 | 81.96596007 | 0.9254604 | 0.009126718 | 0.512388826 | 5.06874 | 0.91838 |
| ND-4-3 | 81.73878265 | 0.018386956 | 0.000127003 | 0.010226142 | 0.60227 | 0.01556 |
| ND-4-3 | 81.69941621 | 0.018278194 | 0.000129221 | 0.009184001 | 0.60261 | 0.01558 |
| ND-4-3 | 81.64653132 | 0.118649896 | 0.001940983 | 0.05869682 | 1.55751 | 0.09338 |
| ND-4-3 | 84.40733437 | 0.574878799 | 0.010418799 | 0.328874988 | 4.17401 | 0.56035 |
| ND-4-3 | 81.36273282 | 4.876942273 | 0.201863887 | 2.398637492 | 12.37604 | 4.38961 |
| ND-4-3 | 84.94293711 | 0.877167972 | 0.007464827 | 0.503751795 | 5.23272 | 0.87167 |
| ND-4-3 | 81.05957569 | 3.766046179 | 0.145698052 | 1.708258683 | 15.49162 | 3.48676 |
| ND-4-3 | 82.9758557 | 1.03541733 | 0.03773741 | 0.590498758 | 4.17412 | 0.87176 |
| ND-4-3 | 81.21089865 | 0.019032005 | 0.000183277 | 0.006928945 | 0.60244 | 0.01557 |
| ND-4-3 | 80.82970833 | 1.248295795 | 0.053056608 | 0.714542528 | 6.43766 | 1.12075 |
| ND-4-8 | 26.67857841 | 0.247635449 | 0.000911929 | 0.124285018 | 2.50153 | 0.24661 |
| ND-4-8 | 26.50889941 | 0.211795563 | 0.001288862 | 0.097175117 | 1.91961 | 0.20865 |
| ND-4-8 | 26.3756163 | 0.127687553 | 0.000963025 | 0.067663755 | 1.89266 | 0.12647 |
| ND-4-8 | 25.96022333 | 3.253574532 | 0.2439583 | 2.036906391 | 14.91508 | 2.99724 |
| ND-4-8 | 26.54309337 | 2.40781807 | 0.067390352 | 1.576627098 | 11.31648 | 2.36498 |
| ND-4-8 | 26.98481415 | 0.01274966 | 2.08744E-05 | 0.006448386 | 0.45 | 0.01266 |
| ND-4-8 | 28.60862519 | 0.145557176 | 0.000482426 | 0.074546141 | 2.33087 | 0.14542 |
| ND-4-8 | 27.20345445 | 0.432431312 | 0.004071202 | 0.230403667 | 3.65311 | 0.42996 |
| ND-4-8 | 27.70976797 | 0.088603735 | 0.000128986 | 0.045546486 | 1.53554 | 0.08851 |
| ND-4-8 | 26.7570875 | 1.001255758 | 0.018089244 | 0.598276012 | 5.18917 | 0.9738 |
| ND-4-11 | 85.73967085 | 0.29681295 | 0.001714324 | 0.096803165 | 2.62277 | 0.29508 |
| ND-4-11 | 84.68488455 | 0.406658039 | 0.00304915 | 0.202997752 | 3.23197 | 0.40574 |
| ND-4-11 | 84.27067451 | 0.88514205 | 0.013252414 | 0.49601321 | 4.38417 | 0.8483 |
| ND-4-11 | 81.18294169 | 0.839138703 | 0.014330533 | 0.515095254 | 5.80813 | 0.82988 |
| ND-4-11 | 81.10713616 | 0.055952925 | 0.000236942 | 0.026762535 | 1.03973 | 0.05532 |
| ND-4-11 | 80.83719953 | 4.238150729 | 0.092241551 | 2.918238132 | 17.67687 | 4.1861 |
| ND-4-11 | 81.24432583 | 1.262642777 | 0.046333025 | 0.684345411 | 5.08646 | 1.06959 |
| ND-4-11 | 80.64369769 | 0.70845312 | 0.00955387 | 0.325975049 | 4.1592 | 0.70072 |
| ND-4-11 | 81.12977526 | 0.018458006 | 1.08826E-05 | 0.010017662 | 0.6557 | 0.01844 |
| ND-4-11 | 81.12664977 | 0.018476232 | 1.48373E-05 | 0.010018404 | 0.6557 | 0.01844 |
| ND-4-11 | 81.09172236 | 1.23841134 | 0.005720271 | 0.559131294 | 4.8151 | 1.23563 |
| ND-4-11 | 81.14635533 | 0.148588157 | 0.00099873 | 0.077558388 | 1.6954 | 0.14753 |
| ND-4-11 | 80.12692574 | 1.375397131 | 0.018671985 | 0.759735275 | 5.74216 | 1.34624 |
| ND-4-11 | 80.46774887 | 0.388996886 | 0.003236463 | 0.153546998 | 2.5103 | 0.38729 |
